# Isopropyl Amino Acid Esters Ionic Liquids as Vehicles for Non-Steroidal Anti-Inflammatory Drugs in Potential Topical Drug Delivery Systems with Antimicrobial Activity

**DOI:** 10.3390/ijms232213863

**Published:** 2022-11-10

**Authors:** Joanna Klebeko, Oliver Krüger, Mateusz Dubicki, Paula Ossowicz-Rupniewska, Ewa Janus

**Affiliations:** 1 Department of Chemical Organic Technology and Polymeric Materials, Faculty of Chemical Technology and Engineering, West Pomeranian University of Technology in Szczecin, Piastów Ave. 42, 71065 Szczecin, Poland; 2 Department II Mathematics, Physics and Chemistry, Berliner Hochschule für Technik, Luxemburger Straße, 13353 Berlin, Germany; 3 Department of Inorganic Chemical Technology and Environment Engineering, Faculty of Chemical Technology and Engineering, West Pomeranian University of Technology in Szczecin, Piastów Ave. 42, 71065 Szczecin, Poland

**Keywords:** amino acid ionic liquids, non-steroidal anti-inflammatory drugs, antimicrobial activity, transdermal drug delivery

## Abstract

New derivatives of non-steroidal anti-inflammatory drugs were synthesized via conjugation with L-amino acid isopropyl esters. The characteristics of the physicochemical properties of the obtained pharmaceutically active ionic liquids were determined. It has been shown how the incorporation of various L-amino acid esters as an ion pair affects the properties of the parent drug. Moreover, the antimicrobial activity of the obtained compounds was evaluated. The proposed structural modifications of commonly used drugs indicate great potential for use in topical and transdermal preparations.

## 1. Introduction

Non-steroidal anti-inflammatory drugs (NSAIDs) represent one of the main therapeutic classes of most widely used and prescribed medications, commonly and extensively used for the treatment of pain and inflammation for their analgesic, antipyretic and anti-inflammatory properties. The group of NSAIDs consists of a large group of compounds with various chemical structures and selectivity, mostly having free carboxylic and enol groups [1,2].

The most common route of administration of NSAIDs is oral tablets, but they are also available as parenteral and topical formulations, which are especially useful for soft injuries and osteoarthritis pain therapy. However, the low solubility of these drugs in water is the limiting factor of their bioavailability and requires the use of high dosages to reach a therapeutic concentration in the blood system. Furthermore, the mechanism of action of NSAIDs, which inhibits mucosal prostaglandin production, may also cause both upper and lower gastrointestinal mucosal damage, especially when using a high dosage of the drug [3]. Moreover, more complications may occur, involving acute renal dysfunction and fluid and electrolyte disorders. Similarly, the acidic nature of these drugs may induce serious side effects involving an increased risk of ulceration, vomiting, perforations, and gastrointestinal bleeding [4,5].

Therefore, many efforts have been made to obtain derivatives of NSAID moieties with higher safety profiles to overcome these disadvantages. One of the common approaches is the administration of the corresponding prodrug, which is converted in vivo into an active drug form. The most common structural modification of the parent acid is the synthesis of ester derivatives, masking the free carboxylic acid group and improving the bioavailability of the active substance [6,7,8]. It is also possible to obtain so-called codrugs (or mutual prodrugs) consisting of two drugs linked together via covalent linkage in one molecule, demonstrating additive or synergic pharmaceutical effects [9,10]. In addition, the conjugation of natural alcohols and phenols having antioxidant properties (such as thymol, menthol, eugenol, and curcumin) with NSAID moieties were successfully obtained [11,12,13].

Another possibility is converting the active substance into salts, which increases the bioavailability and eliminates drawbacks resulting from the solid state of the active substance, such as limited solubility and polymeric conversion. It is well documented that salt formation allows derivatives to be obtained with a higher rate of absorption in comparison to the parent acid without changing its biological properties [14]. The most common derivatives in this group are sodium and potassium based compounds, obtained by the replacement of an acid proton with metal ions [15,16,17]. The lysine salts of ibuprofen and ketoprofen, with higher solubility and faster absorption after oral administration than free acid, are also known [18,19].

The prominent potential of improving drug bioavailability is assigned to using ionic liquids as novel drug formulation systems. The ionic liquids are organic salts consisting of asymmetric cations and anions, with increased distance between positive and negative charges disturbing the lattice packing and resulting in low melting points (below 100 °C). Drawing on the concept of tunable physicochemical properties, it is possible to manipulate them by pairing therapeutic ions with the proper counterion [15].

The transformation of the active pharmaceutical ingredient into the ionic liquids from (API-ILs) has shown great potential in drug development and delivery domains, especially in overcoming bioavailability, polymorphism, and stability issues associated with solid-state pharmaceuticals [20,21]. In terms of higher bioavailability, one of the most desirable goals is obtaining the API-based ILs with higher solubility in polar and nonpolar solvents compared to parent drugs. Especially, overcoming poor solubility in water and biological media is crucial since it limits the therapeutic utility of active substances [22]. The great increase in water solubility of salicylic acid was achieved by pairing it with various amino acid esters, resulting in salts miscible with water at any ratio [23]. The 60- and 120-fold increase in the aqueous solubility of ibuprofen, naproxen, and ketoprofen was evaluated for its imidazolium and phosphonium ionic liquids [24,25].

Transdermal and topical administration is a favorable strategy for avoiding systemic toxicity, bypassing the first-pass metabolism by delivering active substances directly into the systemic circulation, and avoiding erratic drug absorption through the gastrointestinal tract [26]. However, the crucial issues are enhancing active substance flux through the complex structure of the skin and lowering irritation and toxicity. The potential use of ionic liquids as ingredients in transdermal and topical drug delivery systems was the subject of recent studies. The main formulations in the development of API-ILs for this purpose consists of the combination of ionizable active substance and counterion or a combination of two different API moieties, one with acidic and the second with basic character [27,28,29,30]. In these terms, some NSAIDs were incorporated into an ionic liquid form to enhance transdermal or topical delivery. Yang et al. described the correlation of the physical properties of counterions, such as molecular weight, melting point temperatures, polarizability, and partition coefficient, impacting skin permeability of non-steroidal anti-inflammatory drugs based ILs [31]. Some studies have been performed describing the incorporation of functional ingredients. For example, lidocaine–ibuprofen ionic liquid indicated a faster local anesthesia effect and higher permeation rate through the artificial membrane than ibuprofen salts or conventional ionized lidocaine [32,33]. The transformation of ibuprofen, ketoprofen, and naproxen into choline-based ionic liquids was characterized by higher solubility in PBS aqueous solution. Moreover, incorporated further into bacterial nanocellulose, were proved to be suitable for further topical applications [34].

The amino acids constitute a promising class of biocompatible and nontoxic compounds, which were noticed as potential enhancers for transdermal delivery systems [35,36]. Moreover, amino acids are widely recognized in synthesizing prodrugs since they can facilitate membrane transport using peptide transporters and improve several pharmacological parameters, among others, solubility and permeability [37,38,39]. For example, the L-valine ester prodrugs of acyclovir, ganciclovir, and valacyclovir are already approved and effectively used to treat infection caused by herpes virus HSV-1 and HSV2, human cytomegalovirus and varicella zoster virus [40,41,42]. In addition, amino acids may also be considered candidates for synthesizing antimicrobial and antibiofilm agents [43,44].

Our previous studies were focused on assessing the impact of the chain length in the alkyl ester of the amino acid on the properties of the NSAID paired with amino acid ester cation [45,46]. In our previous work, we presented the leverage of using isopropyl esters of protein amino acids conjugated with ibuprofen to improve the permeation of an active substance through the skin. Moreover, we demonstrated that the conjugations of the L-valine alkyl ester moiety paired with ketoprofen and naproxen indicate low toxicity against a murine macrophage cell line [47,48], while salts of salicylic acid show potential as novel topical agents for chronic skin diseases by inhibiting the pro-inflammatory cytokine IL-6 in LPS-stimulated keratinocytes [49].

This research focuses on synthesizing the isopropyl ester of four amino acids (L-valine, L-isoleucine, L-threonine, and L-methionine) paired with selected active ingredients from the NSAIDs group: (*R*,*S*)-ibuprofen, (*R*,*S*)-ketoprofen, *S-*(+)-naproxen, and salicylic acid. We compare the influence of the structure of the amino acid moiety on the antimicrobial and physiochemical properties of obtained salts, being crucial for evaluating the bioavailability of pharmaceutical ingredients. We expand the evaluation of the potential usage of amino acid isopropyl ester-based ionic liquids in topical drug administration.

## 2. Results

The salts of four NSAIDs’ acids and amino acids isopropyl esters were obtained in accordance with the previously described three-step method, which was successfully used before for the synthesis of L-valine alkyl ester salts [45,46,47,48,49]. As a result, compounds were obtained with high yields (95–99%).

The identification of obtained compounds was based on the ^1^H and ^13^C-NMR, ATR-FTIR, and elemental analysis. The high purity of NSAID salts was confirmed by NMR and the content of individual elements (C, H, N, S, O). The detailed identification and description of the L-amino acid isopropyl ester salts of ibuprofen were presented in a previous publication [50].

The formation of the ionic structure was confirmed based on the presence of the protonated amino group on the ^1^H NMR spectra and carbonyl groups, both from the NSAID’s acid moiety and the amino acid part on ^13^C NMR spectra. All NMR results and comparisons of ^1^H and ^13^C-NMR spectra are presented in the Supplemental Material. Comparing ^1^H NMR spectra for ibuprofen salts, the chemical shift of the protons of NH_3_ group changes from 4.67 (L-methionine derivative) to 6.10 (L-threonine derivative), while for ketoprofen salts from 5.62 (for L-isoleucine derivative) to 6.44 (for L-methionine derivative), and naproxen salts from 4.97 (L-threonine derivative) to 5.83 (L-isoleucine derivative). In the case of salicylates, the broad signal from the protonated NH_3_ group is present in the range of about 7–9 ppm in NMR spectra.

In addition, the carbonyl carbon signal corresponding to the carboxylate anion of NSAIDs is shifted to a higher field of about 2 ppm (to about 179 ppm for ibuprofen salts and about 178 ppm for ketoprofen and naproxen derivatives) compared to the signal for this carbon in the unmodified acid (IBU: 181.18 ppm, KETO: 180.24 ppm, and NAP: 181.00 ppm). The exception occurs for all salicylates and all L-threonine derivatives, where this difference equals about 1 ppm.

Moreover, the ATR-IR analysis confirmed the presence of two characteristic absorption bands, visible at ca. 1600 and 1390 cm^−1^, which are assigned respectively to symmetric ν(COO^−^)_sym_ and asymmetric ν(COO^−^)_as_ stretching vibrations. The presence of the carboxylate anion COO^−^ is confirmed by the difference between the frequency of ν(COO^−^)_sym_ and ν(COO^−^)as values equal to above 200 cm^−1^ [51,52,53]. Also, the sharp absorption band of characteristic stretching vibrations C=O of the carboxylic acid group is observed in the range 1733–1742 cm^−1^, while for the unmodified acid broadband is observed for IBU at 1709 cm^−1^, KETO at 1692 cm^−1^, NAP at 1725 cm^−1^, and SA at 1653 cm^−1^. All recorded ATR-IR results and the detailed comparison of ATR-IR spectra for NSAIDs’ acids and their salts (Figures S26–S29) can be found in Supplementary Materials.

The comparisons of XRD patterns for selected NSAIDs’ acids and their amino acid isopropyl ester salts are presented in Supplementary Materials (Figures S51–S54). The last work also confirmed the crystalline nature of obtained ibuprofen salts [50]. In the case of [L-ThrOiPr] and [L-MetOiPr] ketoprofenates salts and [L-ThrOiPr][SA], which are semi-solid products, it was impossible to characterize the crystalline phase. The obtained XRD data also confirmed the partial crystalline nature of ketoprofen salts, also indicated by DSC analysis. It is noticeable that other salts exhibit characteristic diffraction peaks corresponding to diffractometric patterns of unmodified acid: ketoprofen, naproxen, and salicylic acid. However, the apparent sharp peaks usually occur at shifted 2θ value.

The description of the physical properties of the obtained salts included the determination of phase transition temperatures, thermal stability, specific rotation, solubility in water and selected organic compounds, and lipophilicity. Table 1 summarizes the specific and molar rotation values and the obtained results from the thermochemical analysis, where T_DSConset_ and T_DSCmax_ values are summarized, obtained from the first heating cycle (performed after conditioning step), and T_C_ was determined in the first cooling cycle. 

**Figure 1:**
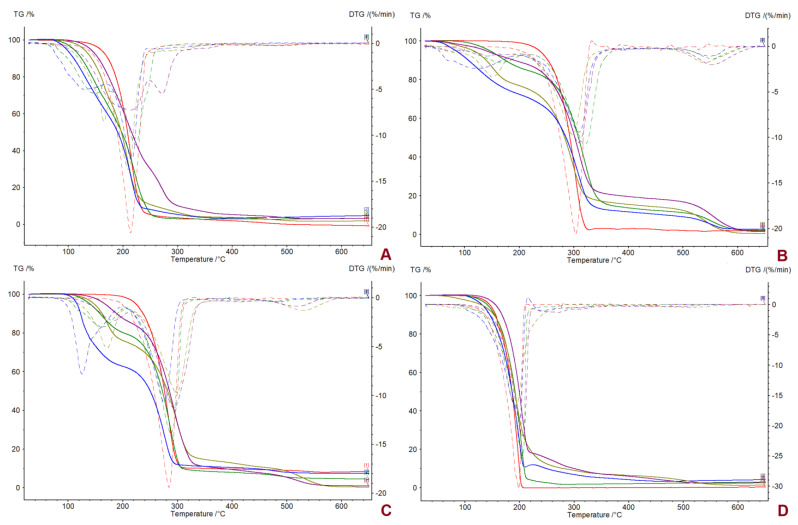
The TG (straight line) and DTG (dotted line) curves of ibuprofen (**A**), ketoprofen (**B**), naproxen (**C**) and salicylic acid (**D**) and their salts, from the top: unmodified acid (red), salts of [L−ValOiPr](blue), salts of [L−IleOiPr] (green), salts of [L−ThrOiPr] (yellow-green), and salts of [L−MetOiPr] (purple).

**Figure 2:**
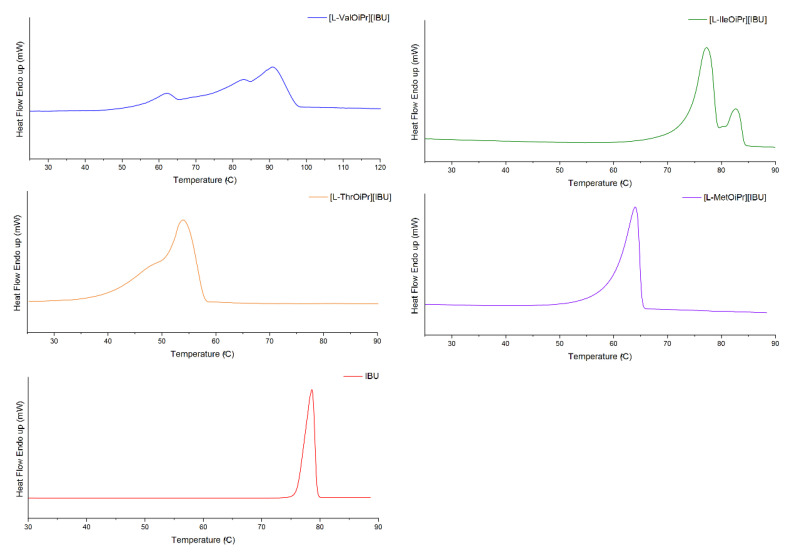
The DSC curves of first heating cycle of ibuprofen and ibuprofen salts: [L−ValOiPr][IBU] (blue), [L−IleOiPr][IBU] (green), [L−ThrOiPr][IBU] (orange), [L−MetOiPr][IBU] (purple), IBU (red).

**Figure 3:**
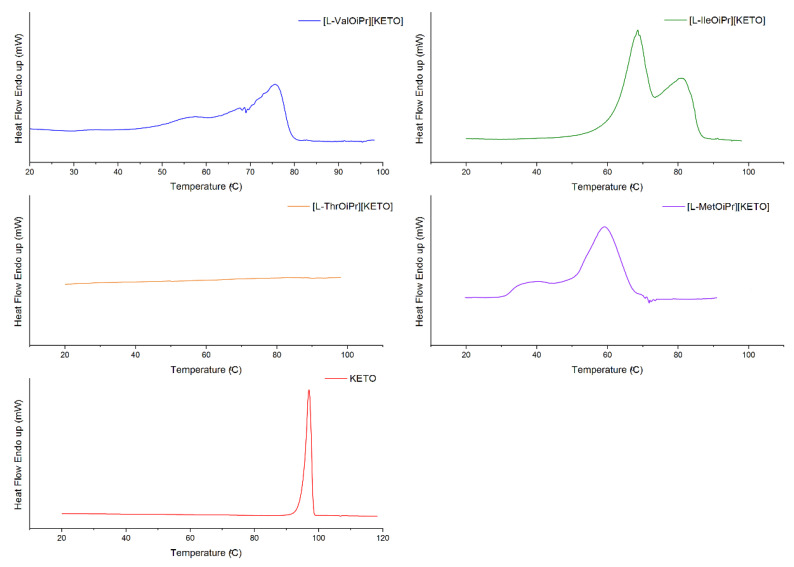
The DSC curves of first heating cycle of ketoprofen and ketoprofen salts: [L−ValOiPr][KETO] (blue), [L−IleOiPr][KETO] (green), [L−ThrOiPr][KETO] (orange), [L−MetOiPr][KETO] (purple), KETO (red).

**Figure 4:**
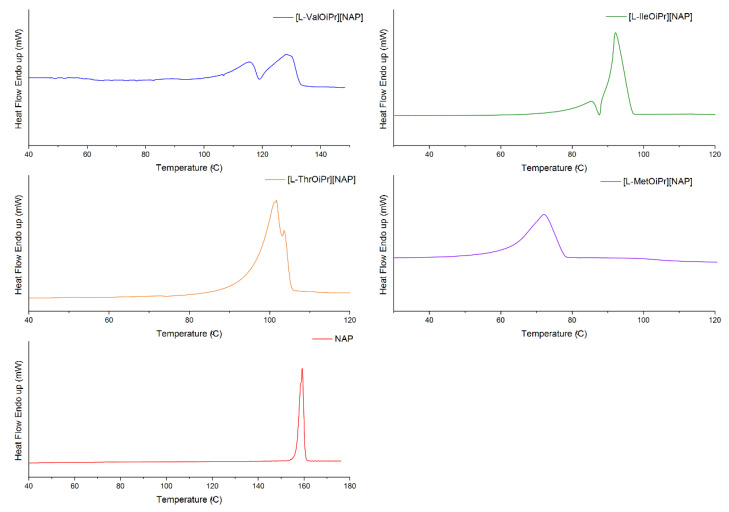
The DSC curves of first heating cycle of naproxen and naproxen salts: [L−ValOiPr][NAP] (blue), [L−IleOiPr][NAP] (green), [L−ThrOiPr][NAP] (orange), [L−MetOiPr][NAP] (purple), NAP (red).

**Figure 5:**
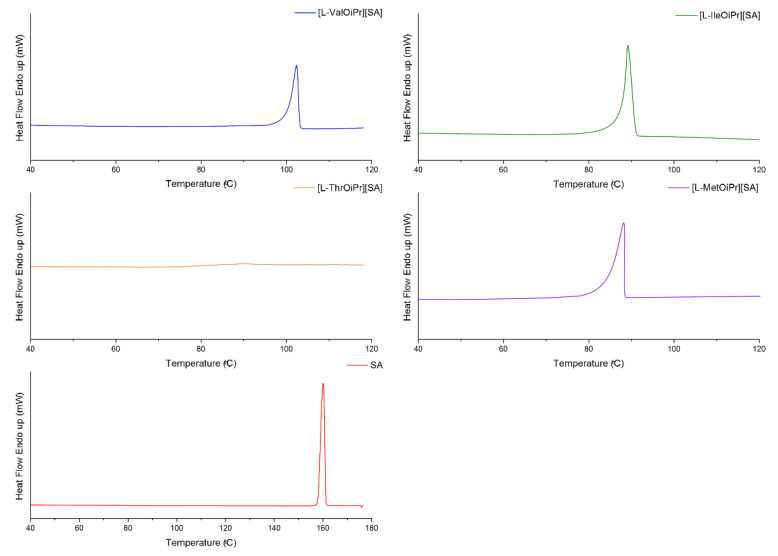
The DSC curves of first heating cycle of salicylic acid and salicylic acid salts: [L−ValOiPr][SA] (blue), [L−IleOiPr][SA] (green), [L−ThrOiPr][SA] (orange), [L−MetOiPr][SA] (purple), SA (red).

The thermal stabilities of the obtained NSAIDs isopropyl ester amino acid salts were investigated. The TG and DTG curves are included in the Supplementary Material. Comparison of thermal degradation and the maximum rate of mass loss is presented in Figure 1 and the DSC curves determined from the first heating cycle are compared in Figure 2, Figure 3, Figure 4 and Figure 5. The beginning of thermal degradation was evaluated based on the value of onset of the TG curve, which occurs in the first phase of the process. The values of maximum mass loss rate (T_max_) were comparable for isopropyl esters salts and parent drugs. The decomposition temperature (T_onset_) for isopropyl amino acid salts is lower in comparison to parent drug moiety, except [L-MetOiPr][SA] (T_onset_ = 161.1 °C), indicating a higher thermal stability than unmodified salicylic acid (T_onset_ = 146.2 °C). Conjugates of L-methionine isopropyl ester mostly demonstrate the highest thermal stability, while the least stable are salts of L-valine isopropyl esters.

For DSC analysis, a conditioning step was performed prior to the recording of heating/cooling cycles in order to eliminate the influence of contaminations, such as residual chloroform, and also to cancel the thermal history of the materials under study [54]. The DSC curves for NSAIDs’ salts were also recorded with a conditioning sequence of the sample and are available in Supplementary Material. In case of slow returning to the solid state compounds (salts of ketoprofen, L-threonine isopropyl ester naproxenate and L-threonine isopropyl ester ibuprofenate), the complete registration of phase transition data was collected after 24 h before conditioning step. However, no difference was observed in the phase transition study between T_DSConset_ and T_DSCmax_ recorded in conditioning and the first heating cycle. In addition, none of these salt samples reverse to the same state upon the cooling cycle.

The melting point data evaluated from DSC analysis are based on both registered T_DSConset_ and T_DSCmax_ values as registered peaks are mostly broad. In the case of compounds of [L-ThrOiPr] ester paired with ketoprofen and salicylic acid, which are in the amorphous state, no melting points could be evaluated. In general, the obtained NSAIDs showed lower melting points compared to the parent acid. The L-threonine derivatives had the lowest melting point temperatures of the studied salts of NSAID acids (T_DSConset_ = 44.62 °C and T_DSCmax_ = 91.97 °C for [L-ThrOiPr][IBU], and T_DSConset_ = 87.16 °C and T_DSCmax_ = 91.97 °C for [L-ThrOiPr][NAP]), while the salts of amino acids with branched chain, i.e., L-valine and L-isoleucine, showed the highest melting points. It is expected that the drugs characterized with lower melting points tend to achieve greater skin penetration due to the higher concentration gradient between the matrix and the skin, which allows it to pass through the skin more rapidly [28]. Based on the observed peak maximum temperature values, all synthetized amino acid isopropyl ester salts, with the exception of [L-ValOiPr][NAP] and [L-ValOiPr][SA], have melting points below 100 °C. Based on determined melting points and the confirmed ionic structure, the synthetized amino acid isopropyl ester salts and selected acid from NSAIDs group can be classified as ionic liquids [55].

No crystallization peaks were observed for the conjugations of [L-ThrOiPr] ester with NSAIDs acids moieties, as well as for all the ketoprofen salts. In addition, the observed characteristic broad endothermic peak in the first heating cycle may also suggest the partial crystallization of these compounds. In the case of other salts being in the solid state, the samples indicate the high thermal stability, which is proved by the almost identical temperatures of melting and crystallization observed in the second heating and cooling cycle recorded in the DSC measurements. With the exception of ketoprofen salts, and compounds in the amorphous state, the conditioning step suggests the existence of more stable phases of studied L-amino acid isopropyl ester derivatives. Moreover, in case of L-valine and L-isoleucine salts paired with ibuprofen and naproxen moieties, two different phases were observed, which were initially assigned to two overlapped peaks in the conditioning transition. Also, the characteristic shape of peaks registered in the cooling transition for [L-ValOiPr][IBU] may also suggest the occurrence of eutectic impurity, while in the case of other compounds only single broad peaks were observed.

**Figure 6:**
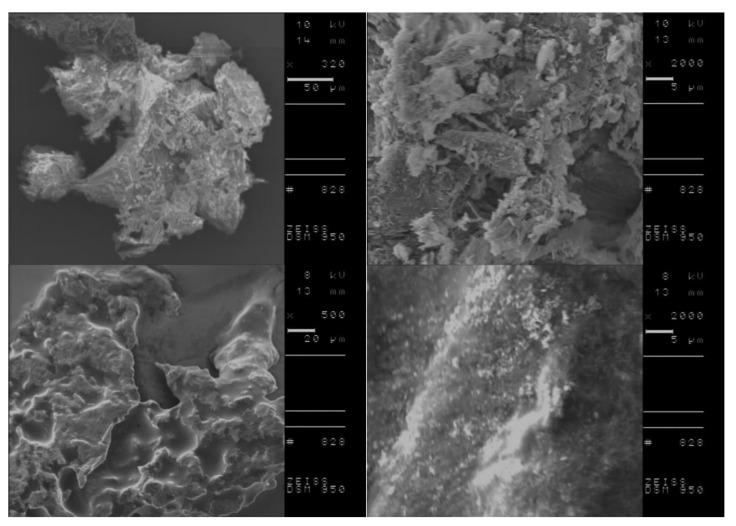
The SEM micrographs of [L-IleOiPr][IBU]; in the top row: before the conditioning step and in the bottom row: after the conditioning step; on the right: enlarged 2000 times SEM image; on the left side lower magnification (enlarged 320 and 500 times respectively).

**Figure 7:**
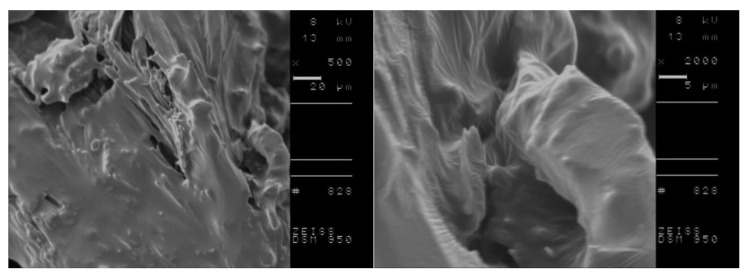

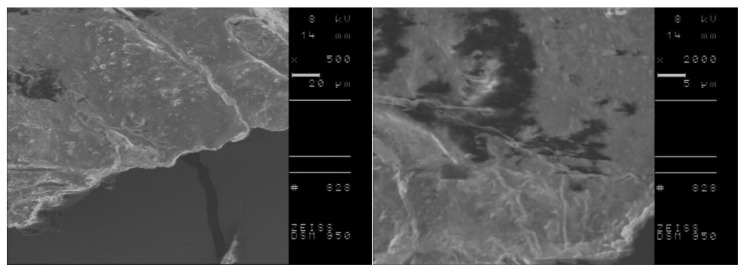
The SEM micrographs of [L-IleOiPr][KETO]; in the top row: before the conditioning step and in the bottom row: after the conditioning step; on the right: enlarged 2000 times SEM image; on the left side lower magnification (enlarged 500 times respectively).

**Figure 8:**
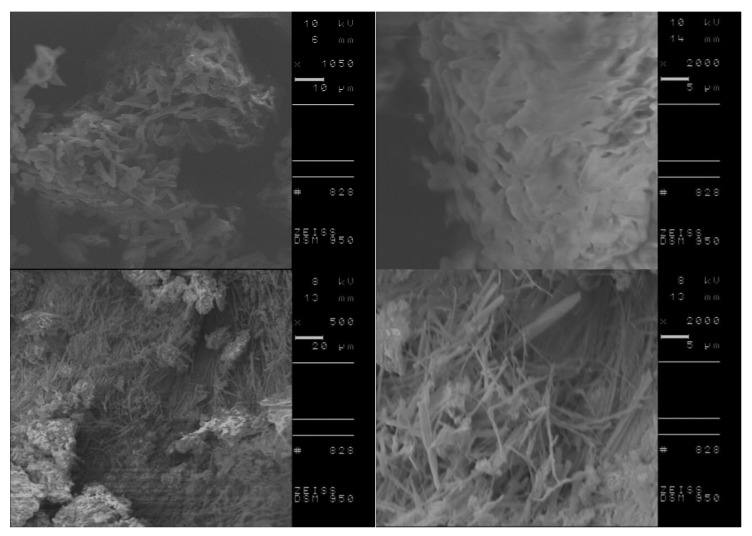
The SEM micrographs of [L-MetOiPr][NAP]; in the top row: before the conditioning step and in the bottom row: after the conditioning step; on the right: enlarged 2000 times SEM image; on the left side lower magnification.

**Figure 9:**
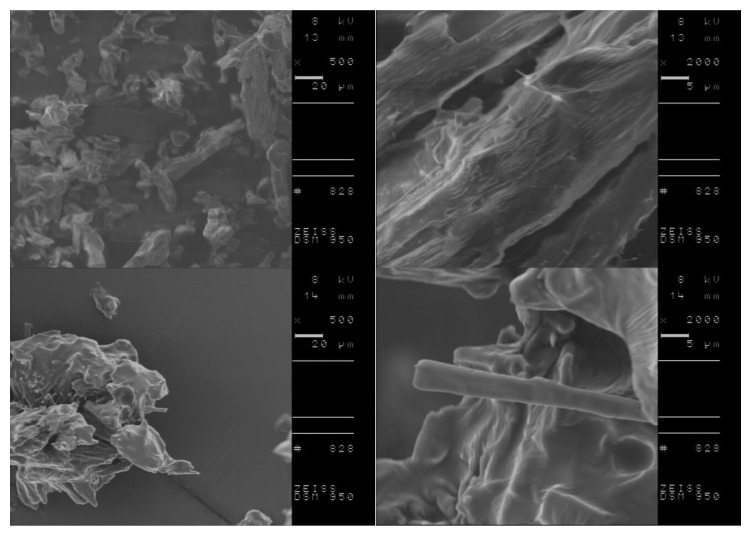
The SEM micrographs of [L-MetOiPr][SA]; in the top row: before the conditioning step and in the bottom row: after the conditioning step; on the right: enlarged 2000 times SEM image; on the left side lower magnification (enlarged 1050 and 500 times respectively).

DSC analysis of L-amino acid isopropyl salts including the conditioning step indicated the possibility of more stable and the in some cases, the occurrence of partial crystallization. Therefore, we investigated the morphology of selected compounds, before and after performing the conditioning step to obtain more detailed information on crystallite characteristics of the studied materials. Based on obtained results from DSC measurements, [L-IleOiPr][IBU], [L-IleOiPr][KETO], [L-MetOiPr][NAP] and [L-MetOiPr][SA] were chosen as the representative compounds for further SEM investigations. The performed SEM measurements revealed two different crystal morphologies of the studied ionic liquids before and after the conditioning step. The comparison of the recorded SEM micrographs is shown in Figure 6, Figure 7, Figure 8 and Figure 9.

Canceling the thermal history of those analyzed materials clearly results in the modification of crystalline structure, which is observed as formation of other types of agglomerates. For example, the powder specimen of [L-IleOiPr][IBU], which is composed of particles with different shapes and porous surfaces (Figure 6, the top row), shows larger agglomerates after conditioning (Figure 8, the bottom row). In the case of [L-IleOiPr][KETO], a transformation from a semi-solid to an amorphous state was observed (Figure 7). On the other hand, the [L-MetOiPr][NAP] sustained the crystalline form with agglomerates of cylindrical-shaped particles, which transform after conditioning into smaller needle-shaped crystals (Figure 8). Similar morphological differences were observed in the case of the agglomerates of [L-MetOiPr][SA] (Figure 9), where smaller crystalline structures can be observed after conditioning. This may cause lower T_DSConset_ and T_DSCmax_ values observed in heating melting peaks registered in DSC analysis. The registered DSC curves for selected salts are available in Supplementary Material (Figures S66, S70, S76 and S80).

Due to the optical activity of the obtained salt’s amino acids, the specific and molar rotation were determined. In the case of the rest components in the role of the anion part, only *S*-(*+*)-naproxen also has optical activity. Ibuprofen and ketoprofen used for this study are racemic mixtures, and salicylic acid contains no asymmetric carbon in the molecule’s structure. The obtained values are summarized in Table 1. The specific rotation of salts depends on the type of amino acid in the cationic part. The value of specific rotation may be presented in descending order: [L-IleOiPr] > [L-ValOiPr] > [L-MetOiPr] > [L-ThrOiPr], except for [L-MetOiPr][KETO], which indicates the highest optical activity in a group of ketoprofenates salts. Moreover, in most cases, the value of the specific rotation of obtained salts is lower than that of the starting amino acids, resulting in a lower share of the amino acid in the total mass of the whole molecule. In addition, the direction of specific rotation of the obtained components sustained the direction of optical rotation of the proper amino acid. The mentioned occurrence is not noticeable in the case of naproxenate salts since both anion and cation have optical activity.

The solubility in conventional organic solvents was determined according to Vogel’s methodology and summarized in Table 2. Based on the value of the empirical polarity parameter (ET(30)), solvents are ranked with decreasing polarity. The compounds were characterized in accordance with established categories: soluble (100 mg of the compound is dissolved in 1 cm^3^), partly soluble (33–100 mg of the compound is dissolved in 1 cm^3^), or insoluble (less than 33 mg of the compound is dissolved in 1 cm^3^).

In general, obtained salts indicate comparable solubility to parent drugs in ethanol, DMSO, dichloromethane, and chloroform. Compared to unmodified salicylic acid, its salts with isopropyl esters of amino acids are characterized by improved solubility in chloroform. In addition, unlike pure ketoprofen, ketoprofenates are soluble in toluene. Similarly, obtained naproxenates show better solubility in toluene than the parent acid, except for [L-ValOiPr][NAP]. None of the examined salts were soluble in nonpolar hexane.

In our previous study, we demonstrated the influence of the elongation of the alkyl chain in the amino acid ester cation on the solubility of ibuprofen derivatives. This study aims to compare the influence of amino acid structure on the physicochemical properties of NSAID salts with the isopropyl ester of amino acids. Since the obtained salts are intended to be used in drug delivery systems, demonstrating solubility in aqueous solutions comparable to physiological conditions is crucial. Therefore, the solubility of NSAIDs and their salts was determined in deionized water and two buffers at pH 5.4 and 7.4 to represent similar pH values of the skin surface and the skin’s deep layers. The obtained results saturation concentration of the salts was also expressed as the concentration of an active substance (g_AS_) and summarized in Table 3. The obtained results are consistent with the results described in previous works.

As can be seen, converting each acid moiety into amino acid isopropyl ester salts affects the solubility of the drug in water markedly, and in both used phosphate buffers. The typical relationship between solubility and increasing pH was observed: saturated concentrations of the active substances and their salts in the buffer of pH 7.4 were slightly higher than the value of the saturated concentration in deionized water and the pH 5.4 buffer. In the previous work, we described the dependence of increasing alkyl chain length, alkyl in the amino acid part, and the ester part on the solubility of ibuprofen derivatives [45]. Also, in this study, the structure and hydrophobicity of starting amino acids alter the solubility of active substances in salt form. The highest values were obtained for L-threonine salts in each group of obtained compounds, while the lowest was for L-methionine salts. In general, the increase in solubility of active substances in water and both used phosphate buffers follow modifications of the amino acid in the ester part in the row: [L-Met] < [L-Val] < [L-Ile] < [L-Thr].

Since the skin consists of both hydrophobic and hydrophilic layers (stratum corneum composed of different lipids and viable epidermis, respectively), the permeation through the skin into the systemic circulation is achievable only for an active substance with suitable solubility in the oil and the water phase. In this term, the n-octanol-water partition coefficient (logP) is considered one of the indicative parameters for evaluating drug permeability [28].

The values of logP determined using the shake flask method are summarized in Table 4.

All of the obtained salts showed positive logP values. Except for salicylic acid derivatives, L-amino acid isopropyl esters compounds demonstrate lower logP than corresponding unmodified acid, indicating the formation of salts with higher hydrophilicity. As can be seen, the obtained results of the partition coefficient match the evaluated values of saturated concentration in deionized water. Therefore, the increase in lipophilicity of the active substance was ranked in the following order of modifications of amino acid: [L-Thr] < [L-Ile] < [L-Val] < [L-Met].

The modification of the intermolecular packing of drug moieties and their polarity, and therefore generation of proton transfer, is widely used for obtaining API-ILs. The mechanism of the ion-pair strategy is based on the difference in the pK_A_ between active substance and counterion (ΔpK_A_ > 3) [28,57]. Cruz-Cabeza described the dependence of ionized and non-ionized forms of pharmaceuticals on the ΔpK_A_ value and demonstrated that the acid and base ionized into acid–base complexes are observed exclusively for ΔpK_A_ > 4 [58].

The determined values of pKa for unmodified acids from NSAID groups were as follows: 4.65 for ibuprofen, 4.11 for ketoprofen, 4.32 for naproxen, and 3.18 for salicylic acid. The obtained pK_B_ values for amino acid isopropyl esters and calculated values of logKs for obtained salts are summarized in Table 5. Calculated differences, ΔpK_A_, between pK_A_ unmodified acids from NSAID groups and L-amino acid isopropyl esters were higher than 5, which proves the formation of ionized acid–base complexes. Moreover, based on calculated log values (K_S_), the predicted degree of salt formation would exceed 99% [59].

The determined values of pKa for unmodified acids from NSAID groups were as follows: 4.65 for ibuprofen, 4.11 for ketoprofen, 4.32 for naproxen, and 3.18 for salicylic acid. The obtained pK_B_ values for amino acid isopropyl ester and calculated values of logKs obtained salts are summarized in Table 5. Each calculated difference ΔpK_A_ between pK_A_ unmodified acids from NSAID groups and L-amino acid isopropyl esters was higher than 5, which proves the formation of ionized acid–base complexes. Moreover, based on calculated log values (K_S_), the predicted degree of salt formation would exceed 99% [59].

The antibacterial activity of acids from NSAIDs and their amino acid isopropyl ester salts was evaluated using the disc diffusion method. The results are summarized in Table 6, Table 7 and Table 8. The gram-negative bacteria *Escherichia coli* used in the present research was reported as one of the multidrug-resistant, extensive drug-resistant, and pan-drug-resistant pathogens [60]. *Staphylococcus epidermidis* forms a part of skin flora as a commensal organism and is also found in the mucous membrane of animals and may penetrate the epithelial barriers of the human body. Therefore, it is one of the major causes of nosocomial infections and one of the major blood culture contaminants [61]. The other bacteria, *M. Luteus* is considered commensal to human skin and upper respiratory tract. However, despite general safety, it may serve as an initial colonizer for more dangerous microorganisms on the skin [62,63].

The antibacterial mechanism mode of action of NSAIDs differs according to the species and may be indicated by their ability to inhibit DNA synthesis, prevent DNA replication, and repair the bacterial membrane, or they may affect the integrity of the cytoplasmic membrane and cause further damage [64]. However, the inhibition of microbial growth of NSAIDs is microorganism dependent [65], and it is observed the above concentrations are possible to achieve in the blood plasma upon oral administration. The topical administration of NSAIDs may overcome this disadvantage as the localized treatment method in the area of skin inflammation [66,67]. The obtained results revealed that the inhibition against studied bacteria follows the presence of L-amino acid isopropyl ester in NSAID salts and is not only the result of the concentration of an active substance in the compound. The presence of L-amino acid isopropyl ester indicated a significant growth inhibition against gram-negative bacteria effect in comparison to the parent drug. A similar effect was observed in the work of Obad et al., where ibuprofen lysine showed higher antimicrobial activity in comparison to pure ibuprofen [67]. However, the susceptibility of each bacterium towards the conjunction of L-amino acids with active substances varies. In general, the gram-positive bacteria, having thick peptidoglycan outer layers, absorb substances easily from the surrounding environment, had lower resistance against the salts under study. In contrast to gram-negative *E. coli*, cells are surrounded by a complex outer membrane containing lipopolysaccharides, playing many functional roles in detoxification and drug resistance [68].

In the case study using *E. coli*, the derivatives of [L-MetOiPr] ester showed the highest antimicrobial effect compared to the parent drug. In contrast, no effect was observed for L-threonine derivatives. Moreover, the L-methionine derivative was the only naproxen salt with a susceptibility effect. It can be described that *E. coli* bacteria export L-threonine through the efflux pumps coded amongst others by the rhtC gene. The overexpression of rhtC was proved to increase resistance to externally supplied L-threonine, further reducing the accumulation of metabolites derived from threonine. The effect leads to a three-fold increase in exported L-threonine in the modified production strain [69,70]. This mechanism can explain the lack of the susceptibility effect for L-threonine salts against studied bacteria. Therefore, the inhibition zone observed for [L-ThrOiPr][SA] is a result of the presence of salicylic acid in the compound, which was only among the studied unmodified NSAIDs that indicated antimicrobial activity against *E. coli*. Moreover, the derivatives of salicylic acids were characterized by the highest values of logP (1.610–1.1784) among all synthesized salts. The optimal lipophilicity may facilitate penetration through the cell wall of the bacteria, which consists of hydrophobic lipid layers. Thus, the salicylate salts are characterized by the highest inhibitory effect, which results in synergistic antibacterial activity.

In contrast, the inhibit0ry effect was evaluated for ibuprofen against *S. epidermidis* in all tested concentrations, while salicylic acid was from 400 mg cm^−3^. Obtained results for L-amino acids revealed that the presence of amino acid isopropyl part ester increases antibacterial activity only when paired with naproxen or the ketoprofen moiety. However, no inhibition zone was observed for [L-ThrOiPr][KETO] and [L-ThrOiPr][NAP]. For ibuprofen and salicylic acid salts, the antimicrobial effect is similar or lower (for [L-MetOiPr][IBU] and [L-MetOiPr][SA]) in comparison to the parent drug. Beavers et al. described that the Msr enzymes protect bacteria of the *Staphylococcus* group from some types of oxidative stress induced by sulfur-containing amino acids, such as cysteine or methionine. The specialized defense mechanism based on proper enzymes can therefore control the resistance of *S. epidermidis* to L-methionine derivatives [71].

The susceptibility of another gram-positive bacteria, *M. Luteus,* towards both unmodified acids from the NSAIDs group and their L-amino acid isopropyl ester salts were observed. In general, the conjugation with L-amino acid ester did not affect the increase in inhibition effect. Surprisingly, the antimicrobial effect against *M. Luteus* was observed for [L-ThrOiPr][SA] in all tested concentration ranges, while for the parent drug only for the highest studied concentration range from 400 mg cm^−3^.

The supplementation with a single amino acid in excessive amounts may cause a transient auxotrophy for other amino acids, which in turn results in a reduced growth rate and reduced ability to synthesize them. The valine biosynthetic pathway of *M. luteus* is associated with conjugated reactions with the leucine production pathway. Thus, Lichev et al. suggested the additional supplementation of branched amino acids, such as valine or isoleucine, affects transformability on a transcriptional and post-transcriptional level by inhibition of biosynthesis of leucine and the same decrease in the transcription of the comEA/EC gene, which is responsible for receptor processes but also plays a fundamental role in transport inside the cell [72,73,74]. This mechanism may explain this study’s low antimicrobial activities of isopropyl esters of branched amino acids.

## 3. Materials and Methods

### 3.1. Synthesis of Amino Acid Isopropyl Ester Salts

**Figure 10:**
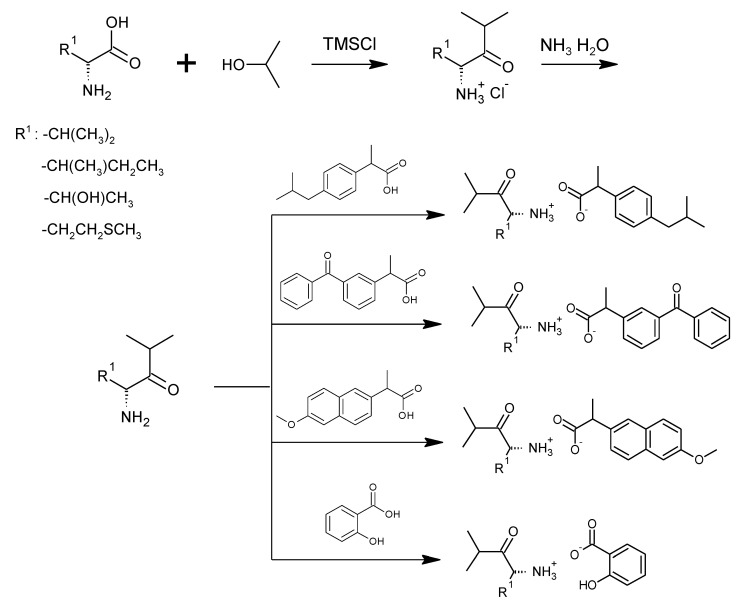
General synthesis pathway of L-amino acid isopropyl ester salts of selected acids from the NSAIDs group.

For the synthesis of all studied salts, we used the previously described three-step method [45,46], which was also successfully used for obtaining the salts of the L-valine alkyl esters paired with ketoprofen, naproxen, and salicylic acid [47,48,49]. The detailed procedure of synthesis of hydrochlorides, esters, and salts is described in the Supplementary Material. The general scheme of the synthesis is presented in Figure 10.

In the first step, the amino acid was submitted to the esterification reaction using a chlorinating agent acting as a catalyst in the presence of excess isopropanol as the reaction medium. Trimethylsilyl chloride was used in molar excess 1:1.5 (AA/TMSCl). TMSCl was chosen for this synthesis instead of SOCl_2_, which is more reactive but has higher toxicity and generates sulfur oxides as by-products. In the next step, synthesized hydrochlorides were neutralized by a base (25% ammonia solution) to obtain amino acid alkyl esters. In the final step, amino acid alkyl ester was obtained and reacted with an equimolar amount of acid from the NSAIDs group.

[L-IleOiPr][KETO]–L-isoleucine isopropyl ester ketoprofenate


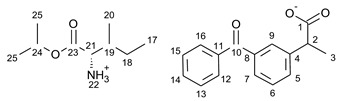


^1^H NMR (400 MHz, CDCl_3_) δ [ppm]: 7.83–7.75 (m, 3H, H16, H12, H7), 7.65 (d, J_9,8_ = 7.7, 1H, H9), 7.62–7.52 (m, 2H, H14, H13), 7.47 (t, J_5,6_ = 7.7 Hz, 2H, H15, H5), 7.41 (t, J_6,5_ = 7.7 Hz, 1H, H6), 5.62 (s, 3H, H22), 5.09–4.99 (m, 1H, H24), 3.79–3.69 (m, 1H, H2), 3.44 (d, J_19,20_ = 4.4 Hz, 1H, H21), 1.84–1.71 (m, 1H, H19), 1.50 (d, J_3,2_ = 7.2 Hz, 3H, H3), 1.47–1.32 (m, 1H, H18′), 1.25–1.28 (2d, 6H, H25, H26), 1.21–1.13 (m, 1H, H18″), 0.94–0.80 (m, 6H, H17, H20). ^13^C NMR (100 MHz, CDCl_3_) δ [ppm]: 196.64 (C10), 178.35 (C1), 173.48 (C23), 141.60 (C8), 137.74 (C11), 137.56 (C4), 132.45 (C5), 131.74 (C14), 130.11 (C12/16), 129.34 (C9), 128.82 (C6), 128.40 (C7), 128.29 (C13/15), 68.73 (C24), 58.16 (C21), 45.95 (C2), 38.54 (C19), 24.88 (C18), 21.81 (C26/25), 18.52 (C3), 15.36 (C20), 11.74 (C17). FT–IR: ν (ATR): 2970, 2935, 2877, 2641, 2178, 2168, 1736, 1654, 1608, 1577, 1528, 1464, 1446, 1424, 1411, 1383, 1360, 1317, 1307, 1289, 1278, 1224, 1174, 1145, 1105, 1074, 1061, 1026, 1000, 971, 950, 905, 877, 842, 825, 813, 797, 784, 718, 708, 691, 658, 641, 611, 591, 560, 520, 484, 429 cm^–1^. UV–Vis (EtOH): λ_max_ = 208, 254 nm. Elemental analysis: Calc (%) for: C_25_H_33_NO_5_ (427.533 g/mol) C (70.23), H (7.78), N (3.28), O (18.71), found: C (70.19), H (7.61), N (3.15), O (18.64).

[L-ThrOiPr][KETO]–L-threonine isopropyl ester ketoprofenate


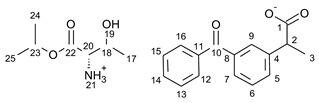


^1^H NMR (400 MHz, CDCl_3_) δ [ppm]: 7.86–7.70 (m, 3H, H16, H12, H7), 7.66–7.51 (m, 3H, H9, H14, H13), 7.47 (t, J_5,6_ = 2H, H5, H15), 7.38 (t, J_6,5_ = 7.7 Hz, 1H, H6), 5.72 (s, 4H, H19, H21), 5.09–4.98 (m, 1H, H23), 4.04–3.92 (m, 1H, H18), 3.77–3.65 (m, 1H, H2), 3.36 (d, J_20,21_ = 5.3 Hz, 1H, H20), 1.48 (d, J_3,2_ = 7.2 Hz, 3H, H3), 1.27–1.18 (2d, 9H, H25, H24, H17). ^13^C NMR (100 MHz, CDCl_3_) δ [ppm]: 196.80 (C10), 179.16 (C1), 171.53 (C22), 141.85 (C8), 137.70 (C11), 137.47 (C4), 132.52 (C5), 131.83 (C14), 130.15 (C12/C15), 129.28 (C9), 129.26 (C6), 128.78 (C7), 128.36 (C13), 128.31 (C15), 69.63 (C23), 67.52 (C20), 59.51 (C18), 46.26 (C2), 21.70 (C25), 21.66 (C24), 19.96 (C3), 18.61 (C17). FT–IR: ν (ATR): 3062, 3033, 2977, 2934, 2875, 2368, 1735, 1699, 1654, 1651, 1596, 1576, 1557, 1505, 1447, 1436, 1386, 1358, 1318, 1281, 1224, 1179, 1143, 1101, 1058, 999, 965, 954, 913, 897, 819, 753, 719, 702, 664, 641, 547, 508, 502, 498, 493, 484, 472, 466, 461, 458, 447, 444, 435, 430, 420, 408, 404., 402 cm^–1^. UV–Vis (EtOH): λ_max_ = 204, 254 nm. Elemental analysis: Calc (%) for: C_23_H_29_NO_6_ (415.479 g/mol) C (66.49), H (7.04), N (3.37), O (23.11), found: C (66.40), H (7.00), N (3.31), O (23.09).

[L-MetOiPr][KETO]–L-methionine isopropyl ester ketoprofenate


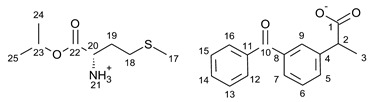


^1^H NMR (400 MHz, CDCl_3_) δ [ppm]: 7.81–7.76 (m, 3H, H16, H12, H7), 7.65 (d, J_9,8_ = 7.4 Hz, 1H, H9), 7.60–7.53 (m, 2H, H14, H13), 7.47 (t, J_5,6_ = 7.7 Hz, 2H, H5, H15), 7.43 (t, J_6,5_ = 7.7 Hz, 1H, H6), 6.44 (s, 3H, H21), 5.09–4.98 (m, 1H, H23), 3.81–3.71 (m, 1H, H20), 3.71–3.63 (m, 1H, H9), 2.62–2.49 (m, 2H, H18), 2.11–1.98 (m, 4H, H17, H19′), 1.95–1.81 (m, 1H, H19″), 1.51 (d, J_3,2_ = 7.2 Hz, 3H, H3), 1.29–1.15 (2d, 6H, H24, H25). ^13^C NMR (100 MHz, CDCl_3_) δ [ppm]: 196.62 (C10), 178.73 (C1), 173.54 (C22), 141.22 (C8), 137.78 (C11), 137.49 (C4), 132.51 (C5), 131.74 (C14), 130.12 (C12/C16), 129.32 (C9), 128.96 (C6), 128.46 (C7), 128.31 (C13/C15), 69.21 (C23), 52.83 (C20), 45.76 (C2), 32.68 (C19), 30.08 (C18), 21.76 (C25), 21.74 (C26), 18.43 (C3), 15.27 (C17). FT–IR: ν (ATR): 3059, 2980, 2921, 2872, 2374, 1735, 1701, 1654, 1617, 1596, 1577, 1481, 1447, 1386, 1375, 1359, 1317, 1281, 1216, 1179, 1145, 1103, 1074, 1026, 999, 965, 954, 902, 881, 856, 819, 750, 719, 701, 666, 642, 519, 483, 454, 440, 425, 415, 405 cm^–1^. UV–Vis (EtOH): λ_max_ = 204, 254 nm. Elemental analysis: Calc (%) for: C_24_H_31_NO_5_S (445.572 g/mol) C (64.69), H (7.01), N (3.14), O (17.95), S (7.20), found: C (64.53), H (7.12), N (3.05), O (17.84), S (7.11).

[L-IleOiPr][NAP]–L-isoleucine isopropyl ester naproxenate


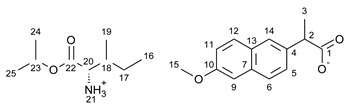


^1^H NMR (400 MHz, CDCl_3_) δ [ppm]: 7.69–7.61 (s+d, 3H, H8, H6, H11), 7.41 (d, J_10,11_ = 8.5 Hz, 1H, H10), 7.14–7.05 (s+d, 2H, H13, H5), 5.83 (s, 3H, H20), 5.09–4.95 (m, 1H, H22), 3.89 (s, 3H, H14), 3.83–3.73 (m, 1H, H2), 3.40 (d, J_19,14_ = 4.4 Hz, 1H, H19), 1.80–1.69 (m, 1H, H17), 1.53 (d, J_3,2_ = 7.2 Hz, 3H, H3), 1.42–1.31 (m, 1H, H16′), 1.28–1.16 (m, 6H, H24, H23), 1.18–1.07 (m, 1H, H16″), 0.90–0.79 (2d, 6H, H18, H15). ^13^C NMR (100 MHz, CDCl_3_) δ [ppm]: 179.15 (C1), 173.42 (C21), 157.49 (C4), 136.40 (C7), 133.61 (C11), 129.29 (C12), 128.97 (C5), 126.99 (C13), 126.50 (C6), 125.96 (C10), 118.78 (C8), 105.57 (C8), 68.71 (C22), 58.12 (C19), 55.29 (C14), 45.99 (C2), 38.51 (C17), 24.89 (C26), 21.79 (23), 18.55 (C3), 15.30 (C18), 11.72 (C15). FT-IR ν (ATR): 3061, 2973, 2933, 2879, 2838, 2658, 2189, 1741, 1628, 1604, 1505, 1481, 1469, 1455, 1417, 1383, 1356, 1314, 1272, 1263, 1226, 1211, 1194, 1175, 1158, 1117, 1105, 1060, 1031, 998, 965, 942, 925, 907, 897, 880, 855, 818, 808, 794, 771, 762, 750, 711, 683, 652, 621, 572, 532, 523, 507, 477, 420 cm^–1^. UV–Vis (EtOH): λ_max_ = 230, 272, 332 nm. Elemental analysis: Calc (%) for: C_23_H_33_NO_5_ (403.512 g/mol) C (68.46), H (8.24), N (3.47), O (19.83), found: C (67.94), H (8.18), N (3.51), O (19.76).

[L-ThrOiPr][NAP]–L-threonine isopropyl ester naproxenate


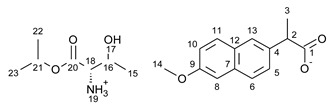


^1^H NMR (400 MHz, CDCl_3_) δ [ppm]: 7.70–7.63 (s+d, 3H, H11, H8, H6), 7.40 (d, J_10,11_ = 6.9 Hz, 1H, H10), 7.14–7.06 (s+d, 2H, H13, H5), 5.06–5.01 (m, 1H, H21), 4.97 (s, 4H, H17, H19), 3.94–3.85 (m, 4H, H14, H16), 3.83–3.75 (m, 1H, H2), 3.25 (d, J_18,17_ = 5.4 Hz, 1H, H18), 1.54 (d, J_3,2_ = 7.2 Hz, 3H, H3), 1.26–1.20 (m, 9H, H23, H22, H15). ^13^C NMR (100 MHz, CDCl_3_) δ [ppm]: 179.58 (C1), 172.49 (C20), 157.56 (C9), 136.03 (C4), 133.65 (C7), 129.29 (C11), 128.93 (C12), 127.08 (C5), 126.41 (C13), 126.01 (C6), 118.90 (C10), 105.56 (C8), 69.31 (C17), 67.90 (C21), 59.66 (C18), 55.31 (C14), 45.86 (C2), 21.73 (C22), 21.70 (C23), 19.88 (C3), 18.45 (C15). FT-IR ν (ATR): 3063, 2977, 2936, 2905, 2080, 1742, 1652, 1630, 1605, 1564, 1541, 1533, 1521, 1516, 1506, 1505, 1487, 1458, 1456, 1445, 1436, 1423, 1385, 1356, 1342, 1290, 1258, 1228, 1213, 1198, 1181, 1161, 1143, 1121, 1104, 1072, 1051, 1031, 1005, 985, 957, 926, 913, 892, 858, 853, 819, 808, 784, 758, 712, 685, 671, 531, 522, 474 cm^–1^. UV–Vis (EtOH): λ_max_ = 230, 272, 332 nm. Elemental analysis: Calc (%) for: C_21_H_29_NO_6_ (391.458 g/mol) C (64.43), H (7.47), N (3.58), O (24.52), found: C (64.39), H (7.39), N (3.43), O (24.18).

[L-MetOiPr][NAP]–L-methionine isopropyl ester naproxenate


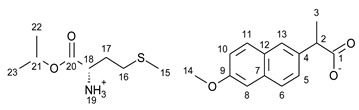


^1^H NMR (400 MHz, CDCl_3_) δ [ppm]: 7.73–7.61 (s+d, 3H, H11, H8, H6), 7.42 (d, J_10,11_ = 6.8 Hz, 1H, H10), 7.15–7.06 (s+d, 2H, H13, H5), 5.55 (s, 3H, H19), 5.09–4.95 (m, 1H, H21), 3.90 (s, 3H, H14), 3.85–3.75 (m, 1H, H2), 3.63–3.56 (m, 1H, H18), 2.53 (d, J_16,17_ = 8.0 Hz, 2H, H16), 2.03 (s, 3H, H15), 2.06–1.94 (m, 1H, H17′), 1.89–1.76 (m, 1H, H17″), 1.54 (d, J_3,2_ = 7.2 Hz, 3H, H3), 1.29–1.17 (2d, 6H, H23, H22). ^13^C NMR (100 MHz, CDCl_3_) δ [ppm]: 179.14 (C1), 174.06 (C20), 157.56 (C9), 136.00 (C4), 133.67 (C7), 129.30 (C11), 128.95 (C12), 127.08 (C5), 126.40 (C13), 126.00 (C16), 118.88 (C10), 105.58 (C8), 68.99 (C21), 55.30 (C14), 52.99 (C18), 45.75 (C2), 33.09 (C17), 30.19 (C16), 21.77 (C22), 21.74 (C23), 18.42 (C3), 15.28 (C15). FT-IR ν (ATR): 3057, 3031, 2981, 2912, 2866, 2177, 2021, 1740, 1682, 1604, 1548, 1505, 1482, 1464, 1450, 1421, 1381, 1353, 1344, 1262, 1228, 1194, 1179, 1160, 1118, 1100, 1070, 1028, 990, 955, 927, 896, 885, 855, 824, 811, 761, 750, 718, 684, 667, 654, 629, 574, 531, 523, 502, 476, 469, 443, 436, 426 cm^–1^. UV–Vis (EtOH): λ_max_ = 230, 272, 332 nm. Elemental analysis: Calc (%) for: C_22_H_31_NO_5_S (421.550 g/mol) C (62.68), H (7.41), N (3.32), O (18.98), S (7.61), found: C (61.95), H (7.36), N (3.30), O (18.61), S (7.51).

[L-IleOiPr][SA]–L-isoleucine isopropyl ester salicylate


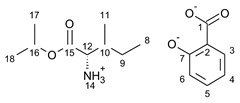


^1^H NMR (400 MHz, CDCl_3_) δ [ppm]: 7.80 (d, J_7,6_ = 7.8 Hz, 1H, H7), 7.30 (t, J_5,4_ = 6.6 Hz, 1H, H5), 6.87 (d, J_4,5_ = 7.2 Hz, 1H, H4), 6.74 (t, J_7,6_ = 7.6 Hz, 1H, H6), 5.02–4.88 (m, 1H, H15), 3.86 (d, J_12,14_ = 3.9 Hz, 1H, H12), 2.04–1.93 (m, 1H, H4), 1.54–1.39 (m, 1H, H9′), 1.38–1.23 (m, 1H, H9″), 1.14 (d, J_16,15_ = 6.3 Hz, 3H, H16), 0.97 (d, J_11,10_ = 6.8 Hz, 3H, H11), 0.83 (t, J_8,9_ = 7.4 Hz, 3H, H8). ^13^C NMR (100 MHz, CDCl_3_) δ [ppm]: 175.47 (C1), 169.03 (C14), 161.66 (C3), 133.68 (C5), 130.69 (C7), 118.14 (C2), 117.43 (C6), 116.76 (C4), 70.52 (C15), 57.29 (C12), 36.93 (C10), 25.75 (C9), 21.60 (C16), 21.46 (C17), 14.57 (C11), 11.57 (C8). FT–IR: ν (ATR): 2966, 2935, 2878, 2638, 2161, 2148, 1733, 1592, 1562, 1541, 1481, 1466, 1450, 1411, 1378, 1358, 1308, 1284, 1251, 1230, 1193, 1160, 1141, 1100, 1054, 1029, 998, 956, 937, 903, 860, 847, 827, 807, 756, 705, 665, 615, 570, 536, 453 cm^–1^. UV–Vis (EtOH): λ_max_ = 208, 232, 300 nm. Elemental analysis: Calc (%) for: C_16_H_24_NO_4_ (310.366 g/mol) C (61.92), H (7.79), N (4.51), O (25.78); found: C (62.13), H (7.85), N (4.48), O (25.52).

[L-ThrOiPr][SA]–L-threonine isopropyl ester salicylate


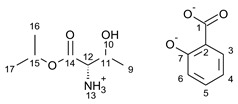


^1^H NMR (400 MHz, CDCl_3_) δ [ppm]: 7.27 (t, J_5,4_ = 7.3 Hz, 1H, H5), 6.84 (d, J_4,5_ = 8.2 Hz, 1H, H4), 6.72 (t, J_6,5_ = 7.7 Hz, 1H, H6), 5.02–4.88 (m, 1H, H14), 4.26–4.15 (m, 1H, H9), 3.79 (d, J_11,12_ = 5.3 Hz, 1H, H11), 1.34 (d, J_8,9_ = 6.5 Hz, 3H, H8), 1.18–1.08 (2t, 6H, H15, H16). ^13^C NMR (100 MHz, CDCl_3_) δ [ppm]: 175.54 (C1), 168.48 (C13), 161.30 (C3), 133.86 (C5), 130.59 (7), 118.50 (C2), 117.22 (C6), 116.81 (C4), 71.32 (9), 66.32 (C14), 59.28 (C11), 21.41 (C15), 21.38 (C16), 20.42 (C8). FT–IR: ν (ATR): 3048, 2981, 2936, 2070, 1735, 1684, 1591, 1560, 1482, 1455, 1377, 1351, 1294, 1250, 1224, 1183, 1141, 1098, 1050, 1030, 953, 913, 896, 860, 808, 755, 704, 665, 566, 535, 494, 458, 418 cm^–1^. UV–Vis (EtOH): λ_max_ = 208, 232, 300 nm. Elemental analysis: Calc (%) for: C_14_H_20_NO_6_ (298.312 g/mol): C (56.37), H (6.76), N (4.70), O (32.18), found: C (56.24), H (6.67), N (4.65), O (32.02).

[L-MetOiPr][SA]–L-methionine isopropyl ester salicylate


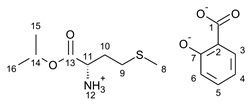


^1^H NMR (400 MHz, CDCl_3_) δ [ppm]: 7.79 (d, J_7,6_ = 7.8 Hz, 1H, H7), 7.32 (t, J_5,4_ = 6.6 Hz, 1H, H5), 6.89 (d, J_4,5_ = 7.3 Hz, 1H, H4), 6.77 (t, J_6,5_ = 7.5 Hz, 1H, H6), 4.16–4.05 (m, 2H, H15), 4.05–3.98 (m, 1H, H12), 2.70–2.53 (m, 2H, H10), 2.25–2.07 (m, 2H, H9), 1.97 (s, 3H, H8), 1.16 (d, J_15,14_ = 6.3 Hz, 3H, H15), 1.12 (d, J_16,14_ = 6.3 Hz, 3H, H16). ^13^C NMR (100 MHz, CDCl_3_) δ [ppm]: 175.54 (C1), 170.15 (C14), 161.61 (C3), 134.03 (C5), 130.55 (C7), 118.39 (C2), 117.06 (C6), 116.97 (C4), 62.65 (C15), 52.07 (C12), 30.18 (C10), 29.49 (C9), 14.97 (C8), 13.86 (C16). FT–IR: ν (ATR): 2981, 2918, 2835, 2756, 2631, 2116, 1733, 1684, 1626, 1593, 1557, 1481, 1448, 1384, 1376, 1349, 1309, 1285, 1248, 1232, 1202, 1182, 1160, 1143, 1103, 1029, 1007, 965, 955, 944, 916, 902, 884, 861, 821, 808, 797, 753, 704, 665, 567, 534, 496, 476, 455, 431 cm^–1^. UV–Vis (EtOH): λ_max_ = 210, 232, 300 nm. Elemental analysis: Calc (%) for: C_15_H_22_NO_5_S (328.404 g/mol): C (54.86); H (6.75); N (4.26); O (24.36); S (9.76), found: C (54.96); H (6.79); N (4.24); O (24.10); S (9.59).

### 3.2. Antimicrobial Susceptibility Test by Disc Diffusion Technique

Antibacterial activity was evaluated by the EUCAST diffusion disc method [75] by measuring the inhibition zone against *Escherichia coli* (gram-negative bacteria), *Micrococcus luteus*, and *Staphylococcus epidermidis* (as gram-positive bacteria). The bacterial cultures of E. coli (ACCT 29425), *M. luteus* (ATCC 7468), and *S. epidermidis* (ACCT 12228) were obtained from DSMZ (Braunschweig, Germany).

Preparation of the liquid nutrient and the agar medium for bacterial growth was prepared according to the producer’s recommendations. The sterilization process was carried out for 15 min at a temperature of 120 °C. The enriched broth was used for the growth of *E. coli*, and the lysogenic broth (LB Broth) for the growth of *M. luteus* bacteria. For the cultivation of *S. epidermidis*, bacteria with high nutritional requirements, cerebral–cardiac broth was used for the cultivation. As the agar medium, the Nutrient LAB–AGAR™ was used for the isolation of *M. luteus*, while the Brain Heart Infusion LAB–AGAR™ was used for the isolation of *S. epidermidis*. For the determination of the number of *Escherichia coli* bacterial cells, the TTC lactose agar medium with tergitol^®^7 was used due to the presence of a selective factor in the form of Tergitol-7 medium, which inhibits the growth of gram-positive bacteria and limits the growth of *Proteus* spp. In addition, the so-called indicator system in the form of bromothymol blue and TTC allows for easier differentiation of *E. coli.* The prepared agar medium was poured into sterile plastic Petri plates. After that, the agar was allowed to solidify, followed by transfer to an incubator set at 37 °C for 48 h to evaporate the water from the agar surface. Filter paper discs for antibiotic resistance testing of 5 mm diameter were saturated with the proper solution of each compound and allowed to dry for 10 min in a laminar airflow cabinet. After that, discs were manually placed by using sterile forceps on the indicated agar mediums, which were previously inoculated evenly over the entire Petri plate with a specific culture of the bacterium (1.5 × 10^6^ CFU/cm^3^, CFU–colony forming units). The concentration of studied compounds were: 1000, 800, 600, 400, 200, 100, 50, 25 mg cm^–3^ in absolute ethanol. The plates were incubated for 24 h at 37 °C. The inhibition zone, defined as the circular area around the disc in which the bacterial colonies do not grow, was evaluated by measuring the diameter of the clear zone (in mm) to an accuracy of 0.1 mm. Each prepared sample was measured in three replicates. The obtained results were expressed as a mean of inhibition zones with standard deviation.

## 4. Conclusions

Sixteen salts of amino acid isopropyl esters based on acids from the non-steroidal anti-inflammatory group were synthetized, of which nine have not yet been described in the literature. All of them were classified as amino acid ionic liquids with pharmaceutical activity. The determined physicochemical properties revealed the potential usage of the obtained compounds for application on the skin. The antimicrobial activity of the obtained salts against *Escherichia coli*, *Staphylococcus epidermidis*, and *M. Luteus* was evaluated. Studied bacteria indicated various susceptibilities against L-amino acid isopropyl ester salts, more dependent on the structure of the counterion of the active substance. Although the permeation study through the skin was not part of this study, our previous work demonstrated that L-amino acid isopropyl ester-based conjugations with ibuprofen improve the skin permeation rates [4,6]. Therefore, further investigation should be considered to evaluate the skin permeation rate of naproxen, ketoprofen and salicylic acid salts and the impact of the L-amino acid isopropyl ester moiety structure on the impact of L-amino acids and their permeability. The obtained conjugations of L-amino acid esters may be potentially used in topical and transdermal administration of both amino acids and active substances in sufficient amounts to cause therapeutic effects. In addition, due to its antibacterial activity, amino acid isopropyl ester salts could be an alternative tool to treat a delayed healing wound and used in topical drug formulations.

## Figures and Tables

**Table 1:** Melting points, thermal stability, and specific and molar rotation for NSAIDs and their amino acid isopropyl esters.

Compound	T_DSConset_ (°C)	T_DSCmax_ (°C)	T_C_ (°C)	T_TGonset_ (°C)	T_DTGmax_ (°C)	${\left[\alpha \right]}_{\lambda }^{20}$	${\left[M\right]}_{\lambda }^{20}$
IBU	76.23	78.58	-	186.2	218.8	-	-
[L-ValOiPr][IBU]	65.84	91.15	66.33	90.2 [50]	215.5 [50]	+11.852 [50]	+43.313 [50]
[L-IleOiPr][IBU]	73.68/ 79.98	77.24/ 82.56	52.67	105.2 [50]	221.9 [50]	+15.779 [50]	+59.887 [50]
[L-ThrOiPr][IBU]	44.62	53.17	-	129.6 [50]	209.9 [50]	−1.538 [50]	−5.654 [50]
[L-MetOiPr][IBU]	60.42	65.09	47.04	146.2 [50]	209.9 [50]	+3.137 [50]	+12.472 [50]
KETO	95.14	97.03	-	241.4	304.9	-	-
[L-ValOiPr][KETO]	69.84	75.65	-	60.7 [47]	307.6 [47]	+8.083 [47]	+33.424 [47]
[L-IleOiPr][KETO]	61.70/ 73.38	68.73/ 81.36	-	116.6	319.6	+9.053	+38.407
[L-ThrOiPr][KETO]	-	-	-	82.4	295.1	−9.742	−40.475
[L-MetOiPr][KETO]	49.62	61.67	-	98.0	306.5	+9.182	+40.912
NAP	155.98	158.41	98.60	224.5	284.0	+50.988	+117.405
[L-ValOiPr][NAP]	107.23/ 119.16	115.32/ 128.19	81.52	112.2 [48]	275.8 [48]	+30.740 [48]	+119.729 [48]
[L-IleOiPr][NAP]	74.50/ 86.79	82.58/ 92.13	56.74	127.0	287.4	+34.343	+138.578
[L-ThrOiPr][NAP]	87.16	91.97	-	145.8	295.3	+26.608	+104.159
[L-MetOiPr][NAP]	71.00	83.79	55.66	149.6	293.2	+26.693	+112.524
SA	158.05	160.04	107.83	146.2	198.8	-	-
[L-ValOiPr][SA]	100.13	102.33	83.01	124.6 [49]	197.6 [49]	+12.457 [49]	+37.040 [49]
[L-IleOiPr][SA]	86.10	88.50	67.26	144.2	202.4	+18.939	+58.780
[L-ThrOiPr][SA]	-	-	-	127.2	191.4	−4.105	−12.245
[L-MetOiPr][SA]	85.39	89.09	51.62	161.1	205.0	+10.575	+34.729

T_DSConset_: the peak onset temperature in DSC; T_DSCmax_: the peak maximum temperature in DSC; T_C_: crystallization maximum peak temperature; T_TGonset_: the onset of the thermal degradation; T_DTGmax_: maximum decomposition temperature; ${\left[\alpha \right]}_{\lambda }^{20}$: specific rotation; ${\left[M\right]}_{\lambda }^{20}$: molar rotation.

**Table 2:** Solubility of NSAIDs and their amino acid isopropyl esters salts in organic solvents.

Compound	Ethanol (59.1)	DMSO (45.1)	Dichloromethane (40.7)	Chloroform (39.1)	Ethyl Acetate (38.1)	Diethyl Ether (34.5)	Toluene (33.9)	Hexane (31.0)
IBU	○	○	○	○	○	○	○	●
[ValOiPr][IBU]	○ [45]	○ [45]	○ [45]	○ [45]	◐ [45]	◐ [45]	○ [45]	● [45]
[IleOiPr][IBU]	○ [50]	○ [50]	○ [50]	○ [50]	○ [50]	◐ [50]	○ [50]	● [50]
[ThrOiPr][IBU]	○ [50]	○ [50]	○ [50]	○ [50]	◐ [50]	◐ [50]	◐ [50]	● [50]
[MetOiPr][IBU]	○ [50]	○ [50]	○ [50]	○ [50]	○ [50]	○ [50]	○ [50]	● [50]
KETO	○	○	○	○	◐	◐	●	●
[ValOiPr][KETO]	○ [47]	○ [47]	○ [47]	◐ [47]	● [47]	○ [47]	○ [47]	● [47]
[IleOiPr][KETO]	○	○	○	○	●	●	○	●
[ThrOiPr][KETO]	○	○	◐	○	●	●	○	●
[MetOiPr][KETO]	◐	○	○	○	●	●	○	●
NAP	○	○	○	○	◐	◐	●	●
[ValOiPr][NAP]	◐ [48]	◌ [48]	◌ [48]	◌ [48]	● [48]	◐ [48]	● [48]	● [48]
[IleOiPr][NAP]	○	○	○	○	●	●	◐	●
[ThrOiPr][NAP]	○	○	○	○	●	●	◐	●
[MetOiPr][NAP]	○	○	○	○	●	●	○	●
SA	○	○	○	◐	○	○	◐	●
[ValOiPr][SA]	○ [49]	○ [49]	○ [49]	○ [49]	◐ [49]	◐ [49]	○ [49]	● [49]
[IleOiPr][SA]	○	○	○	○	○	○	○	●
[ThrOiPr][SA]	○	○	○	○	○	●	●	●
[MetOiPr][SA]	○	○	○	○	○	○	○	●

Solvents were ranked with decreasing value of empirical solvent polarity parameters, E_T_(30) (“○”: soluble >100 mg cm^−3^; “◐”: partially soluble 33–100 mg cm^−3^; “●”: insoluble <33 mg cm^−3^) at the temperature 25 °C by modified Vogel’s method [56].

**Table 3:** Solubility of acids from NSAIDs group and their L-amino acid isopropyl ester salts in water and phosphate buffers at 25 °C.

Compound	Solubility in Water		Solubility in Phosphate Buffer			
			pH = 5.4		pH = 7.4	
	g dm^−3^	g_AS_ dm^−3^	g dm^−3^	g_AS_ dm^−3^	g dm^−3^	g_AS_ dm^−3^
IBU	0.076 ± 0.001 [45]	0.076 ± 0.001 [45]	0.082 ± 0.001 [45]	0.082 ± 0.001 [45]	0.432 ± 0.001 [45]	0.432 ± 0.001 [45]
[L-ValOiPr][IBU]	3.468 ± 0.007 [45]	1.957 ± 0.007 [45]	2.351 ± 0.027 [45]	1.326 ± 0.027 [45]	4.998 ± 0.018 [45]	2.821 ± 0.018 [45]
[L-IleOiPr][IBU]	2.729 ± 0.180 [50]	1.483 ± 0.098 [50]	1.161 ± 0.002	0.631 ± 0.002	4.038 ± 0.021	2.195 ± 0.021
[L-ThrOiPr][IBU]	5.005 ± 0.007 [50]	2.809 ± 0.004 [50]	4.931 ± 0.013	2.768 ± 0.013	8.839 ± 0.005	4.962 ± 0.005
[L-MetOiPr][IBU]	1.191 ± 0.056 [50]	0.618 ± 0.029 [50]	0.932 ± 0.011	0.483 ± 0.011	3.376 ± 0.001	1.752 ± 0.001
KETO	0.013 ± 0.001	0.013 ± 0.001	0.024 ± 0.001	0.024 ± 0.001	0.079 ± 0.003	0.079 ± 0.003
[L-ValOiPr][KETO]	0.445 ± 0.014	0.274 ± 0.014	0.162 ± 0.054	0.100 ± 0.054	1.447 ± 0.011	0.890 ± 0.011
[L-IleOiPr][KETO]	0.577 ± 0.006	0.343 ± 0.006	0.344 ± 0.032	0.205 ± 0.032	1.173 ± 0.024	0.698 ± 0.024
[L-ThrOiPr][KETO]	1.012 ± 0.001	0.619 ± 0.001	0.823 ± 0.027	0.504 ± 0.027	2.566 ± 0.036	1.570 ± 0.036
[L-MetOiPr][KETO]	0.289 ± 0.004	0.165 ± 0.004	0.171 ± 0.058	0.098 ± 0.058	1.086 ± 0.007	0.622 ± 0.007
NAP	0.147 ± 0.001	0.147 ± 0.001	0.221 ± 0.015	0.221 ± 0.015	1.379 ± 0.021	1.379 ± 0.021
[L-ValOiPr][NAP]	3.988 ± 0.020	2.358 ± 0.020	2.323 ± 0.033	1.320 ± 0.033	5.484 ± 0.012	3.241 ± 0.012
[L-IleOiPr][NAP]	5.048 ± 0.027	2.881 ± 0.027	3.206 ± 0.017	1.829 ± 0.017	7.736 ± 0.030	4.415 ± 0.030
[L-ThrOiPr][NAP]	6.457 ± 0.019	3.798 ± 0.019	4.381 ± 0.008	2.585 ± 0.008	8.711 ± 0.029	5.137 ± 0.029
[L-MetOiPr][NAP]	3.011 ± 0.038	1.645 ± 0.038	1.509 ± 0.015	0.824 ± 0.015	4.404 ± 0.033	2.405 ± 0.033
SA	3.795 ± 0.004	3.795 ± 0.004	3.862 ± 0.005	3.862 ± 0.005	5.757 ± 0.024	5.757 ± 0.024
[L-ValOiPr][SA]	16.708 ± 2.044	7.761 ± 2.044	11.322 ± 0.014	5.259 ± 0.014	18.733 ± 0.027	8.702 ± 0.027
[L-IleOiPr][SA]	26.500 ± 0.422	11.793 ± 0.422	19.281 ± 0.015	8.581 ± 0.015	29.548 ± 0.041	13.149 ± 0.041
[L-ThrOiPr][SA]	38.714 ± 0.241	17.925 ± 0.241	30.189 ± 0.018	13.978 ± 0.018	40.757 ± 0.125	18.777 ± 0.125
[L-MetOiPr][SA]	16.046 ± 0.533	6.749 ± 0.533	10.550 ± 0.007	4.437 ± 0.007	18.036 ± 0.046	7.586 ± 0.046

AS, active substance (unmodified acid).

**Table 4:** The n-octanol-water partition coefficient (logP) of acids from NSAID groups and their L-amino acid isopropyl ester salts.

Compound	LogP			
	[IBU]	[KETO]	[NAP]	[SA]
Unmodified acid	3.208 ± 0.002 [50]	1.577 ± 0.010	2.119 ± 0.021	1.321 ± 0.018
[L-ValOiPr]	1.154 ± 0.004 [45]	0.570 ± 0.006	1.254 ± 0.016	1.610 ± 0.057
[L-IleOiPr]	1.652 ± 0.008 [50]	0.897 ± 0.004	1.188 ± 0.033	1.601 ± 0.034
[L-ThrOiPr]	0.998 ± 0.001 [50]	0.516 ± 0.001	0.089 ± 0.005	1.555 ± 0.021
[L-MetOiPr]	1.509 ± 0.001 [50]	1.227 ± 0.015	1.372 ± 0.005	1.784 ± 0.035

**Table 5:** The formation constants log(K_S_) of L-amino acid isopropyl ester salts.

Compound	pK_B_ of the Base	Log(K_S_)			
		[IBU]	[KETO]	[NAP]	[SA]
[L-ValOiPr]	10.210	5.56	6.10	5.89	7.03
[L-IleOiPr]	10.430	5.78	6.32	6.11	7.25
[L-ThrOiPr]	11.118	6.47	6.80	6.80	7.94
[L-MetOiPr]	10.176	5.53	5.86	6.07	7.00

**Table 6:** Antimicrobial activity against *Escherichia coli* of acids from NSAID groups and their L-amino acid isopropyl ester salts, Inhibition diameter (mm).

Compound	Concentration of Solution Transferred to Disc [mg cm^−3^]						
	1000	600	400	200	100	50	25
IBU	-	-	-	-	-	-	-
[L-ValOiPr][IBU]	7.1 ± 0.3	6.8 ± 0.5	6.3 ± 0.2	6.2 ± 0.1	-	-	-
[L-IleOiPr][IBU]	7.8 ± 0.3	7.1 ± 0.2	6.4 ± 0.3	6.3 ± 0.1	-	-	-
[L-ThrOiPr][IBU]	-	-	-	-	-	-	-
[L-MetOiPr][IBU]	8.1 ± 0.1	7.6 ± 0.2	7.1 ± 0.3	6.5 ± 0.2	6.2 ± 0.1	6.1 ± 0.1	-
KETO	-	-	-	-	-	-	-
[L-ValOiPr][KETO]	8.3 ± 0.4	7.6 ± 0.3	7.0 ± 0.2	6.7 ± 0.3	-	-	-
[L-IleOiPr][KETO]	-	-	-	-	-	-	-
[L-ThrOiPr][KETO]	-	-	-	-	-	-	-
[L-MetOiPr][KETO]	12.9 ± 0.4	10.6 ± 0.7	8.9 ± 0.5	8.0 ± 0.6	7.3 ± 0.5	-	-
NAP	-	-	-	-	-	-	-
[L-ValOiPr][NAP]	-	-	-	-	-	-	-
[L-IleOiPr][NAP]	-	-	-	-	-	-	-
[L-ThrOiPr][NAP]	-	-	-	-	-	-	-
[L-MetOiPr][NAP]	7.7 ± 0.2	7.3 ± 0.2	7.1 ± 0.1	6.4 ± 0.3	-	-	-
SA	13.1 ± 1.2	11.0 ± 0.8	6.4 ± 0.1	6.1 ± 0.1	-	-	-
[L-ValOiPr][SA]	16.5 ± 1.0	12.6 ± 0.3	8.9 ± 0.4	7.5 ± 0.1	6.3 ± 0.2	6.2 ± 0.1	-
[L-IleOiPr][SA]	14.8 ± 0.7	13.7 ± 0.7	7.3 ± 0.3	6.2 ± 0.1	-	-	-
[L-ThrOiPr][SA]	13.2 ± 0.8	9.3 ± 0.4	7.3 ± 0.2	-	-	-	-
[L-MetOiPr][SA]	21.8 ± 0.7	18.8 ± 0.4	16.4 ± 0.3	14.4 ± 0.8	7.8 ± 0.2	-	-

(-), no growth of the organism.

**Table 7:** Antimicrobial activity against *Staphylococcus epidermidis* of acids from NSAIDs and their L-amino acid isopropyl esters salts, Inhibition diameter (mm).

Compound	Concentration of Solution Transferred to Disc [mg cm^−3^]							
	1000	800	600	400	200	100	50	25
IBU	20.6 ± 0.2	18.6 ± 0.2	17.6 ± 0.2	16.4 ± 0.2	15.3 ± 0.3	14.9 ± 0.3	11.5 ± 0.3	10.8 ± 0.3
[L-ValOiPr][IBU]	18.9 ± 0.3	18.4 ± 0.2	17.7 ± 0.3	16.3 ± 0.3	13.2 ± 0.2	12.8 ± 0.2	10.4 ± 0.1	7.3 ± 0.2
[L-IleOiPr][IBU]	19.3 ± 0.1	18.0 ± 0.1	17.5 ± 0.1	17.0 ± 0.2	15.4 ± 0.4	14.4 ± 0.2	9.5 ± 0.4	7.5 ± 0.2
[L-ThrOiPr][IBU]	23.2 ± 0.1	19.8 ± 1.4	18.3 ± 0.2	17.5 ± 0.2	16.9 ± 0.2	15.4 ± 0.1	10.5 ± 0.2	7.5 ± 0.2
[L-MetOiPr][IBU]	17.7 ± 0.3	17.0 ± 0.2	15.4 ± 0.2	14.7 ± 0.2	13.6 ± 0.2	8.3 ± 0.1	-	-
KETO	-	-	-	-	-	-	-	-
[L-ValOiPr][KETO]	6.9 ± 0.2	6.3 ± 0.2	6.3 ± 0.1	6.2 ± 0.1	-	-	-	-
[L-IleOiPr][KETO]	10.4 ± 0.2	8.3 ± 0.1	7.3 ± 0.2	6.2 ± 0.1	-	-	-	-
[L-ThrOiPr][KETO]	-	-	-	-	-	-	-	-
[L-MetOiPr][KETO]	7.4 ± 0.2	-	-	-	-	-	-	-
NAP	-	-	-	-	-	-	-	-
[L-ValOiPr][NAP]	7.4 ± 0.1	6.6 ± 0.3	-	-	-	-	-	-
[L-IleOiPr][NAP]	13.5 ± 0.3	12.4 ± 0.2	12.0 ± 0.3	-	-	-	-	-
[L-ThrOiPr][NAP]	-	-	-	-	-	-	-	-
[L-MetOiPr][NAP]	7.5 ± 0.3	-	-	-	-	-	-	-
SA	12.6 ± 0.7	8.6 ± 0.3	7.0 ± 0.2	6.2 ± 0.1	-	-	-	-
[L-ValOiPr][SA]	10.7 ± 0.3	8.4 ± 0.3	6.7 ± 0.3	-	-	-	-	-
[L-IleOiPr][SA]	16.6 ± 0.5	10.5 ± 0.3	7.3 ± 0.4	-	-	-	-	-
[L-ThrOiPr][SA]	-	-	-	-	-	-	-	-
[L-MetOiPr][SA]	10.7 ± 0.6	9.2 ± 0.5	7.5 ± 0.2	7.1 ± 0.1	6.6 ± 0.2	-	-	-

(-), no growth of the organism.

**Table 8:** Antimicrobial activity against *Micrococcus Luteus* of acids from NSAIDs and their L-amino acid isopropyl ester salts, Inhibition diameter (mm).

Compound	Concentration of Solution Transferred to Disc [mg cm^−3^]							
	1000	800	600	400	200	100	50	25
IBU	13.4 ± 0.1	12.7 ± 0.2	11.5 ± 0.2	10.6 ± 0.1	10.2 ± 0.1	9.7± 0.2	9.4 ± 0.2	8.6 ± 0.3
[L-ValOiPr][IBU]	13.2 ± 0.2	12.6 ± 0.3	11.7 ± 0.2	11.2 ± 0.2	10.5 ± 0.2	9.6 ± 0.1	9.1 ± 0.2	8.4 ± 0.3
[L-IleOiPr][IBU]	12.3 ± 0.2	11.6 ± 0.1	11.2 ± 0.1	10.7 ± 0.1	9.4 ± 0.2	8.6 ± 0.1	7.6 ± 0.1	7.2 ± 0.2
[L-ThrOiPr][IBU]	19.3 ± 0.2	18.2 ± 0.2	17.5 ± 0.1	17.0 ± 0.2	16.3 ± 0.1	15.5 ± 0.2	10.4 ± 0.2	7.3 ± 0.2
[L-MetOiPr][IBU]	14.6 ± 0.3	14.3 ± 0.1	14.1 ± 0.1	12.6 ± 0.1	10.4 ± 0.1	8.5 ± 0.2	7.3 ± 0.2	-
KETO	17.3 ± 0.2	14.2 ± 0.1	13.3 ± 0.2	12.5 ± 0.2	11.1 ± 0.4	10.2 ± 0.2	9.6 ± 0.2	8.3 ± 0.3
[L-ValOiPr][KETO]	15.1 ± 0.2	14.5 ± 0.2	11.7 ± 0.1	10.3 ± 0.2	9.1 ± 0.3	8.5 ± 0.3	7.4 ± 0.1	6.3 ± 0.2
[L-IleOiPr][KETO]	9.4 ± 0.2	8.4 ± 0.3	8.3 ± 0.2	7.3 ± 0.1	7.1 ± 0.2	6.6 ± 0.3	6.3 ± 0.2	-
[L-ThrOiPr][KETO]	17.4 ± 0.1	15.4 ± 0.2	14.3 ± 0.2	9.6 ± 0.1	7.4 ± 0.3	-	-	-
[L-MetOiPr][KETO]	14.3 ± 0.2	12.4 ± 0.2	9.6 ± 0.2	9.2 ± 0.1	8.5 ± 0.3	8.2 ± 0.2	7.2 ± 0.2	-
NAP	8.7 ± 0.1	8.5 ± 0.2	7.6 ± 0.2	7.3 ± 0.2	6.8 ± 0.1	6.7 ± 0.1	6.4 ± 0.1	6.2 ± 0.1
[L-ValOiPr][NAP]	8.6 ± 0.2	8.2 ± 0.2	7.4 ± 0.2	7.3 ± 0.1	7.2 ± 0.1	6.4 ± 0.1	6.3 ± 0.1	6.2 ± 0.1
[L-IleOiPr][NAP]	9.5 ± 0.1	8.7 ± 0.1	8.1 ± 0.6	7.5 ± 0.2	7.3 ± 0.1	7.0 ± 0.1	6.7 ± 0.1	6.4 ± 0.1
[L-ThrOiPr][NAP]	10.8 ± 0.5	9.6 ± 0.1	8.8 ± 0.2	8.5 ± 0.1	7.6 ± 0.1	6.9 ± 0.1	6.6 ± 0.1	6.2 ± 0.1
[L-MetOiPr][NAP]	7.8 ± 0.2	7.5 ± 0.1	7.1 ± 0.2	6.4 ± 0.1	-	-	-	-
SA	13.1 ± 1.9	9.4 ± 0.8	8.7 ± 0.8	7.4 ± 0.4	-	-	-	-
[L-ValOiPr][SA]	17.4 ± 1.4	15.9 ± 1.2	8.6 ± 0.3	-	-	-	-	-
[L-IleOiPr][SA]	23.5 ± 2.2	12.4 ± 0.4	10.8 ± 0.5	-	-	-	-	-
[L-ThrOiPr][SA]	12.7 ± 1.0	10.2 ± 0.7	8.7 ± 0.5	8.1 ± 0.1	7.6 ± 0.2	7.0 ± 0.1	6.6 ± 0.2	6.3 ± 0.1
[L-MetOiPr][SA]	11.1 ± 0.9	10.5 ± 1.0	8.1 ± 0.9	7.3 ± 0.4	6.3 ± 0.1	6.1 ± 0.1	-	-

(-), no growth of the organism.

## Data Availability

The data presented in this study are available on request from the corresponding author.

## Supplementary Materials

The following supporting information can be downloaded at: https://www.mdpi.com/article/10.3390/ijms232213863/s1.
