# Supplementary material for: Isopropyl Amino Acid Esters Ionic Liquids as Vehicles for Non-Steroidal Anti-Inflammatory Drugs in Potential Topical Drug Delivery Systems with Antimicrobial Activity
Source: Int J Mol Sci. 2022 Nov 10;23(22):13863. doi: 10.3390/ijms232213863 (PMC9693575; doi:10.3390/ijms232213863)
Supplement: Supplementary file 1 [file ijms-23-13863-s001.zip › ijms-2001903-supplementary.pdf]

International Journal of *Molecular Sciences*

MDPI

Supplementary Materials

Isopropyl Amino Acid Esters Ionic Liquids as Vehicles for Non-Steroidal Anti-Inflammatory Drugs in Potential Topical Drug Delivery Systems with Antimicrobial Activity

Joanna Klebeko [1,*], Oliver Krüger [2], Mateusz Dubicki [3], Paula Ossowicz-Rupniewska [1] and Ewa Janus [1]

[1] Department of Chemical Organic Technology and Polymeric Materials, Faculty of Chemical Technology and Engineering, West Pomeranian University of Technology in Szczecin, Piastów Ave. 42, 71065 Szczecin, Poland
[2] Department II Mathematics, Physics and Chemistry, Berliner Hochschule für Technik, Luxemburger Straße, 13353 Berlin, Germany
[3] Department of Inorganic Chemical Technology and Environment Engineering, Faculty of Chemical Technology and Engineering, West Pomeranian University of Technology in Szczecin, Piastów Ave. 42, 71065 Szczecin, Poland
* Correspondence: joanna.klebeko@gmail.com; Tel.: +48-449-48-01

Number of pages: 79
Number of Figures: 80
Number of Tables: 3

Table of Contents

Materials and Methods.......................................3
The NMR spectra of [AAOiPr][IBU] 7
The NMR spectra of [AAOiPr][KETO] 9
The NMR spectra of [AAOiPr][NAP] 17
The NMR spectra of [AAOiPr][SA] 25
The ATR-FTIR spectra of [AAOiPr][KETO].......................................36
The ATR-FTIR spectra of [AAOiPr][NAP].......................................38
The ATR-FTIR spectra of [AAOiPr][SA].......................................40
The UV–Vis spectra of [AAOiPr][KETO] 42
The UV–Vis spectra of [AAOiPr][NAP] 44
The UV–Vis spectra of [AAOiPr][SA] 46
X-ray diffraction (XRD) patterns of [AAOiPr][KETO] 52
X-ray diffraction (XRD) patterns of [AAOiPr][NAP].......................................53
X-ray diffraction (XRD) patterns of [AAOiPr][SA] 55
The TG curves of [AAOiPr][KETO].......................................57
The TG curves of [AAOiPr][NAP].......................................60
The TG curves of [AAOiPr][SA] 63
The DSC curves of [AAOiPr][IBU] 66
The DSC curves of [AAOiPr][KETO] 70
The DSC curves of [AAOiPr][NAP] 74
The DSC curves of [AAOiPr][SA].......................................78

Materials and Methods

Materials

All reagents were purchased from commercial suppliers and used without further purification. (*R,S*)-Ibuprofen and S(+)-Naproxen of purity ≥98% were provided by AmBeed (Arlington Hts, USA). (*R,S*)-Ketoprofen (≥98%) was provided by Biosynth Carbosynth (Staad, Switzerland). Salicylic acid, trimethylsilyl chloride (TMSCl), n-octanol of purity ≥99%, and acetonitrile (≥99.9%) for HPLC gradient grade were purchased from Sigma-Aldrich (Steinheim am Albuch, Germany). L-valine, L-isoleucine, L-threonine, and L-methionine (≥98%) were purchased from FluoroChem (Derbyshire, UK). Disodium hydrogen phosphate anhydrous (≥99%) (Na_2HPO_4) and potassium dihydrogen phosphate dihydrogen (≥99.5%) were provided by Fisher Bioreagents (Pittsburgh, PA, USA) and sodium dihydrogen phosphate dihydrate (98%) ($NaH_2PO_4·2H_2O$) were purchased from Acros Organics (Geel, Belgium). Ammonium hydroxide solution 25% ($NH_3·H_2O$) of analytical grade was provided by StanLab (Lublin, Poland). Ethanol (EtOH), propan-2-ol (iPrOH), acetic acid, potassium chloride, sodium chloride, sodium hydroxide, sodium sulfate anhydrous, orthophosphoric acid (98%), hydrochloride acid (35-38%), dimethyl sulfoxide, chloroform, ethyl acetate, diethyl ether, toluene, and n-hexane were high purity and provided by Chempur (Gliwice, Poland). Deuterated chloroform ($CDCl_3$) (99.8%) (+0.03% TMSCl) was purchased from Eurisotop (Cheshire, England).

The bacterial culture of *E.coli* (ATCC 29425), *M. luteus* (ATCC 7468), and *S. epidermidis* (ATCC 12228) were provided by DSMZ (Leibniz-Institut, Deutsche Sammlung von Microorganismen und Zellkulturen GmbH, Braunschweig, Germany). Brain–heart-infusion broth and brain–heart-infusion LabAgar were provided by Carl Roth (Karlsruhe, Germany). *Enrichment broth*, Lactose TTC Agar with Tergitol®7 and Tryptic Soy Broth were purchased from MerckMillipore (Darmstadt, Germany).

Methods

Nuclear magnetic spectroscopy (NMR)

The NMR spectra were recorded in $CDCl_3$ on BRUKER DPX-400 Avance III HD spectrometer (Billerica, MA, USA) operating at 400.13 MHz (^1H) and 100.62 MHz (^{13}C). Tetramethylsilane (TMS) was used as the internal standard. Chemical shifts are given in δ (ppm), and coupling constants *J* are given in Hz.

Total reflectance–Fourier transform infrared spectroscopy (ATR-FTIR)

The ATR-FTIR spectra were registered on a Thermo Fischer Scientific Nicolet 380 (Waltham, MA, USA) spectrometer equipped with an ATR diamond plate in transmission mode. The data were recorded in the range of 4000–400 cm^{-1} at a resolution of 4 cm^{-1}.

UV–Vis spectroscopy (UV–Vis)

UV–Vis spectra were recorded with a Genesys 50 UV–Visible Spectrophotometer from Thermofisher Scientific™ (Waltham, MA, USA). The solutions were prepared in absolute ethanol of concentration range 10^{-5}–10^{-6} M. The measurements were performed in a 10 mm quartz cell in the wavelength range of 190–400 nm with an accuracy of ±1 nm.

Elemental analysis

The elemental composition CHNS/O was determined using a Thermo Scientific™ FLASH 2000 CHNS/O Analyzer (Waltham, MA, USA). The individual elements were detected by a thermal conductivity detector (TCD). The reactor temperature was maintained at 950°C (CNHS analysis) and 1060 °C (O analysis. The samples were prepared in silver crucibles for oxygen determination and tin crucibles for analysis in CHNS mode. All samples were weighed with an accuracy of ±0.000001 g. For device calibration, 2,5-(Bis(5-tert-

butyl-2-benzo-oxazol-2-yl)thiophene (BBOT), sulphanilamide, L-cysteine, and L-methionine (CHNS-mode) acetanilide and benzoic acid (O-mode) were used as standards.

X-ray diffraction (XRD)

The crystallinity of the synthesized compounds was verified by XRD analysis recorded on an AERID PANalytical X-ray diffractometer with Cu-Kα radiation.

Thermogravimetric analysis (TG)

Thermogravimetric analysis was carried out using Netzsch Proteus Thermal Analysis TG 209 F1 Libra apparatus (Selb, Germany). Samples of 5–8 mg were loaded in Al_2O_3 crucibles and heated from 25°C to 1000°C at a heating rate of 10°C min^{-1}. The measurement was performed in an air atmosphere (25 cm^3min^{-1}) with nitrogen flow (10 cm^3min^{-1}) as the purge gas.

Differential scanning calorimetry (DSC)

Phase transformation temperatures were determined on a PerkinElmer DSC-7 (Rotgau, Germany). The samples of 3–5 mg were loaded on the aluminum pan with a crimped lid. Measurements were carried out in a nitrogen atmosphere (20 cm3 min-1). The rate of heating/cooling was 5°C min-1. The sample was first heated from 20°C to 120°C and maintained for 30 min inside the furnace, then cooled to 0°C to condition the sample and the crucible. After that, heating was conducted to the specified temperature, followed by cooling. Unless otherwise stated, two complete cycles of heating and cooling were performed. The specified maximum temperature was individual for each compound, determined from TG analysis, and it was at least 10°C lower than the onset decomposition temperature. Temperatures reported from DSC data are curve peaks, the melting points being endothermic upon heating and crystallization point being exothermic upon cooling. Lead, indium, tin, and zinc were used as standards to calibrate the temperature. Indium was used for enthalpy measurement calibration.

Specific rotation

The specific rotation $[\alpha]_D^{20}$ was measured on an AUTOPOL IV Polarimeter (Rudolph Research Analytical, Hackettstown, NJ, USA) with a 589 nm wavelength for concentrations of compounds of 0.01g cm^{-3} at 20.0 ± 0.1°C. The solutions were prepared in absolute ethanol as the solvent. The angular rotation was tested at an accuracy of 0.001°.

SEM

The morphology measurements were performed using scanning electron microscopy, carried out using a Carl Zeiss Microscope GmbH Model DSM 950 (Oberkochen, Germany). Prior to the measurements, the samples were applied on electrically conductive carbon adhesive discs, which were previously pressed onto aluminum specimen stubs. The samples were then sputter-coated with a thin layer of platinum through target ionization of argon using a Blazers SCD 040 sputter coater. The sputter coating of specimens was conducted at a 15 mA current in an argon atmosphere at 0.05 mbar.

Determination of formation constant pKs

The determination for L-amino acid isopropyl esters and ibuprofen, naproxen, ketoprofen and salicylic acid was performed by potentiometric acid–base titration using a pH meter CP-505 (Elmetron, Zabrze, Poland) equipped with an electrode EPS-1 (Elmetron, Zabrze, Poland). For the measurement of pK_A acids, 0.1 M NaOH was used. In order to assess pK_B, in the first stage, a solution of 0.60 mM of the proper L-amino acid isopropyl ester hydrochloride, HCl aqueous solution, was added in an amount that allowed reaching pH=3.5. After that, an acid-base titration was made out by adding dropwise 5 mM

NaOH solution. Finally, the pK_S values from obtained salts were calculated from the equation:

$$\mathrm{pK}_S = \mathrm{pK}_A + \mathrm{pK}_B - \mathrm{pK}_W$$

The measurements were replicated in five independent measurements and presented as a mean value.

Partition coefficient

The n-octanol/water partition coefficient was determined for acids from the NSAIDs group and their amino acid isopropyl ester salts using the shake flask method. The sample of 10 mg weighted with an accuracy of 0.01 mg was added to 5 cm^3 of n-octanol saturated with water and 5 cm^3 of water saturated with n-octanol. The resulting mixture was vigorously agitated at 25.00 ± 0.05 °C for 3 hours then centrifugated at 7500 rpm at 25 °C for 15 min. After the separation of phases, the aqueous layer was decanted and analyzed by high-performance liquid chromatography (HPLC) to determine the concentration of the compound. The HPLC analyses were performed by using a SHIMADZU Nexera-i LC-2040C 3D High Plus liquid chromatograph equipped with a DAD/FLD detector and Kinetex® F5 column 100 Å (2.6 mm; 150×4.6 mm; Phenomenex, Torrance, CA, USA) maintained at 30 °C. The mixture of acetonitrile and water 50/50 (V/V) was used as the mobile phase under isocratic conditions, with a flow rate of 1 cm^3min^{-1}. The detection wavelength was 210 nm. Injections of 50 μL were repeated at least three times, and the result was averaged for each sample. The collected data were acquired and processed using a LabSolutions/LC Solution System. The concentration of acid from the NSAIDs group and the amino acid isopropyl ester salts in the aqueous phase were calculated based on peak area measurements using a calibration curve method, while the concentration in the n-octanol phase was calculated in accordance with the formula:

$$C_O = C_0 - C_W$$

where

C_O−concentration of the substance dissolved in the octanol layer [$mg \cdot dm^{-3}$]

C_W−concentration of the substance dissolved in the aqueous layer [$mg \cdot dm^{-3}$]

C_0−total concentration of the substance used in the experiment [$mg \cdot dm^{-3}$]

The partition coefficient $logP_{ow}$ was calculated by the equation:

$$log\mathrm{P}_{\mathrm{OW}} = log_{C_O} - log_{C_W}$$

Solubility

The modified Vogel's method was used to evaluate the solubility in selected polar and nonpolar organic solvents at room temperature. According to this method, the compounds were classified as soluble, partially soluble, or insoluble. Ethanol, dimethyl sulfoxide, dichloromethane, chloroform, ethyl acetate, diethyl ether, toluene, and n-hexane were used as solvents. The solubility (saturation concentration), defined as the maximum amount of a substance that can be dissolved at a given temperature per unit volume of solvent, was determined in deionized water and phosphate buffers (pH 5.4 and 7.4). An excess of the compound was added to 2 cm^3 of water or buffer solution in a screwed vial and stirred vigorously at 25.00 ± 0.05 °C for 24 hours, followed by centrifugation. Then, the supernatant was withdrawn and diluted. The concentration of the analyzed compound was determined by using the HPLC method. The conditions of HPLC analysis are detailed in the partition coefficient part.

General procedure for preparation of amino acid isopropyl hydrochlorides [AAOiPr][HCl]

L-amino acids isopropyl ester hydrochlorides were obtained following the previously described procedure [1]. First, 5 g of amino acid was dispersed into 50 mL of isopropyl alcohol at room temperature, followed by adding two molar equivalents of TMSCl into the mixture. The solution was stirred thoroughly at 60ºC for 24 h for the complete conversion of reagents, which was observed by the dissolution of substrates. After that, the product was purified from excess TMSCl and alcohol by evaporation at 60°C under a vacuum. Then, the formed by-products TMSOH or TMSOiPr were removed by washing with diethyl ether. The product was subsequently dissolved in chloroform and filtered under reduced pressure to purify it from unreacted amino acids. Next, the filtrate was distilled off under reduced pressure (60°C, 10 mbar). Finally, the obtained hydrochloride was dried in a vacuum dryer at 60°C, 5 mbar for 24 h. As a result, the L-amino acid isopropyl ester hydrochloride (AAOiPr·HCl) was obtained with a good yield (95-99%).

General procedure for preparation of amino acid isopropyl esters [AAOiPr]

Obtained in the first step, [AAOiPr][HCl] was added to a small amount of distilled water and neutralized by adding three molar equivalents of 25% ammonium hydroxide aqueous solution. The solution was intensively mixed, and then the product was extracted with the organic solvent. The ethyl acetate was used for extraction to separate L-threonine isopropyl ester, while all other L-amino acid esters were extracted using diethyl ether. The organic layer was dried using anhydrous Na_2SO_4 and then concentrated under a vacuum to receive [AAOiPr].

General procedure for preparation of amino acid isopropyl esters salts

The equimolar reaction of L-amino acid isopropyl ester with proper acid (ibuprofen, ketoprofen, naproxen, or salicylic acid) was carried out in chloroform at room temperature for 20 minutes. The solvent was then evaporated under a vacuum at 30°C. The obtained L-amino acids isopropyl derivatives were dried for 24 h at 50 °C under reduced pressure.

The NMR spectra of [AAOiPr][IBU]

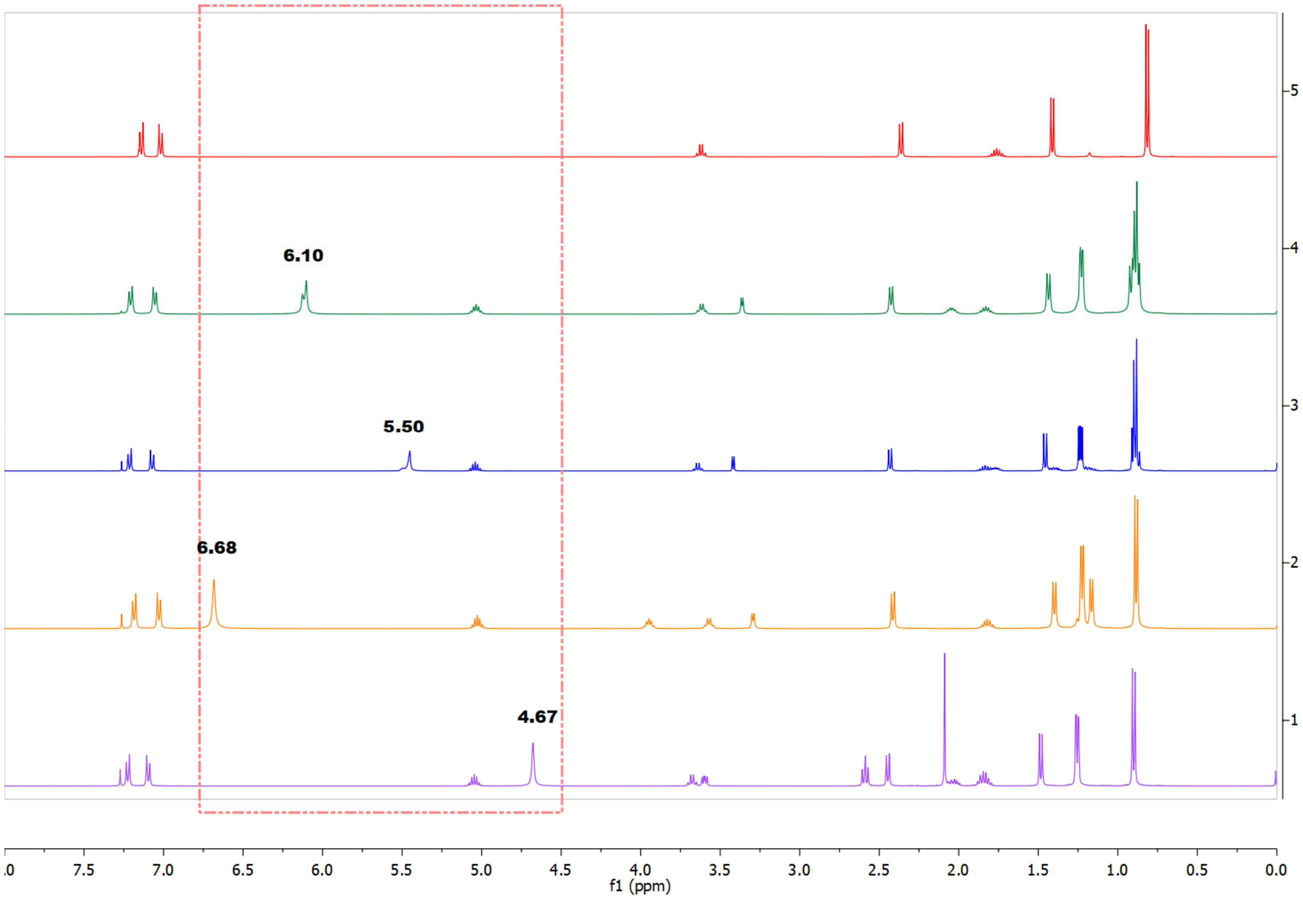

Figure S1. ^{1}H NMR spectra of ibuprofen and its derivatives (in the red dot square, the protonated amino groups are marked)—from the top: IBU, [L-ValOiPr][IBU], [L-IleOiPr][IBU], [L-ThrOiPr][IBU], and [L-MetOiPr][IBU].

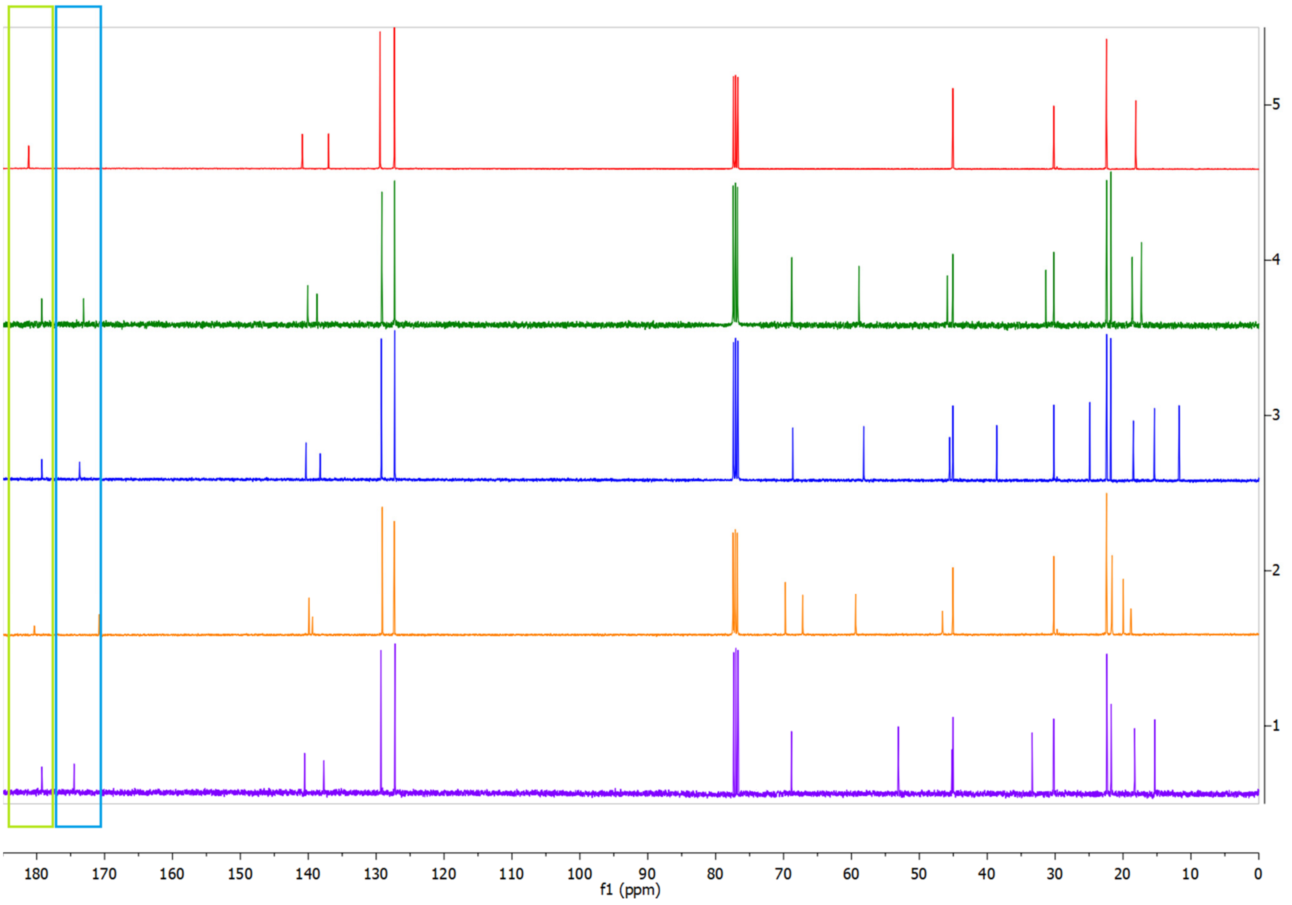

Figure S2. ^{13}C NMR spectra of ibuprofen and its derivatives (the carbonyl group from ibuprofen is marked in the green square, and from amino acid isopropyl esters are marked in blue square)—from the top: IBU, [L-ValOiPr][IBU], [L-IleOiPr][IBU], [L-ThrOiPr][IBU], and [L-MetOiPr][IBU].

The NMR spectra of [AAOiPr][KETO]

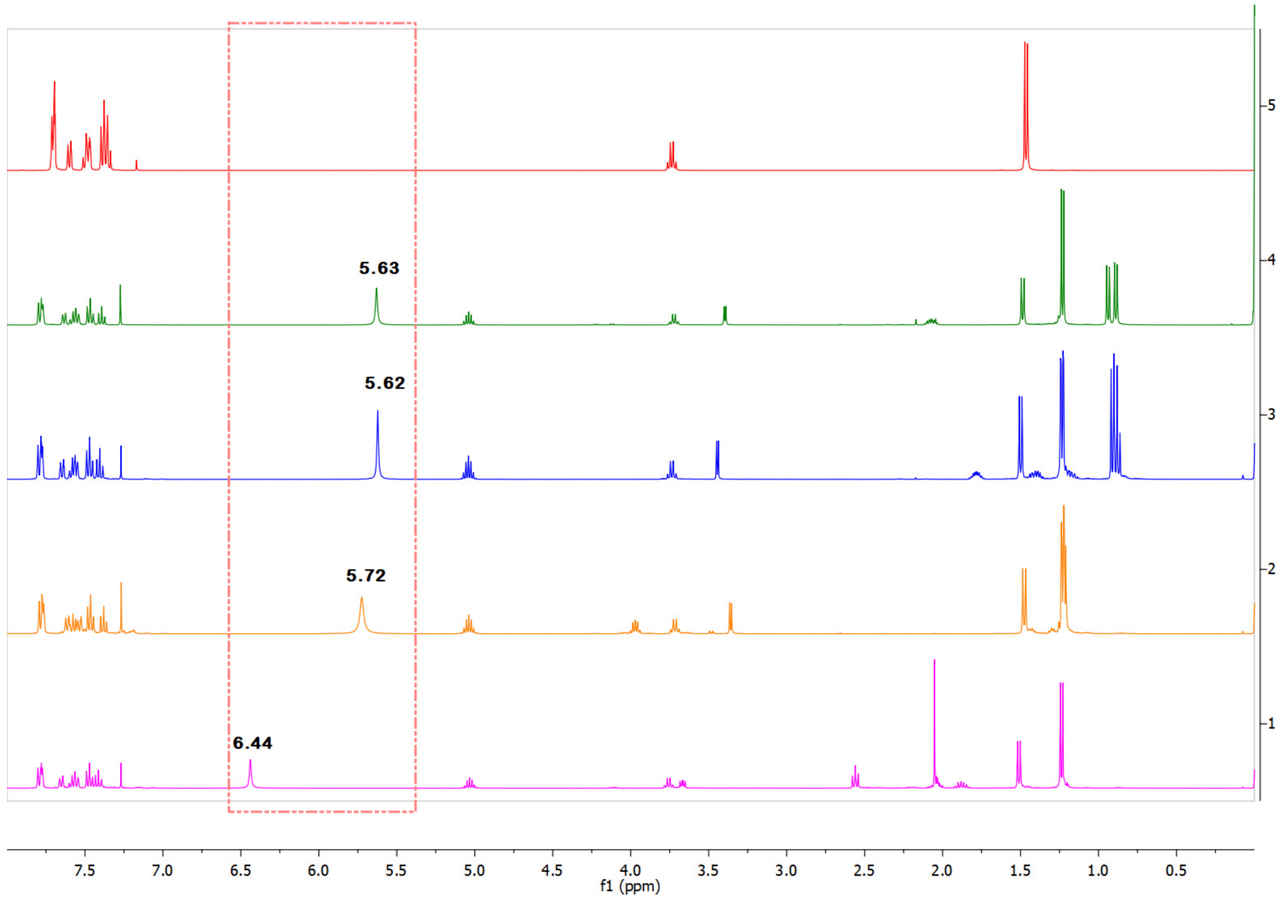

Figure S3. ^{1}H NMR spectra of ketoprofen and its derivatives (in the red dot square, the protonated amino groups are marked)—from the top: KETO, [L-ValOiPr][KETO], [L-IleOiPr][KETO], [L-ThrOiPr][KETO], and [L-MetOiPr][KETO].

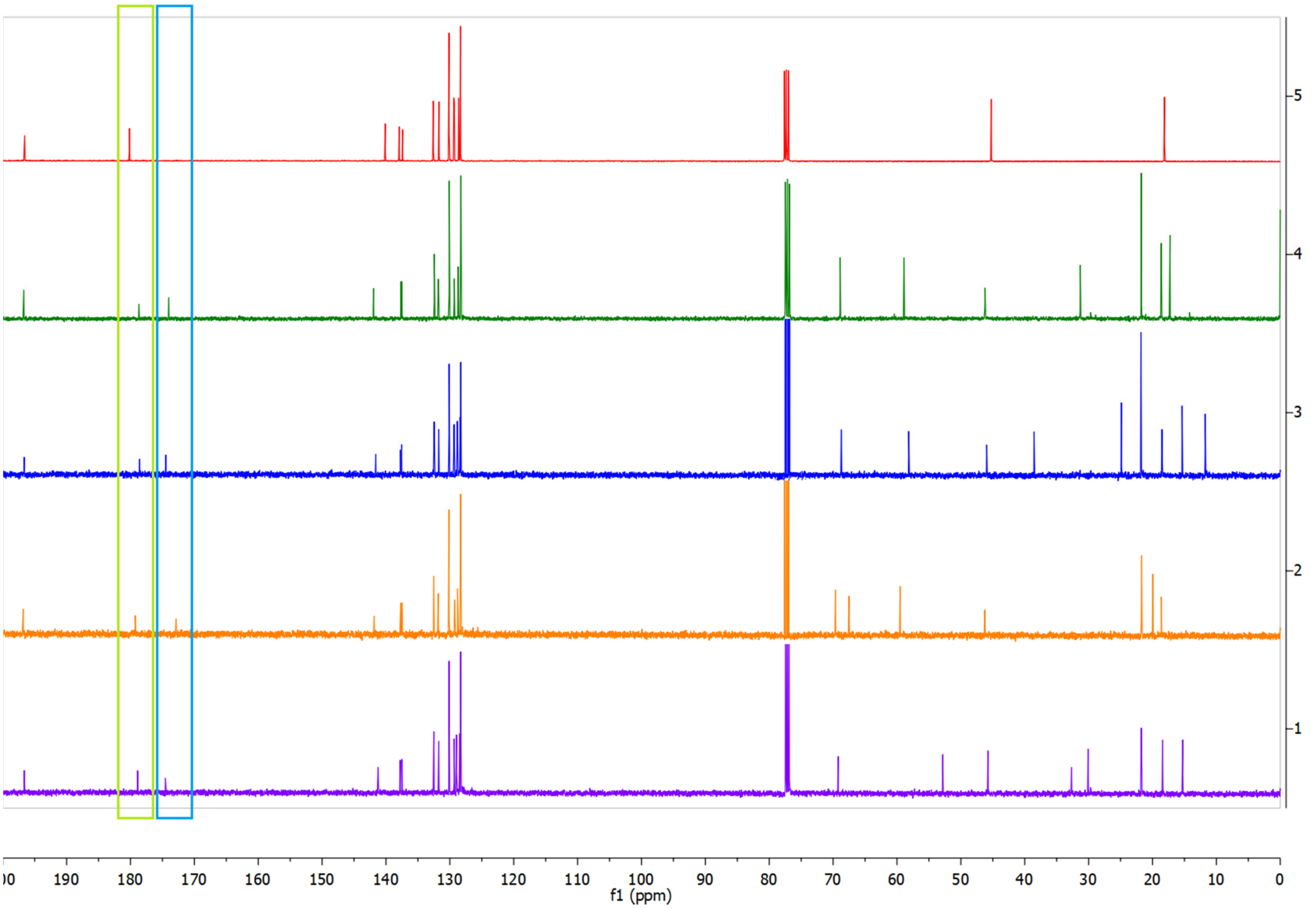

Figure S4. ^{13}C NMR spectra of ketoprofen and its derivatives (the carbonyl group from ketoprofen is marked in the green square, and from amino acid isopropyl esters are marked in blue square)—from the top: KETO, [L-ValOiPr][KETO], [L-IleOiPr][KETO], [L-ThrOiPr][KETO], and [L-MetOiPr][KETO].

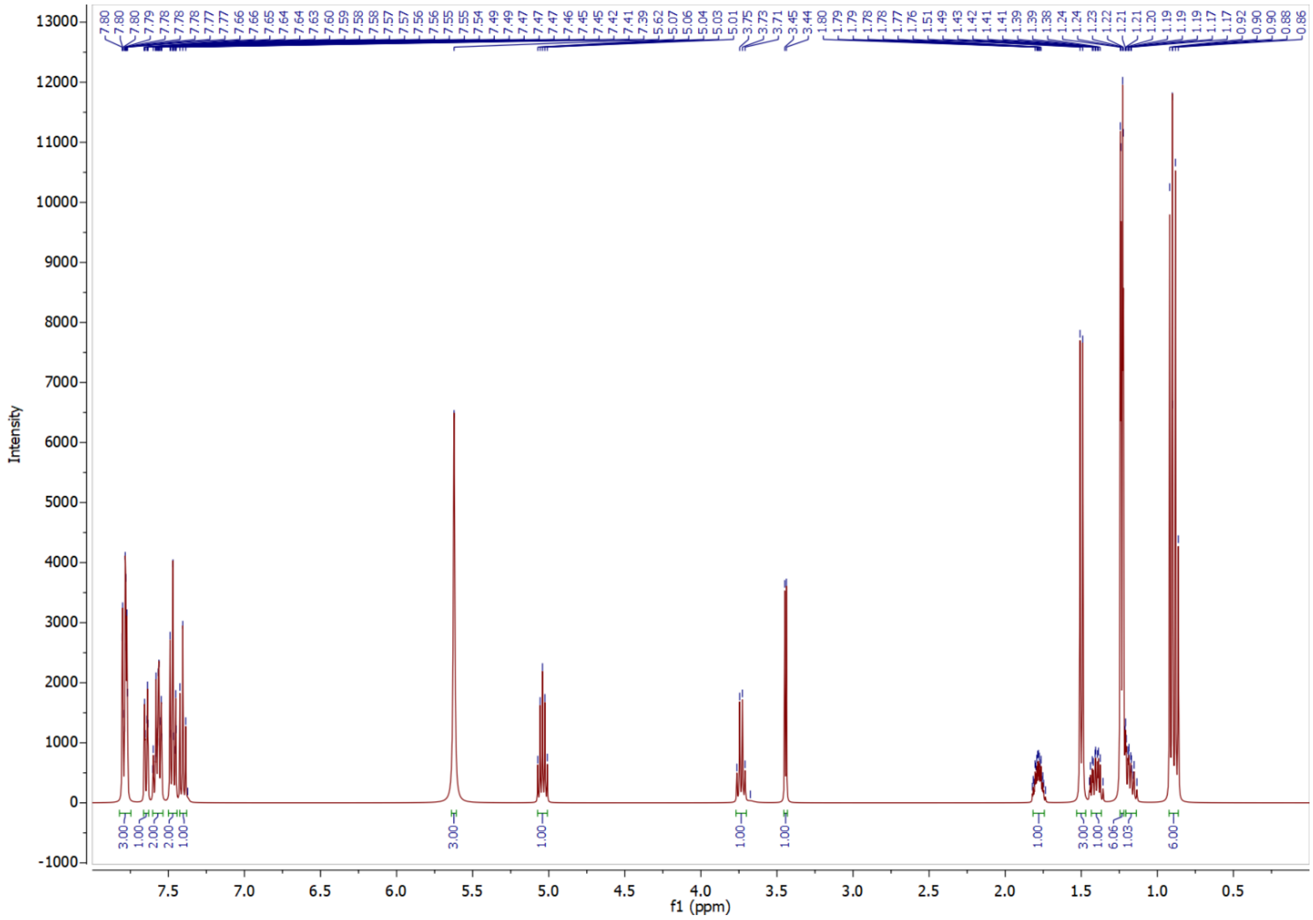

Figure S5. ^1H NMR spectrum of [L-IleOiPr][KETO].

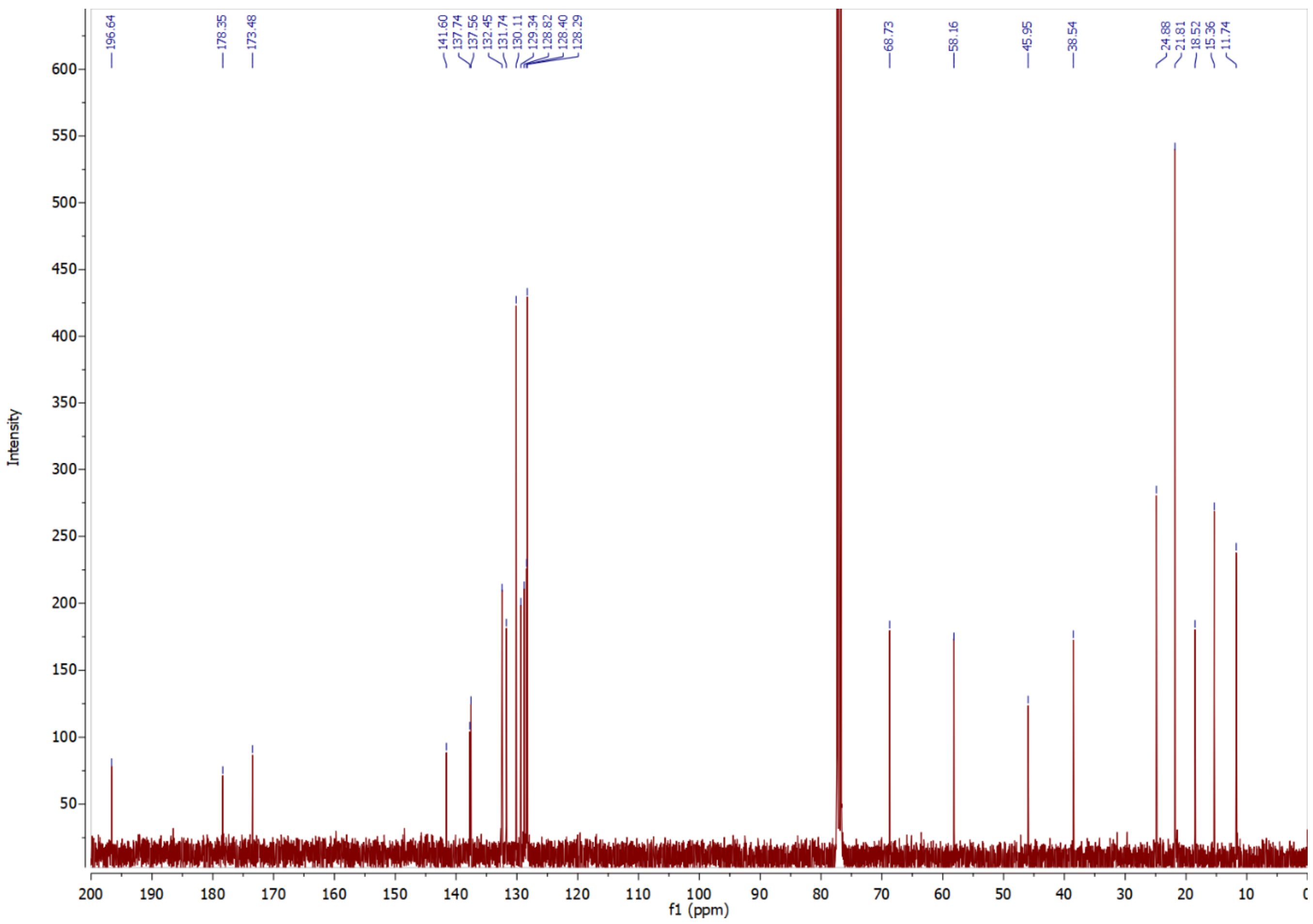

Figure S6. ^{13}C NMR spectrum of [L-IleOiPr][KETO].

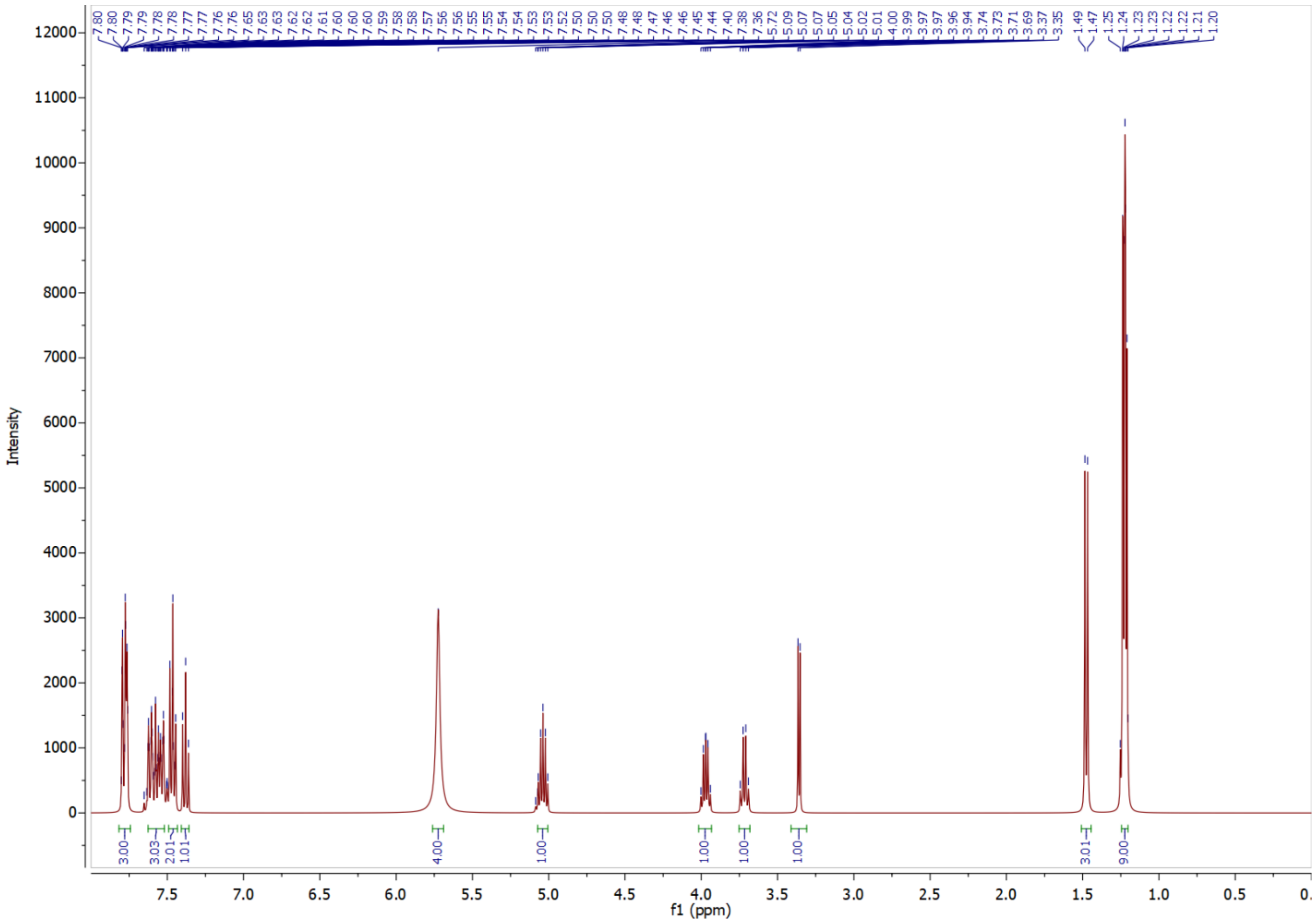

Figure S7. ^{1}H NMR spectrum of [L-ThrOiPr][KETO].

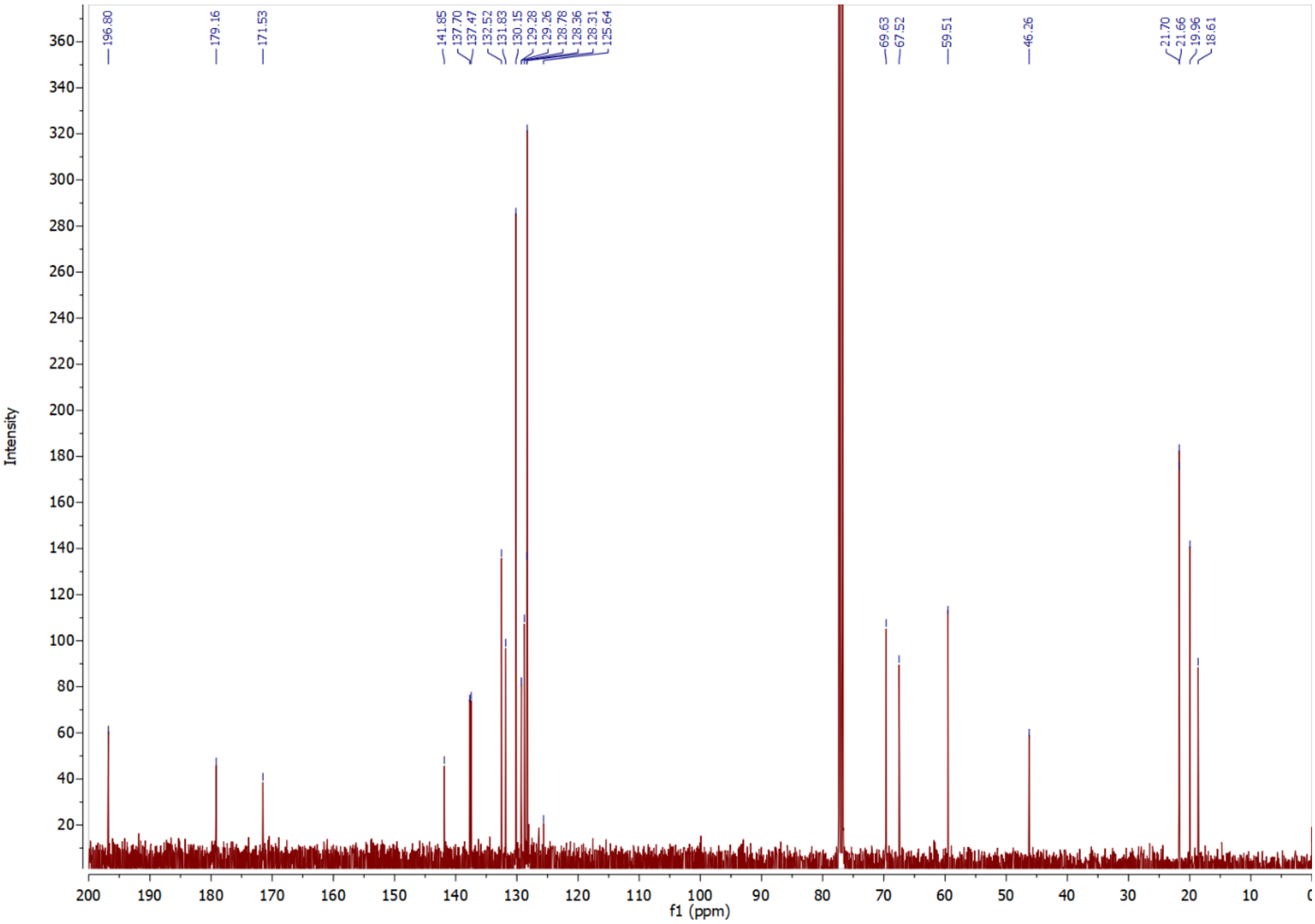

Figure S8. ^{13}C NMR spectrum of [L-ThrOiPr][KETO].

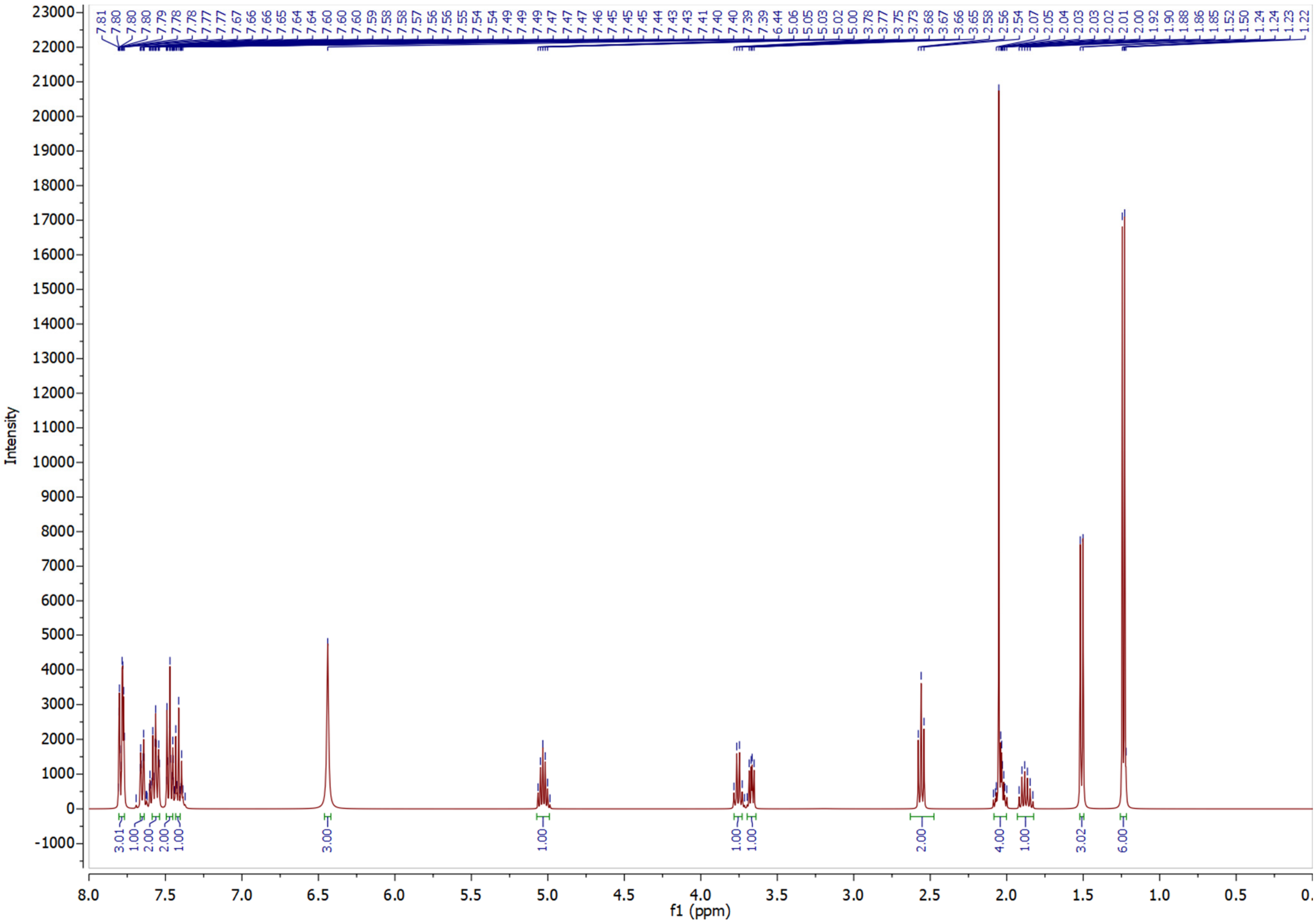

Figure S9. ^{1}H NMR spectrum of [L-MetOiPr][KETO].

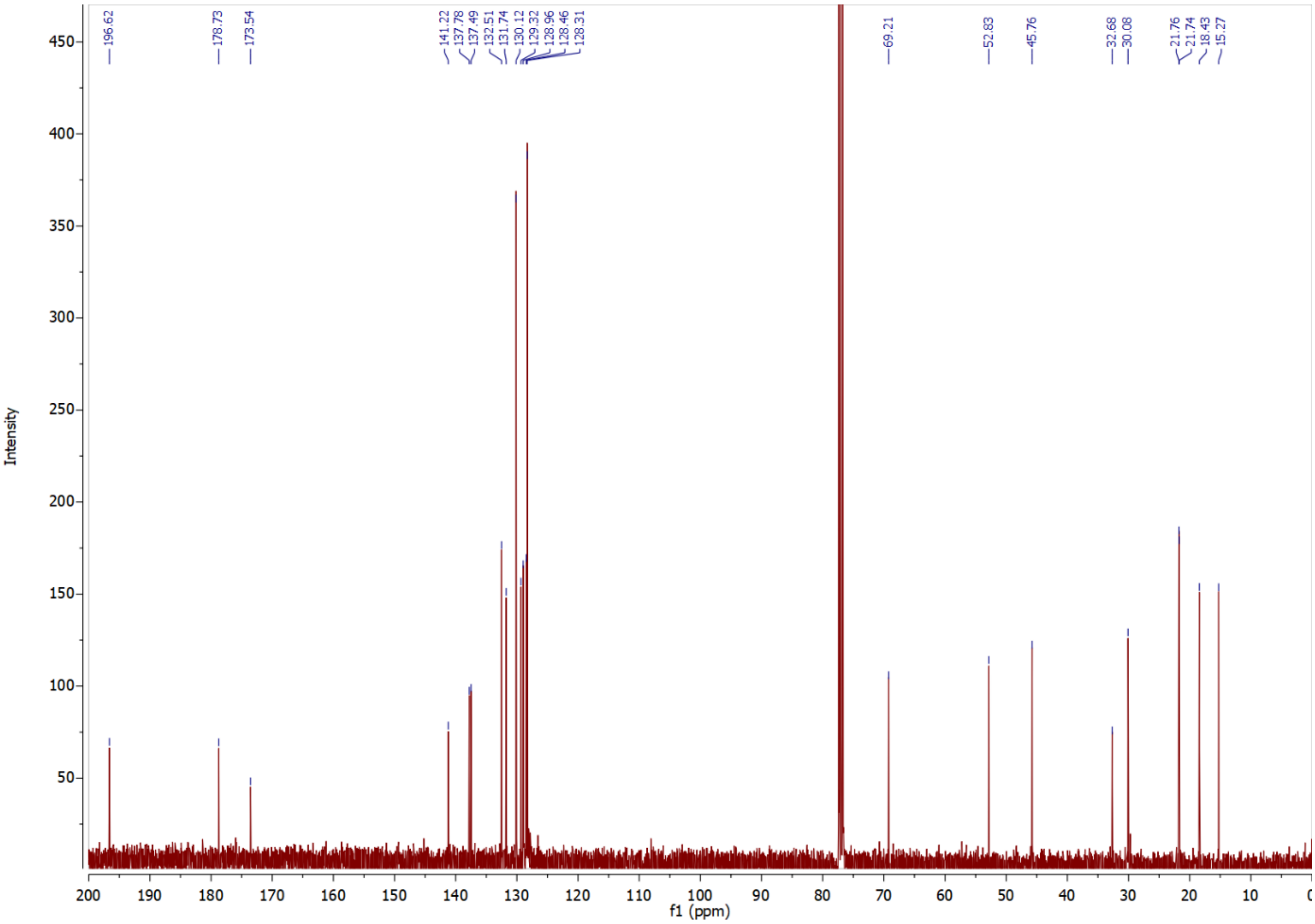

Figure S10. ^{13}C NMR spectrum of [L-MetOiPr][KETO].

The NMR spectra of [AAOiPr][NAP]

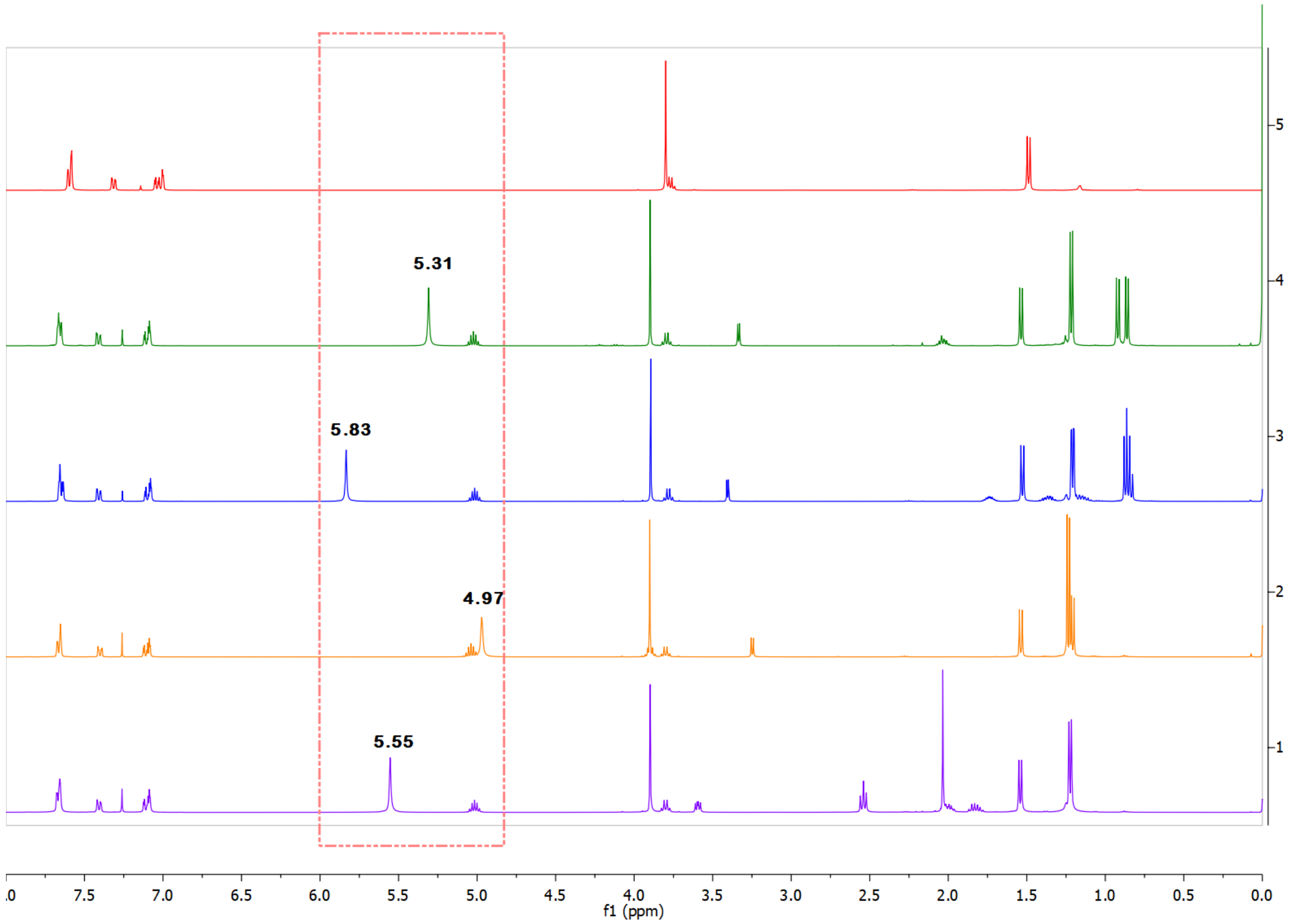

Figure S11. ^{1}H NMR spectra of naproxen and its derivatives (in the red dot square, the protonated amino groups are marked)—from the top: NAP, [L-ValOiPr][NAP], [L-IleOiPr][NAP], [L-ThrOiPr][NAP], and [L-MetOiPr][NAP].

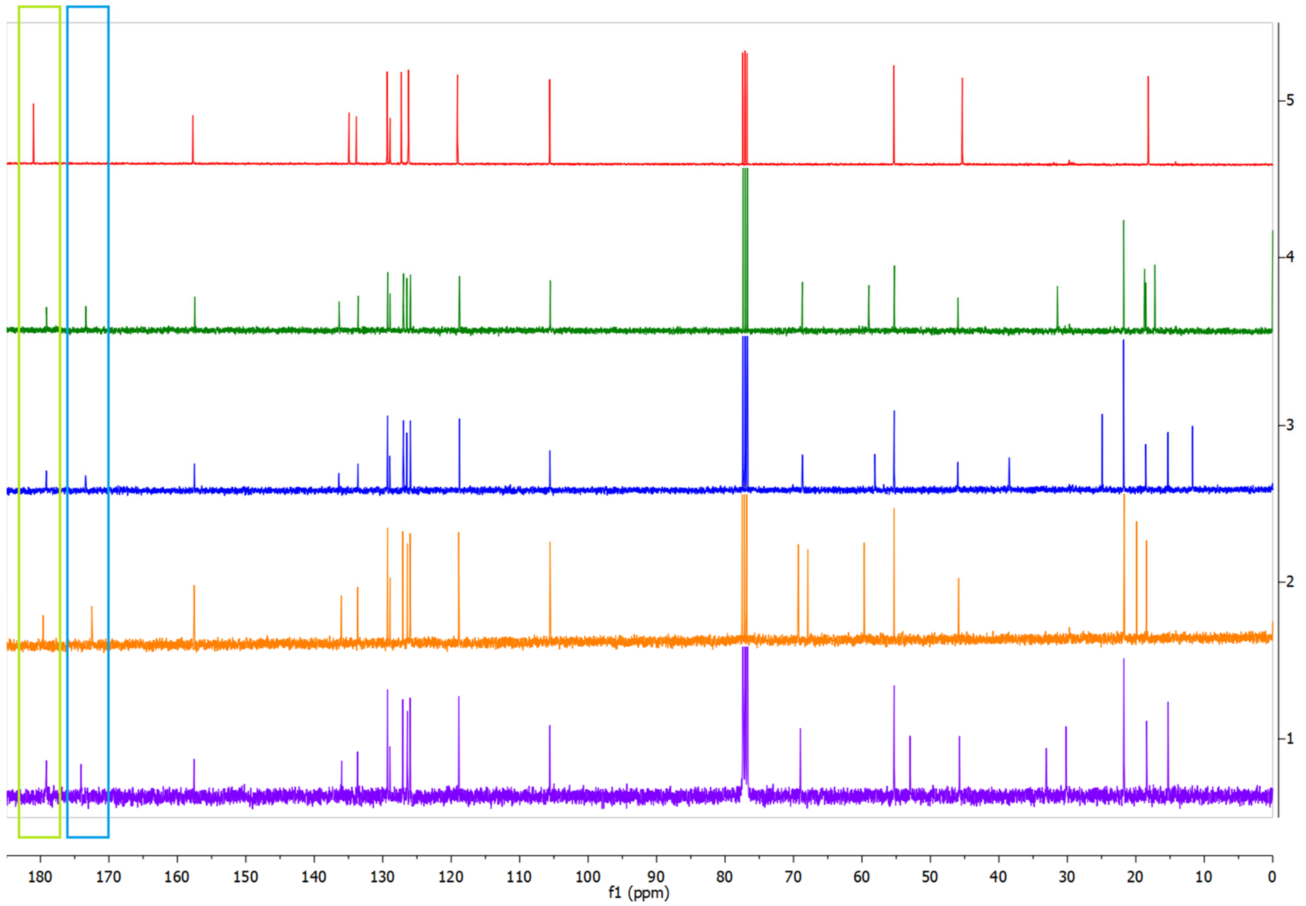

Figure S12. ^{13}C NMR spectra of naproxen and its derivatives (the carbonyl group from naproxen is marked in the green square, and from amino acid isopropyl esters are marked in blue square)—from the top: NAP, [L-ValOiPr][NAP], [L-IleOiPr][NAP], [L-ThrOiPr][NAP], and [L-MetOiPr][NAP].

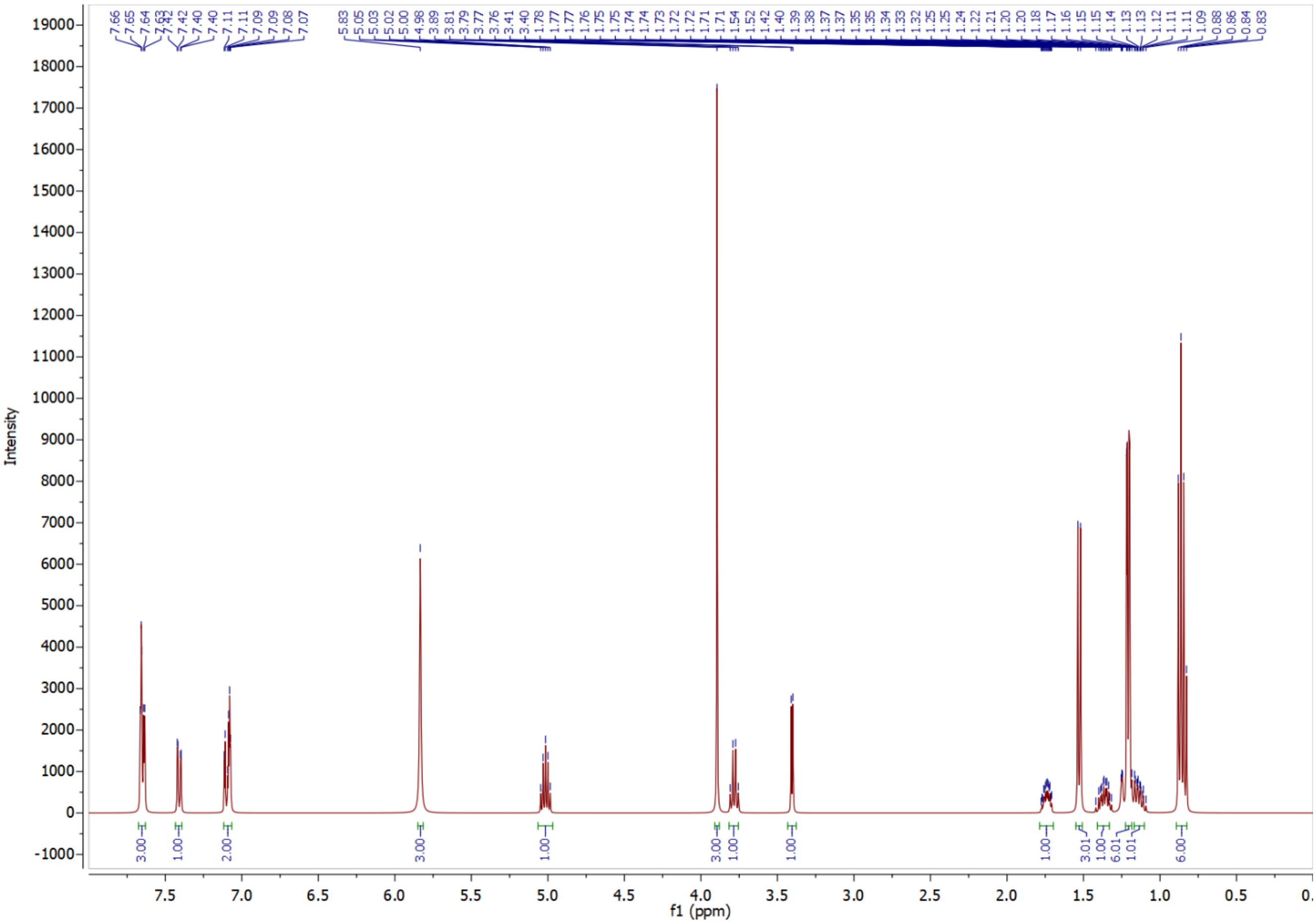

Figure S13. ^{1}H NMR spectrum of [L-IleOiPr][NAP].

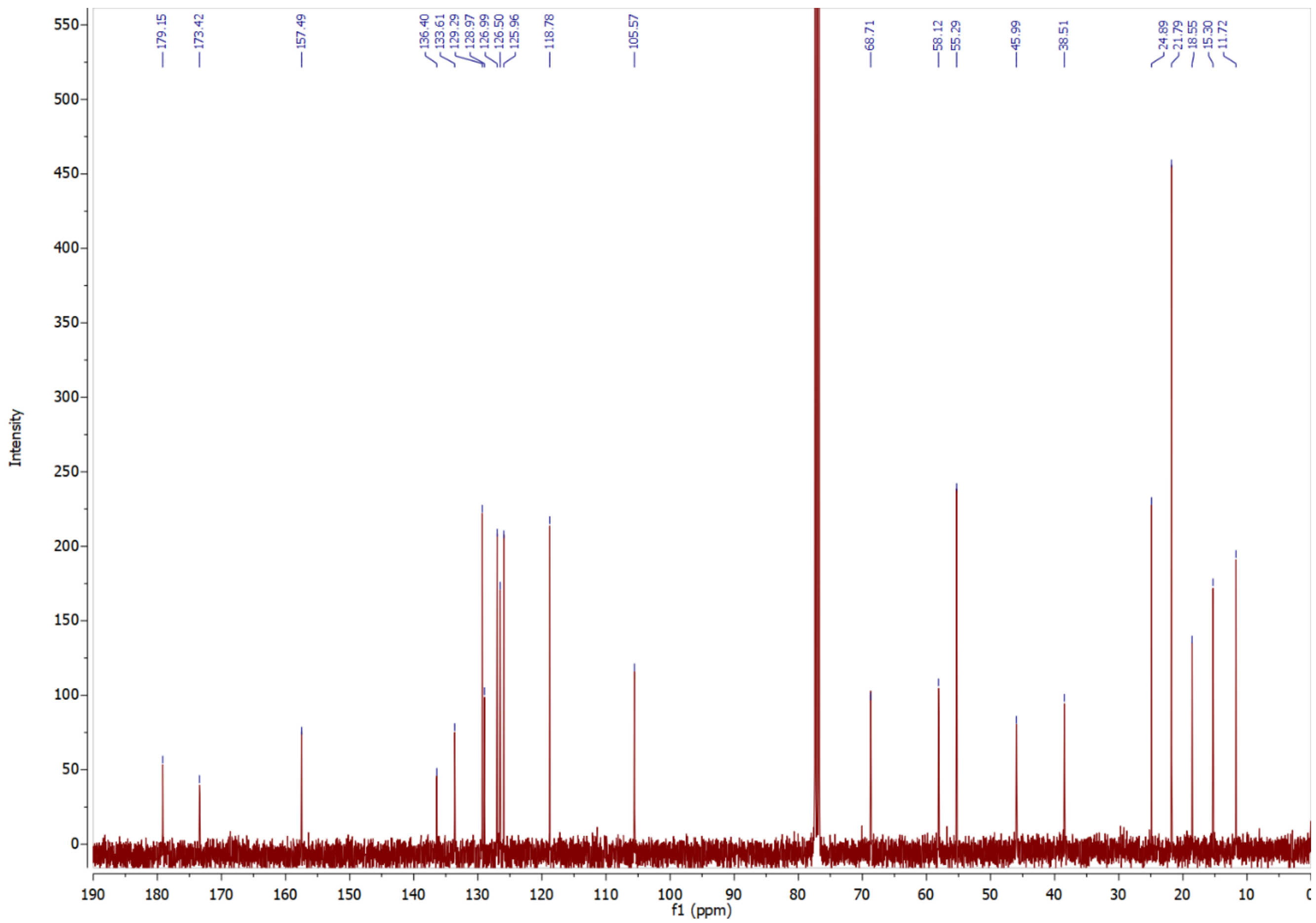

Figure S14. ^{13}C NMR spectrum of [L-IleOiPr][NAP].

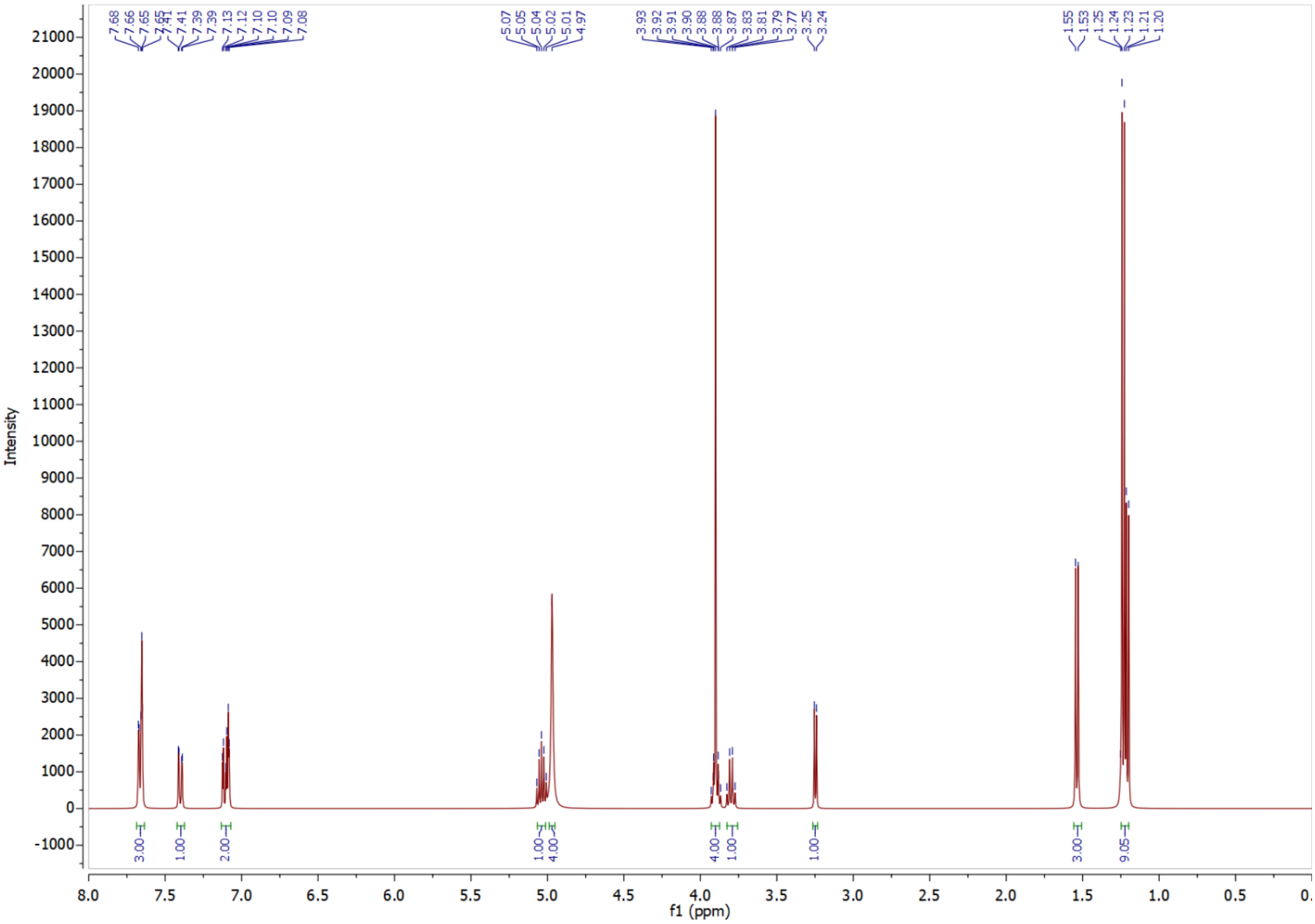

Figure S15. ^{1}H NMR spectrum of [L-ThrOiPr][NAP].

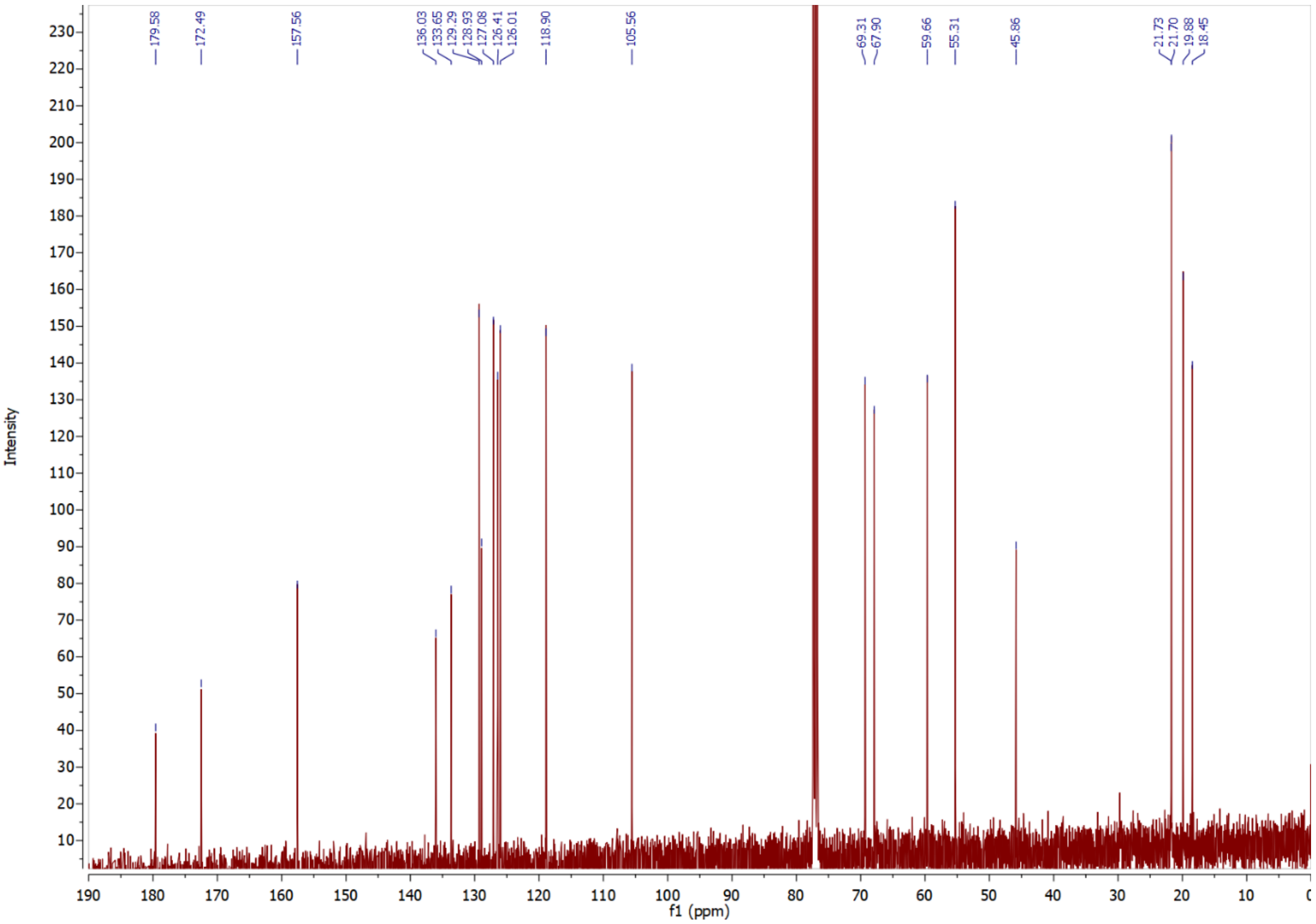

Figure S16. ^{13}C NMR spectrum of [L-ThrOiPr][NAP].

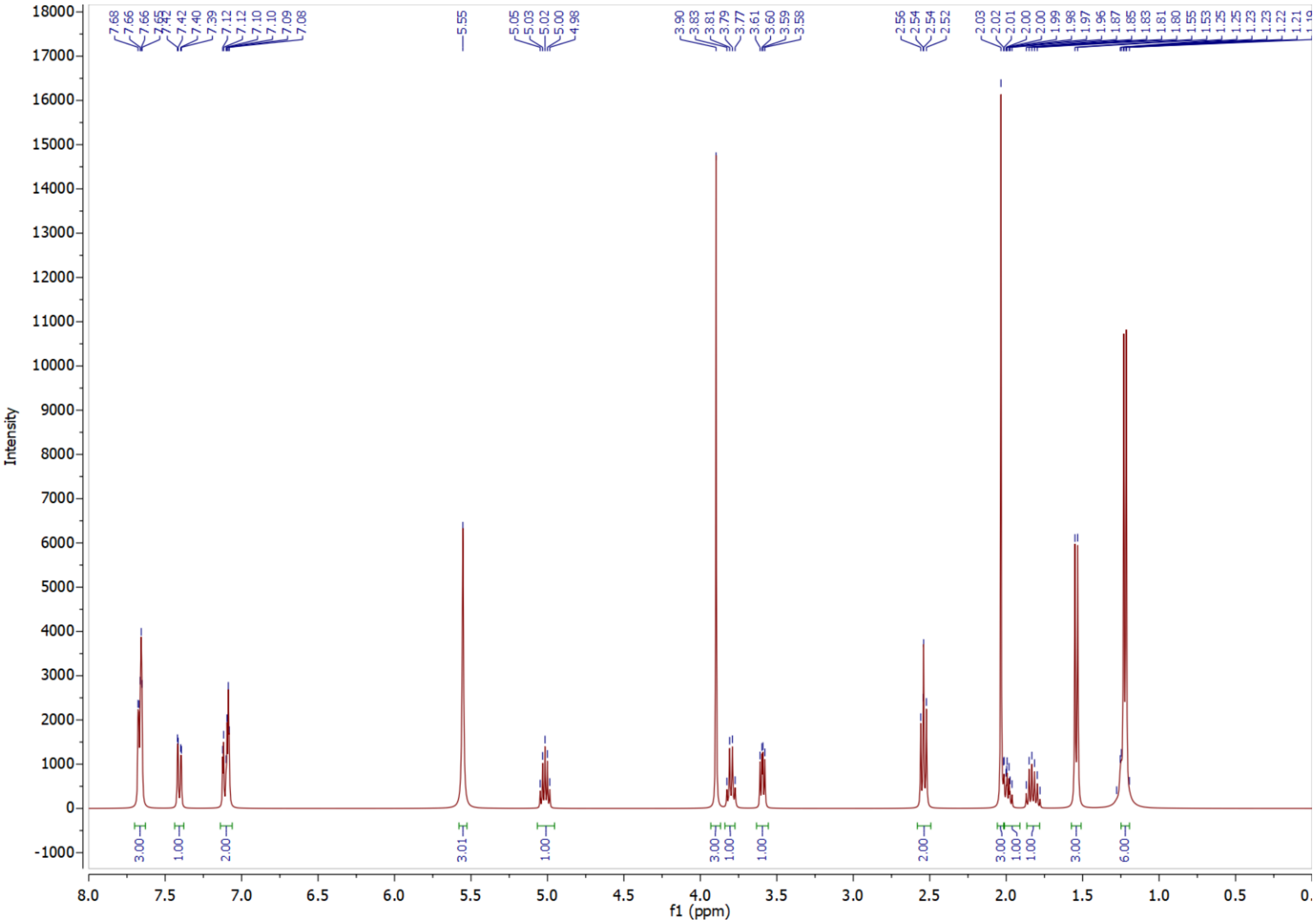

Figure S17. ^{1}H NMR spectrum of [L-MetOiPr][NAP].

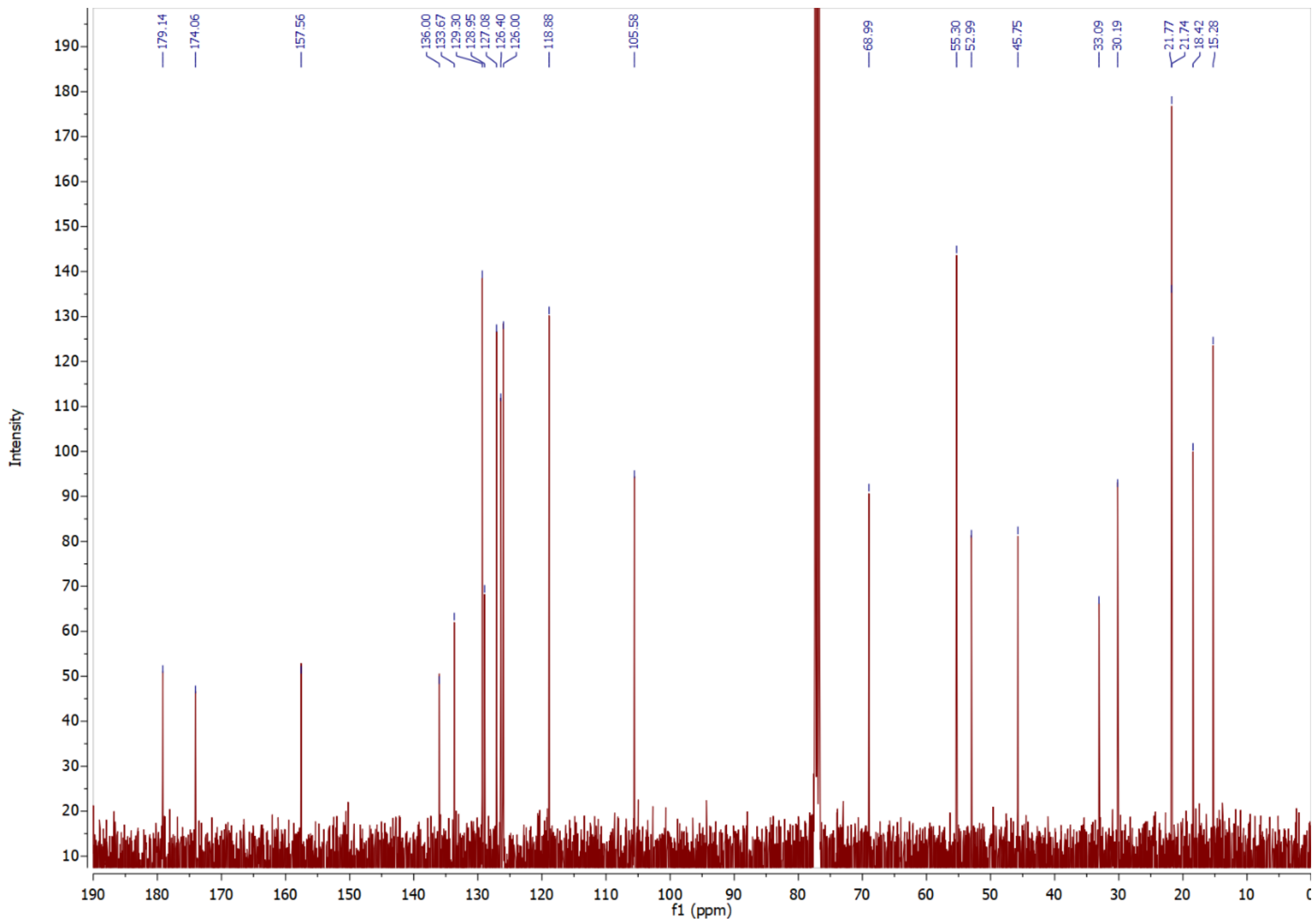

Figure S18. ^{13}C NMR spectrum of [L-MetOiPr][NAP].

The NMR spectra of [AAOiPr][SA]

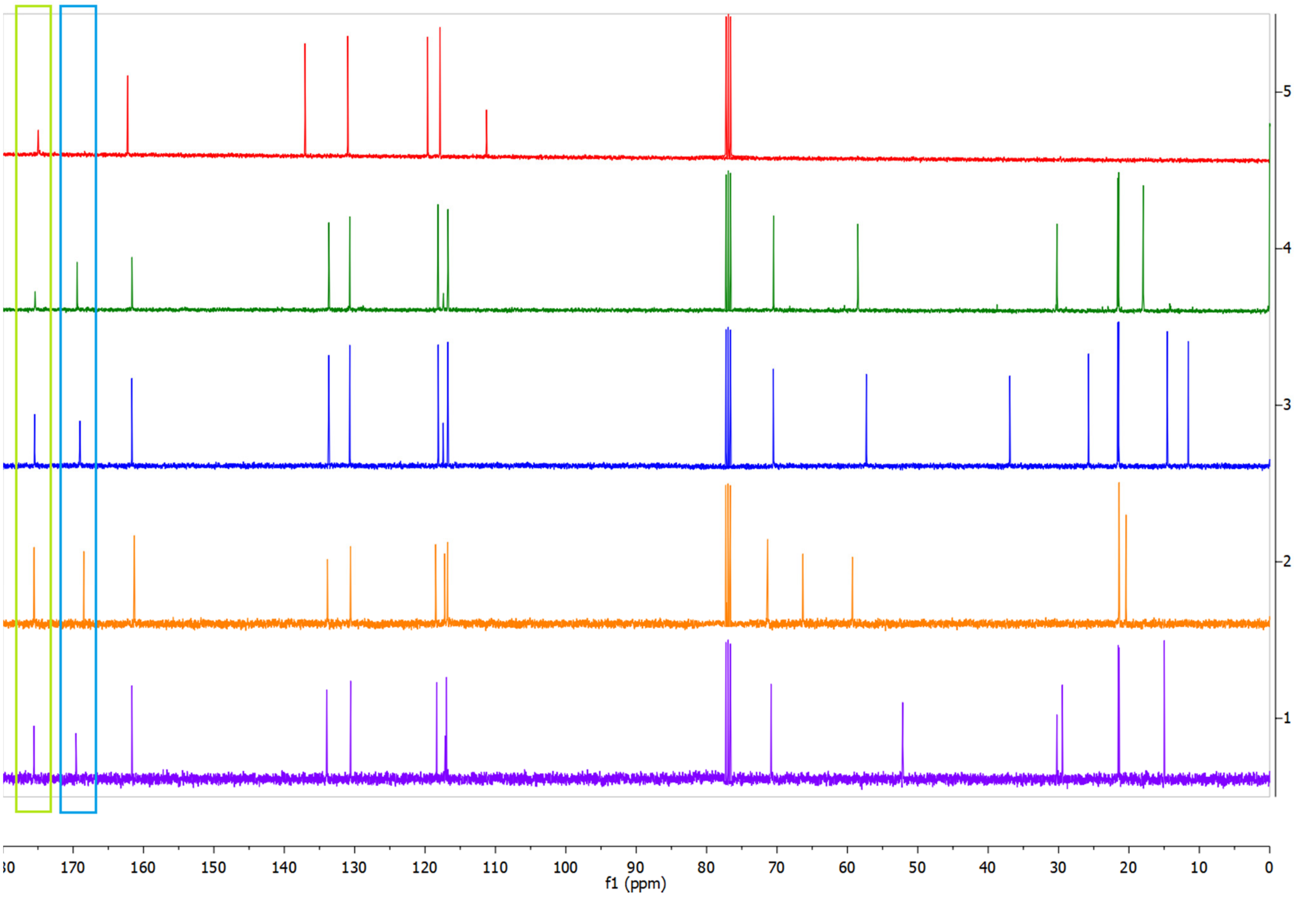

Figure S19. ^{13}C NMR spectra of salicylic acid and its derivatives (the carbonyl group from salicylic acid is marked in the green square, and from amino acid isopropyl esters are marked in blue square)—from the top: SA, [L-ValOiPr][SA], [L-IleOiPr][SA], [L-ThrOiPr][SA], and [L-MetOiPr][SA].

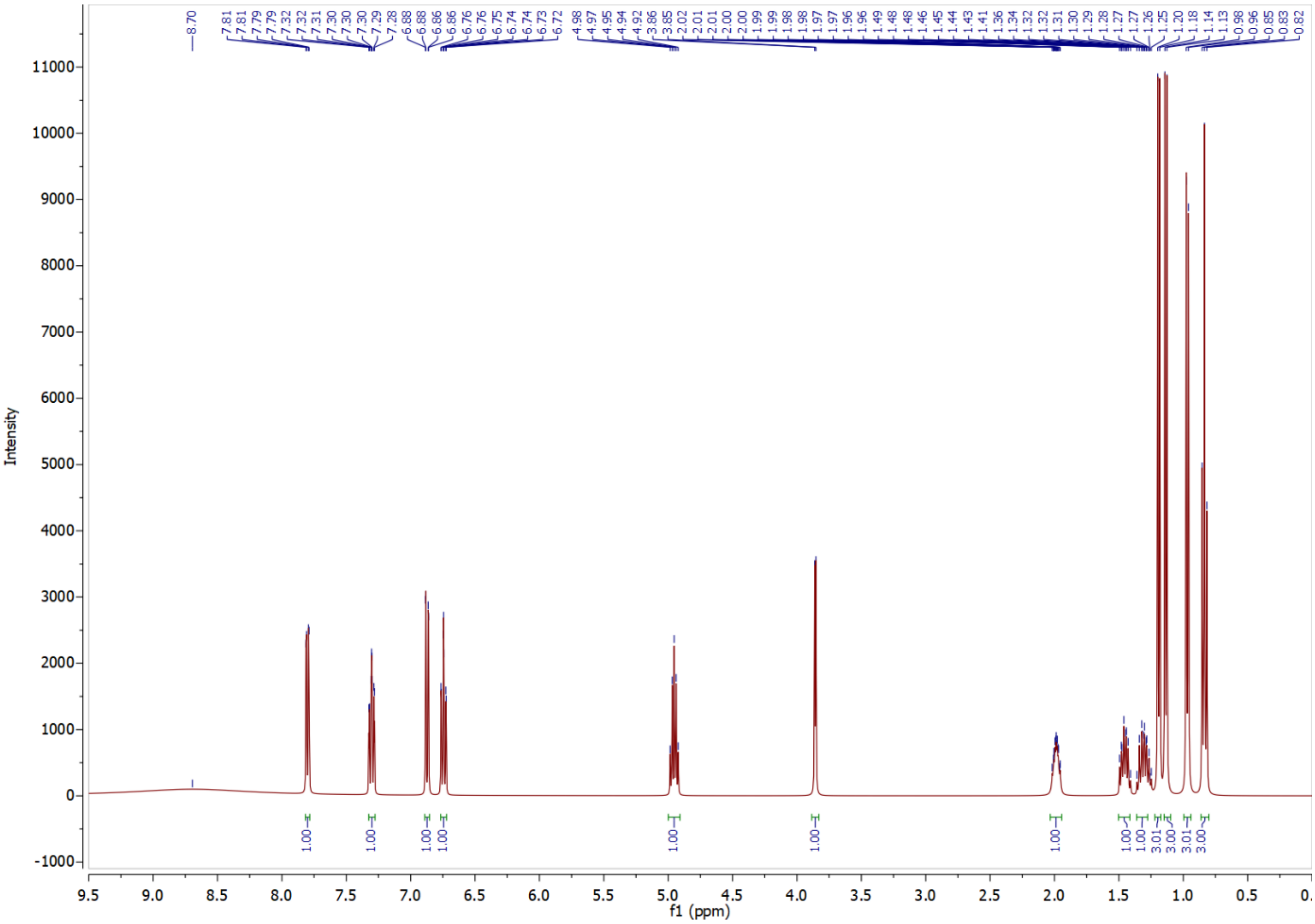

Figure S20. ^{1}H NMR spectrum of [L-IleOiPr][SA].

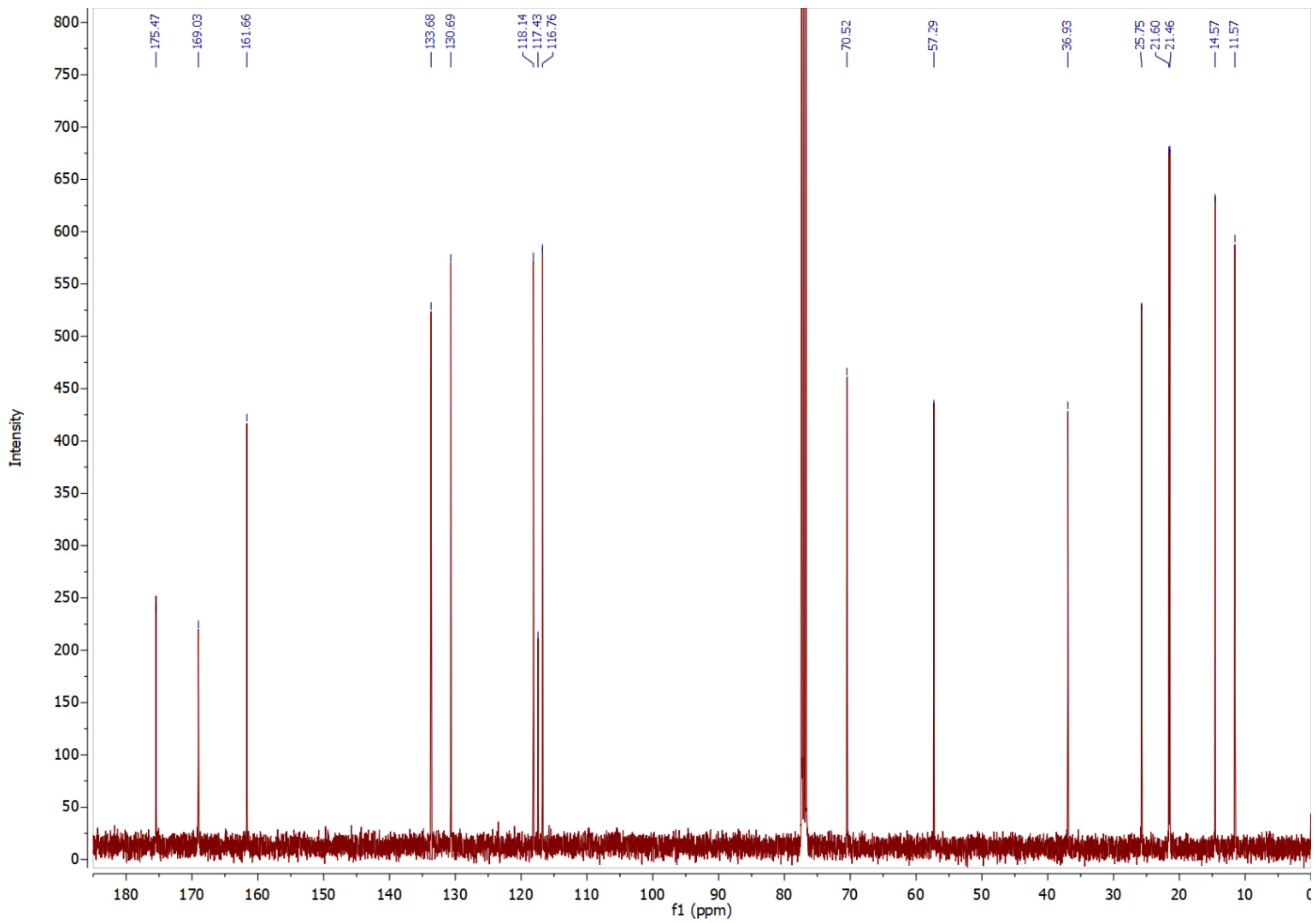

Figure S21. ^{13}C NMR spectrum of [L-IleOiPr][SA].

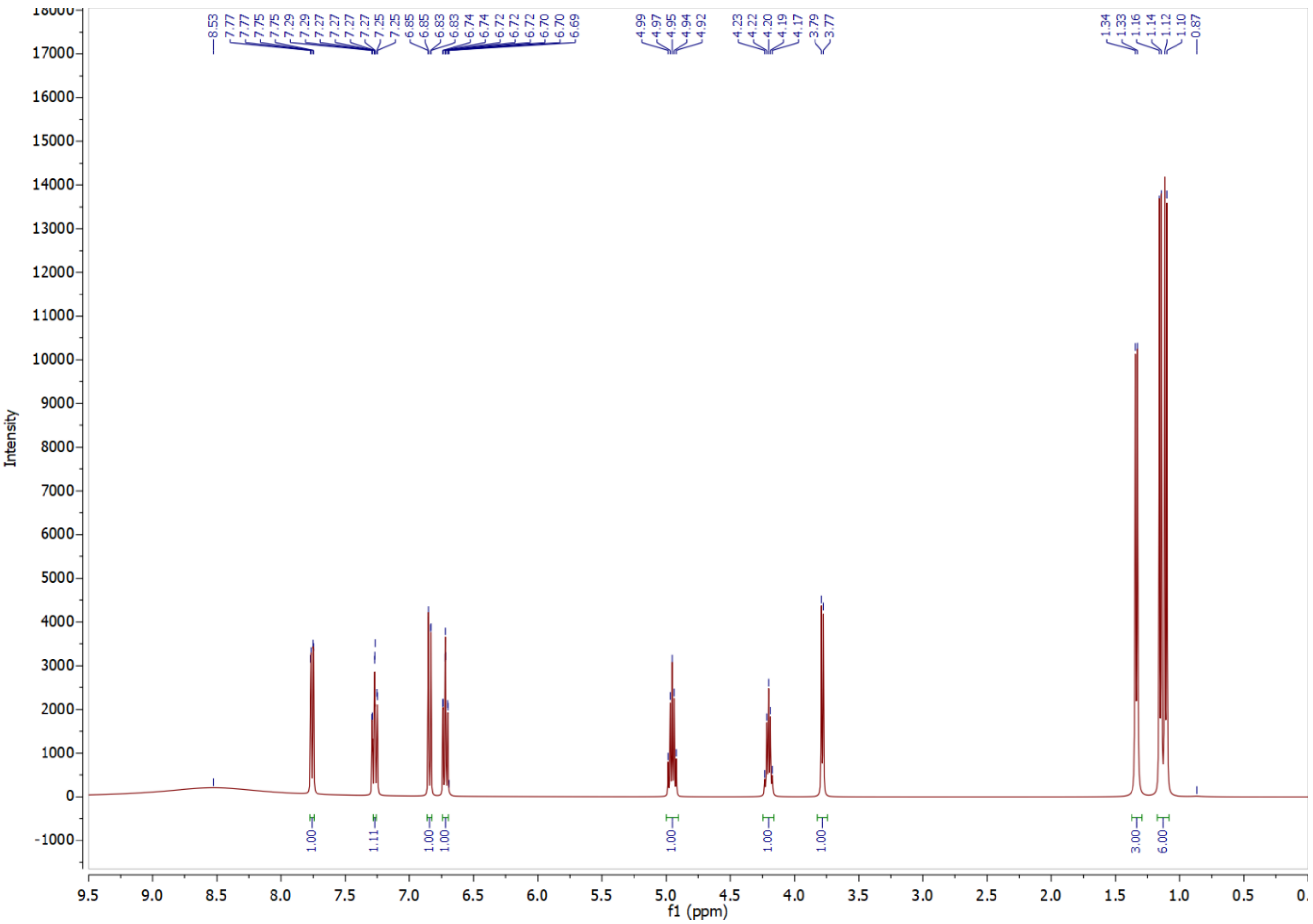

Figure S22. ^{1}H NMR spectrum of [L-ThrOiPr][SA].

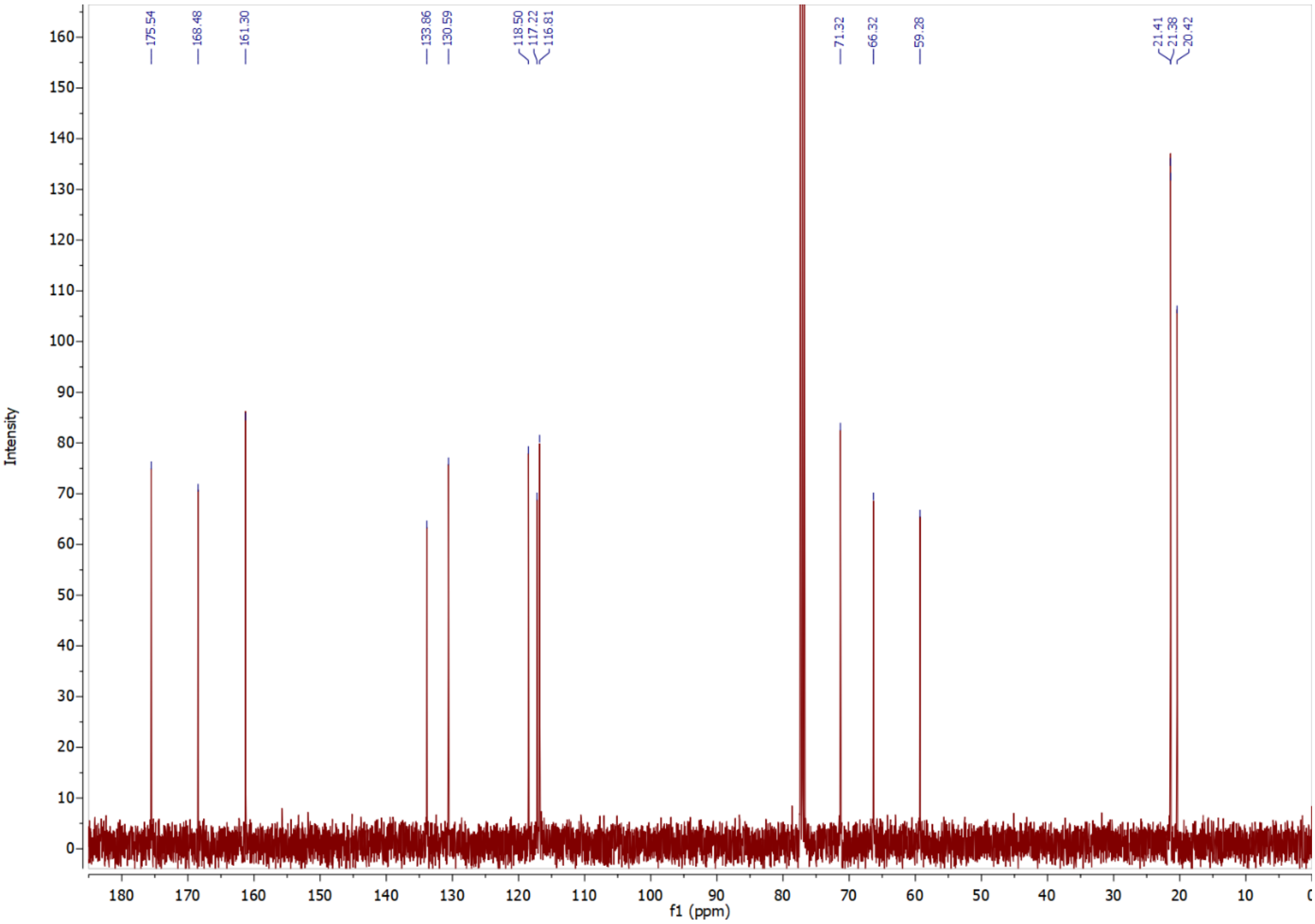

Figure S23. ^{13}C NMR spectrum of [L-ThrOiPr][SA].

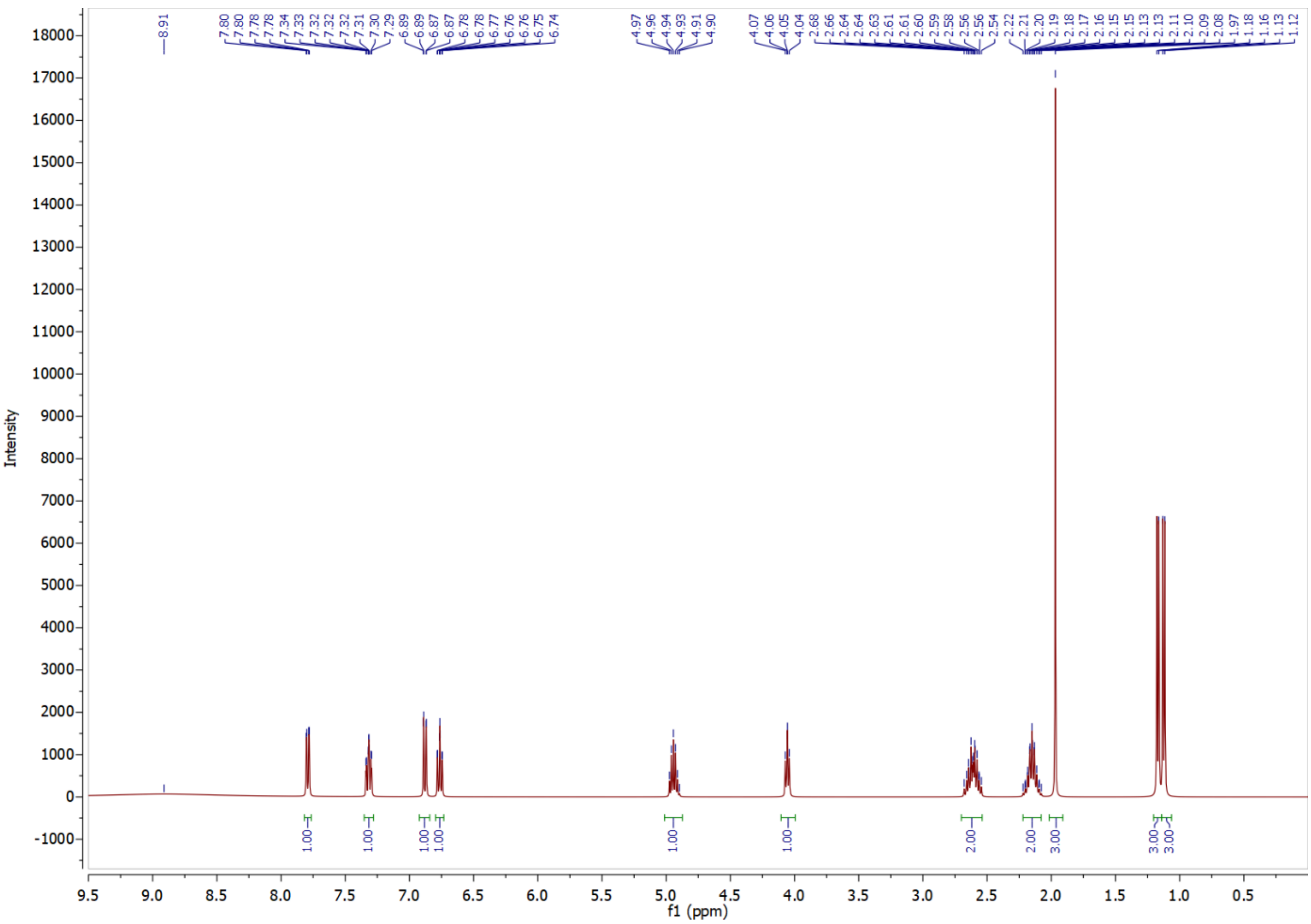

Figure S24. ^{1}H NMR spectrum of [L-MetOiPr][SA].

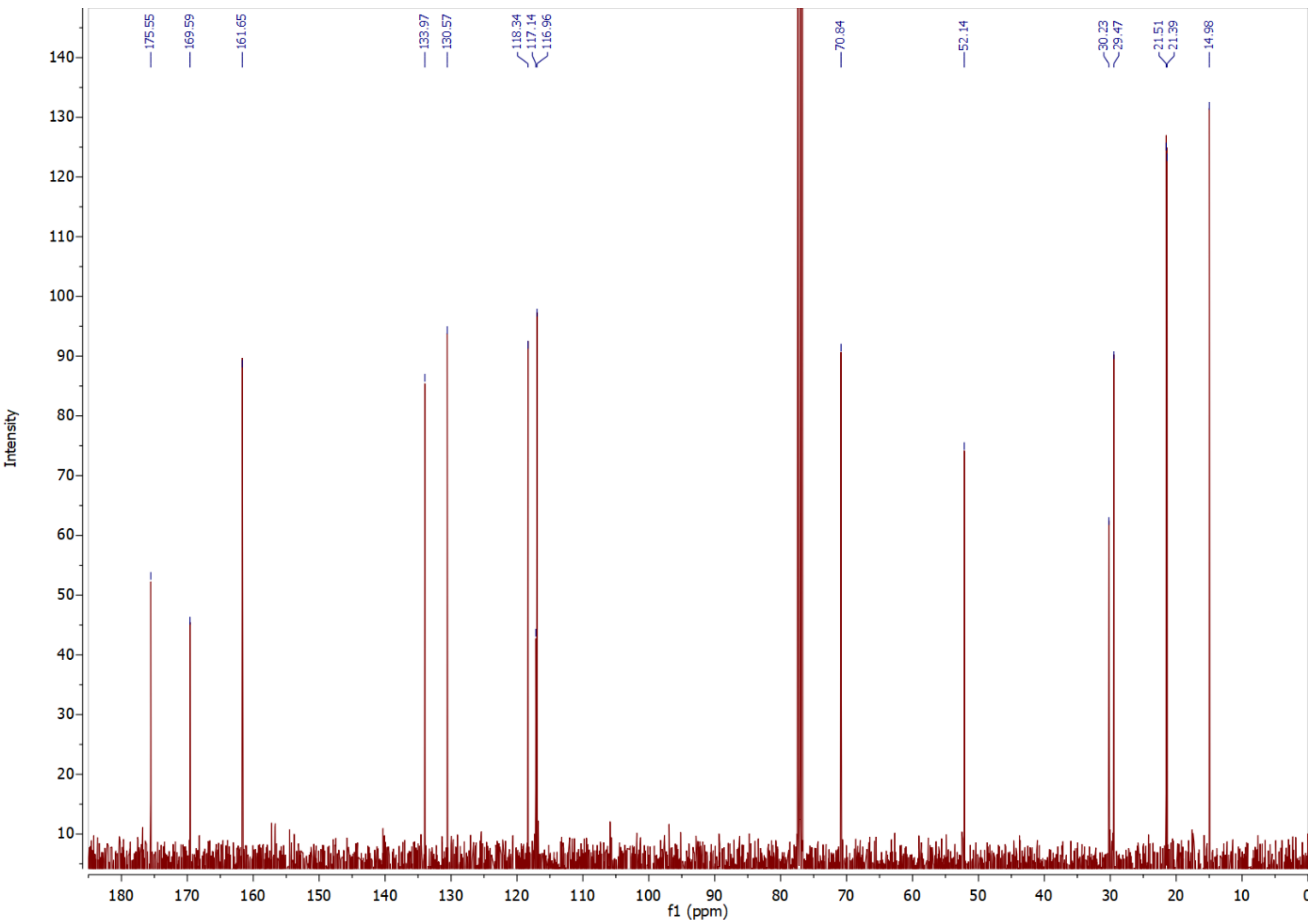

Figure S25. ^{13}C NMR spectrum of [L-MetOiPr][SA].

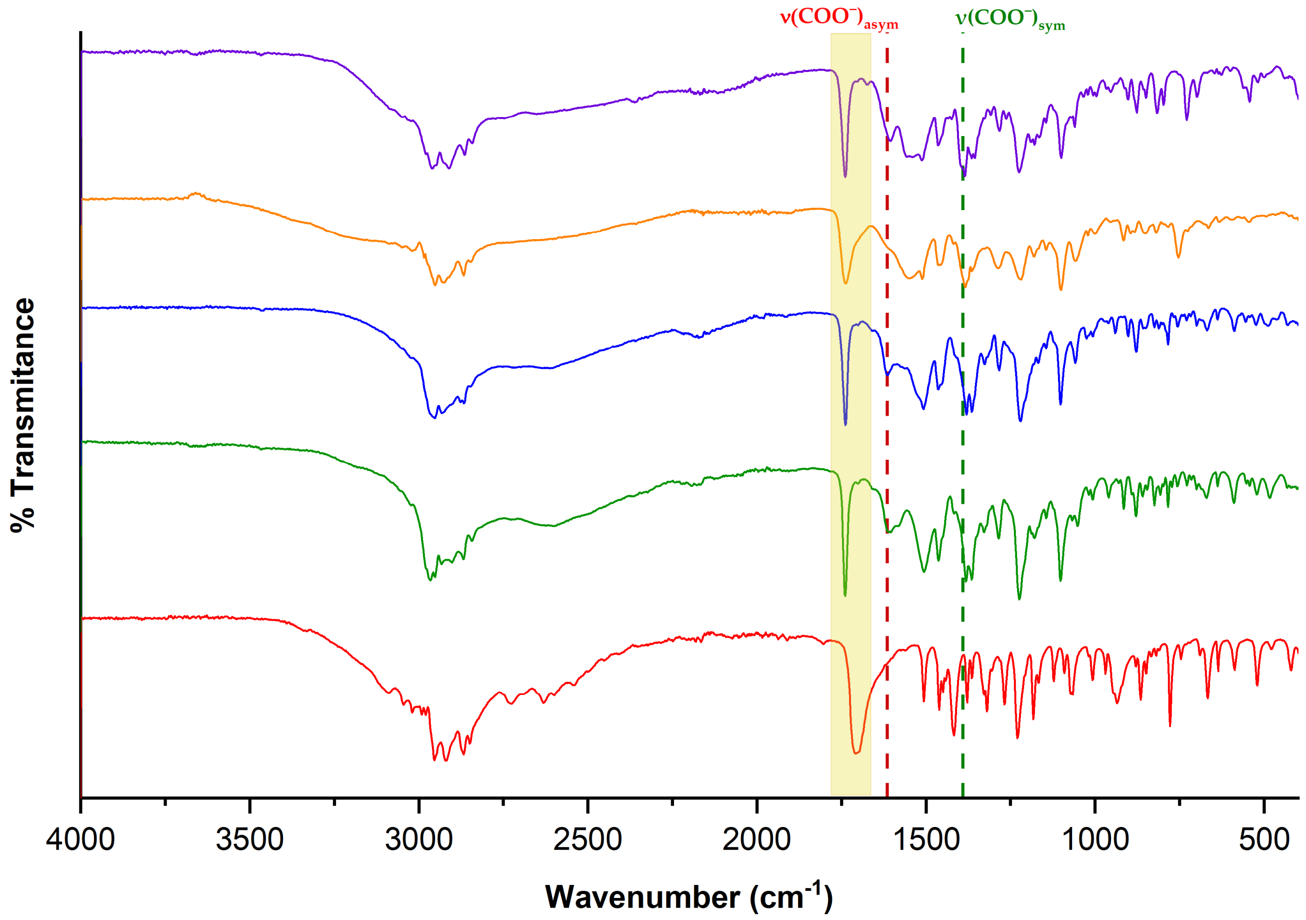

Figure S26. ATR-FTIR spectra for ibuprofen and its salts, from the bottom: unmodified acid (red), salts of [L-ValOiPr] (blue), salts of [L-IleOiPr] (green), salts of [L-ThrOiPr] (orange), salts of [L-MetOiPr][IBU] (purple), C=O stretching vibrations of the carboxylic acid group are marked in the yellow square, asymmetric ($\nu_{asym.}$) (COO^-), and symmetric ($\nu_{asym.}$) (COO^-) stretching vibrations marked as red and green dotted line, respectively.

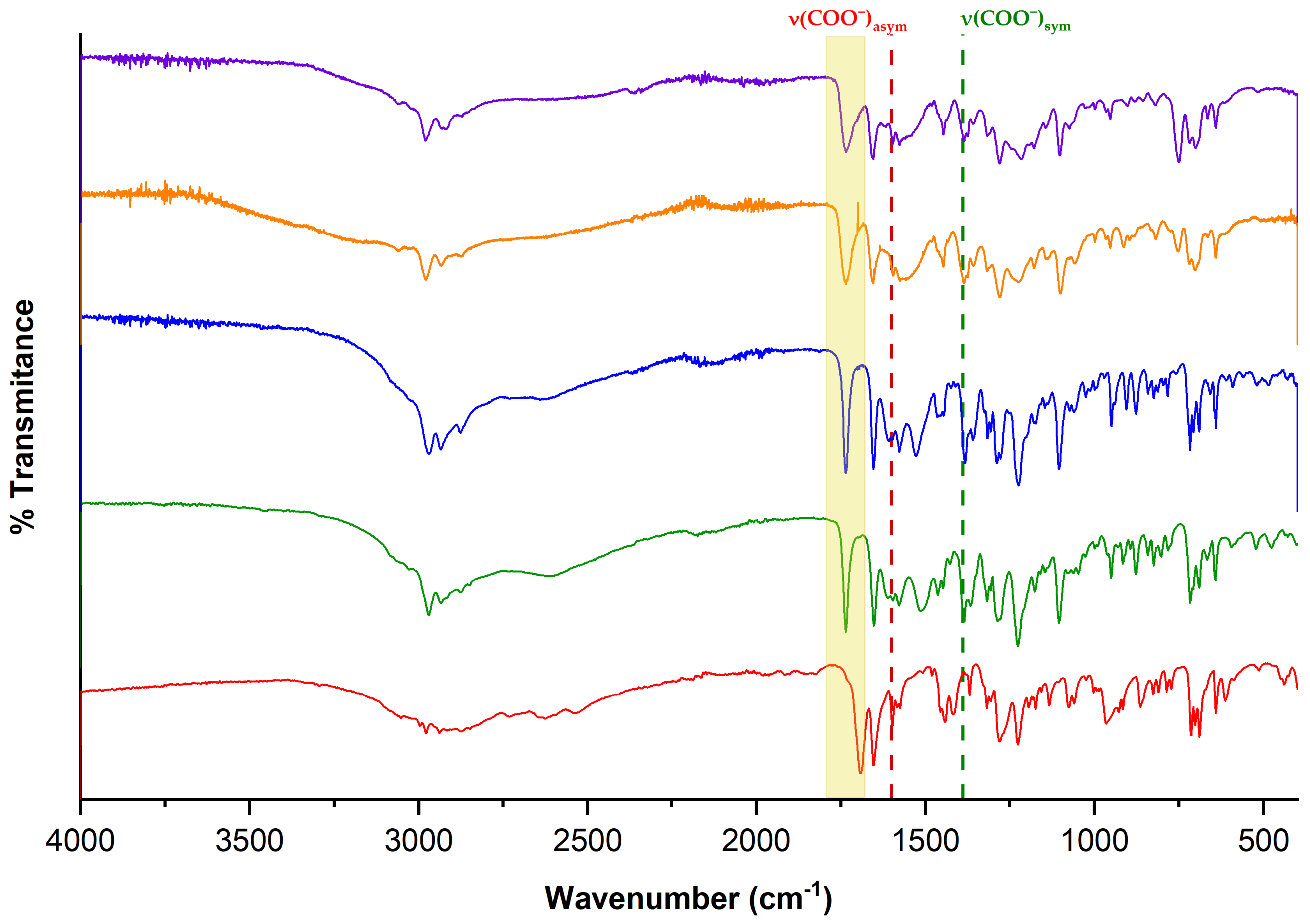

Figure S27. ATR-FTIR spectra for ketoprofen and its salts, from the bottom: unmodified acid (red), salts of [L-ValOiPr] (blue), salts of [L-IleOiPr] (green), salts of [L-ThrOiPr] (orange), salts of [L-MetOiPr][IBU] (purple), C=O stretching vibrations of the carboxylic acid group are marked in the yellow square, asymmetric ($\nu_{asym.}$) (COO^-), and symmetric ($\nu_{asym.}$) (COO^-) stretching vibrations marked as red and green dotted line, respectively.

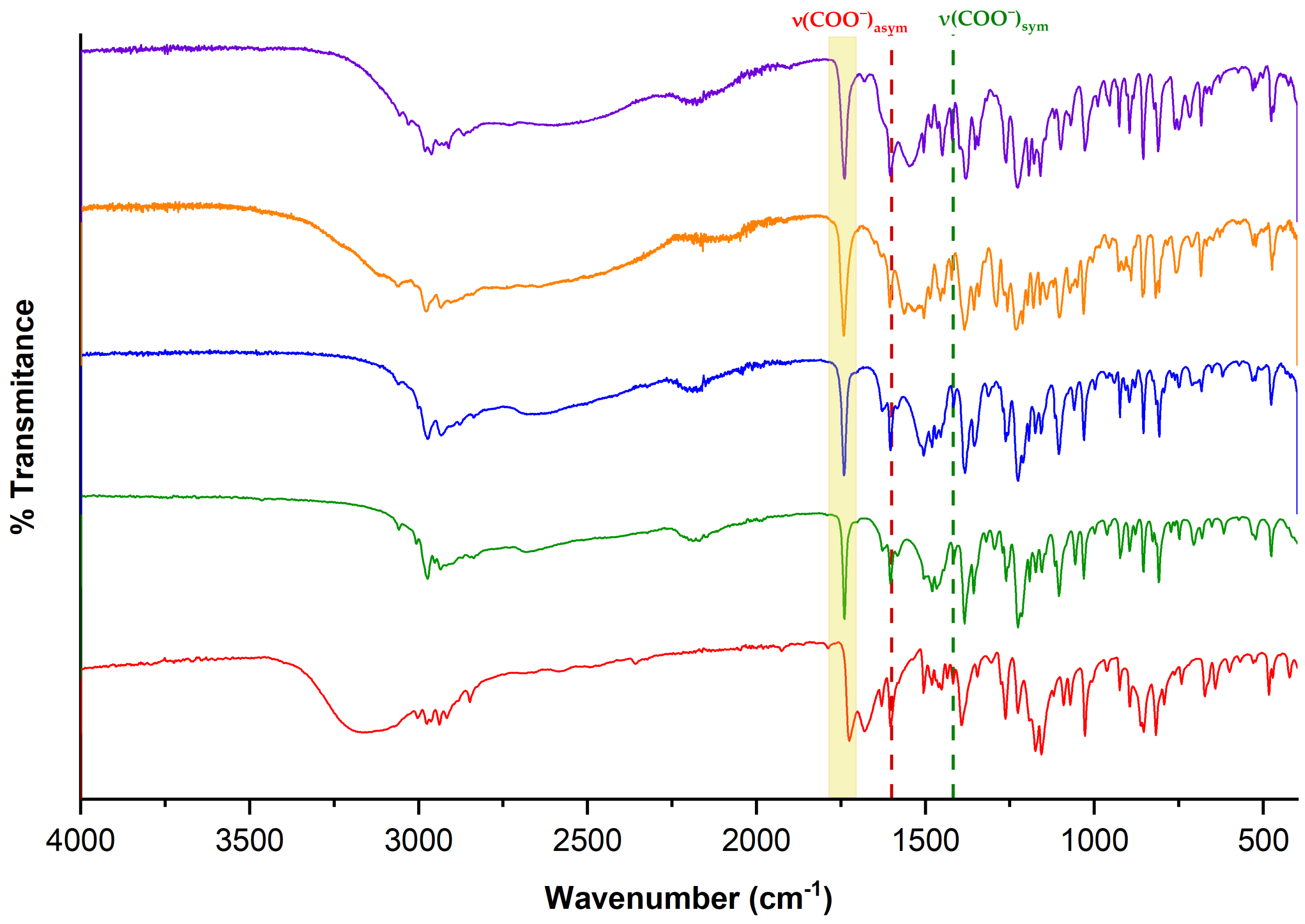

Figure S28. ATR-FTIR spectra for naproxen and its salts, from the bottom: unmodified acid (red), salts of [L-ValOiPr] (blue), salts of [L-IleOiPr] (green), salts of [L-ThrOiPr] (orange), salts of [L-MetOiPr][IBU] (purple), C=O stretching vibrations of the carboxylic acid group are marked in the yellow square, asymmetric ($\nu_{asym.}$) (COO^-), and symmetric ($\nu_{asym.}$) (COO^-) stretching vibrations marked as red and green dotted line, respectively.

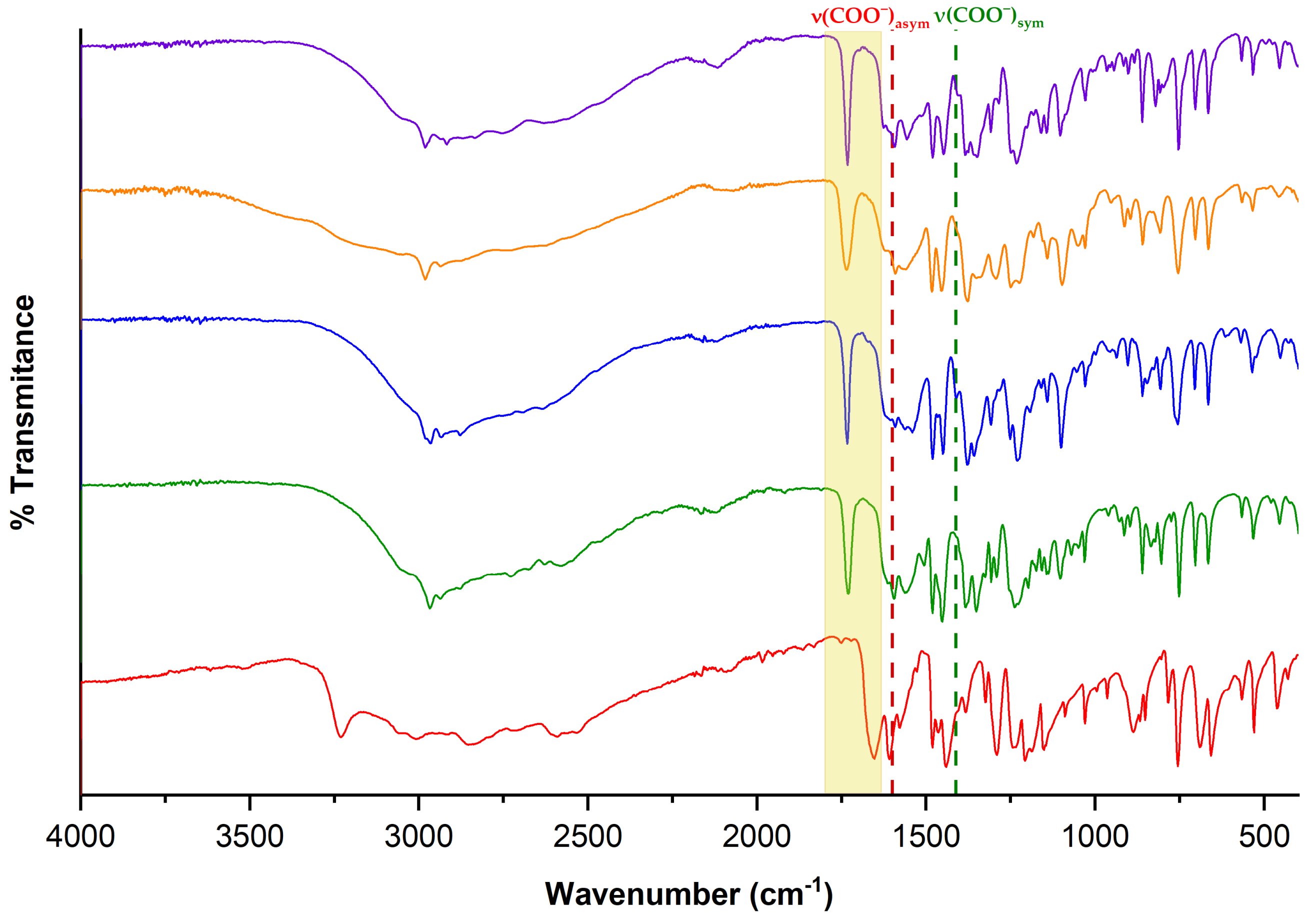

Figure S29. ATR-FTIR spectra for salicylic acid and its salts, from the bottom: unmodified acid (red), salts of [L-ValOiPr] (blue), salts of [L-IleOiPr] (green), salts of [L-ThrOiPr] (orange), salts of [L-MetOiPr][IBU] (purple), C=O stretching vibrations of the carboxylic acid group are marked in the yellow square, asymmetric ($\nu_{asym.}$) (COO^-), and symmetric ($\nu_{asym.}$) (COO^-) stretching vibrations marked as red and green dotted line, respectively.

The ATR-FTIR spectra of [AAOiPr][KETO]

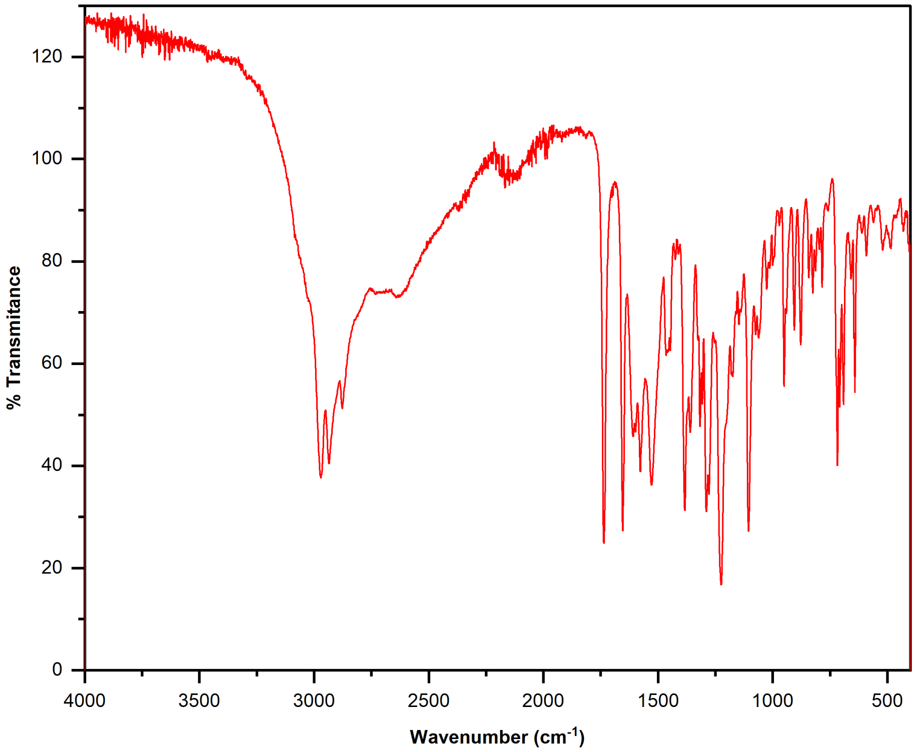

Figure S30. The ATR-FTIR spectrum of [L-IleOiPr][KETO].

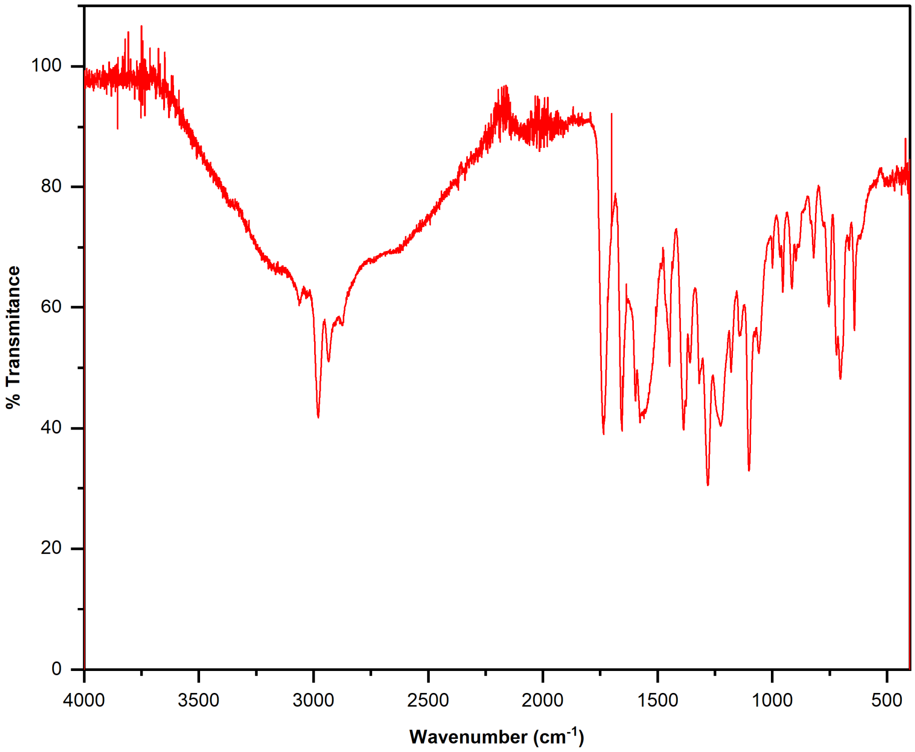

Figure S31. The ATR-FTIR spectrum of [L-ThrOiPr][KETO].

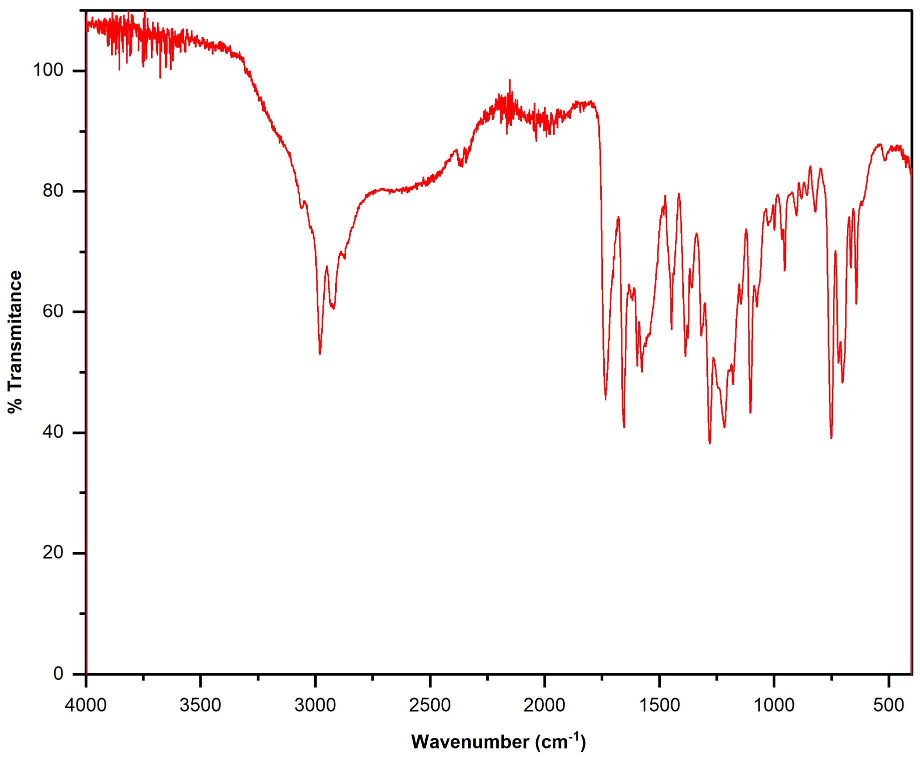

Figure S32. The ATR-FTIR spectrum of [L-MetOiPr][KETO].

The ATR-FTIR spectra of [AAOiPr][NAP]

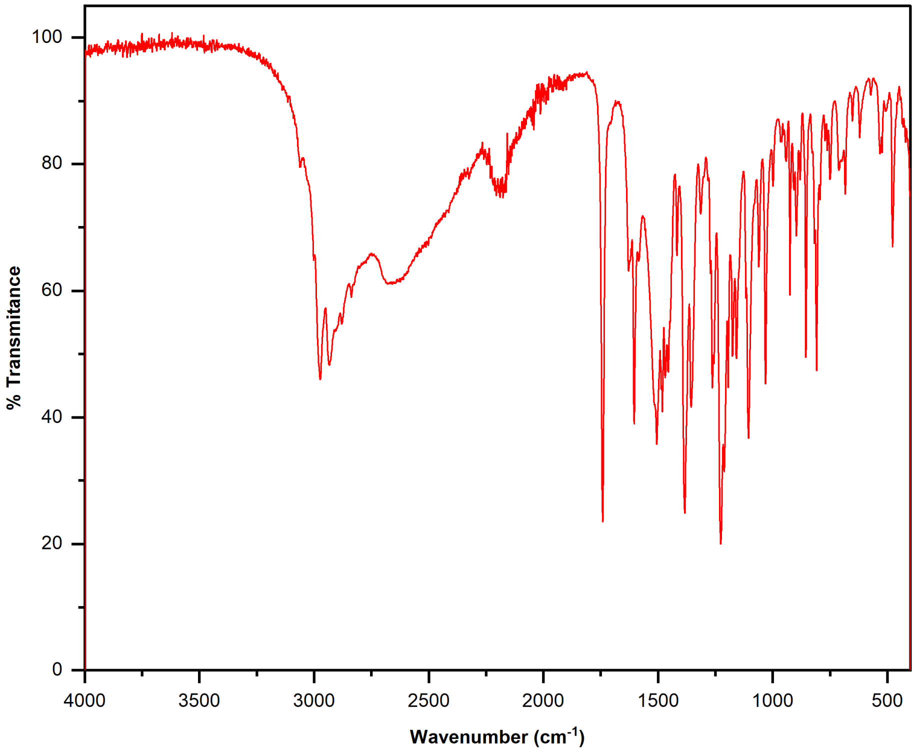

Figure S33. The ATR-FTIR spectrum of [L-IleOiPr][NAP].

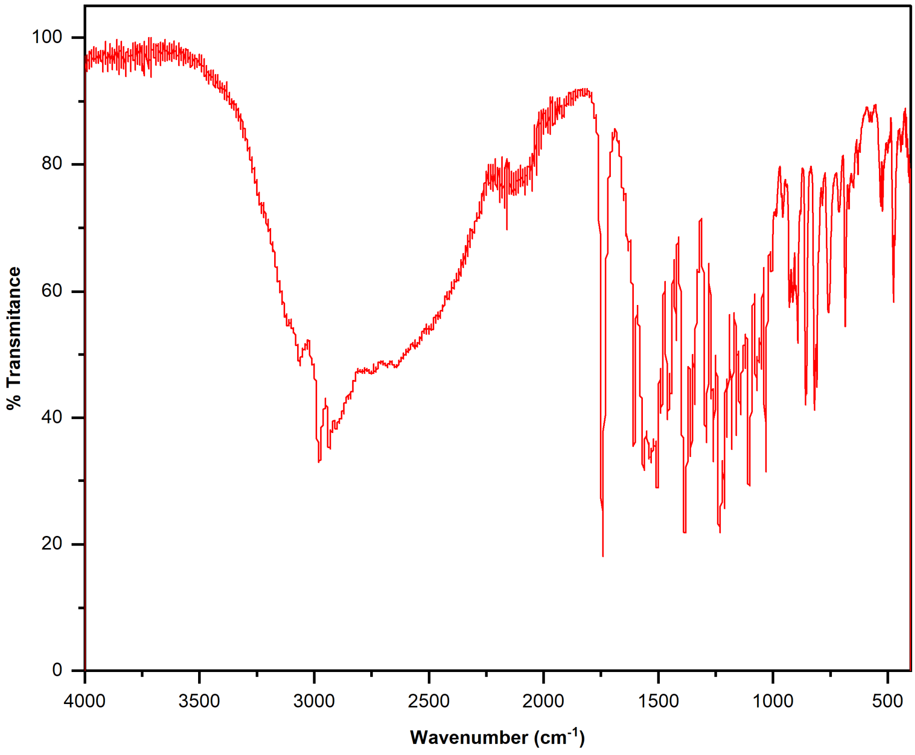

Figure S34. The ATR-FTIR spectrum of [L-ThrOiPr][NAP].

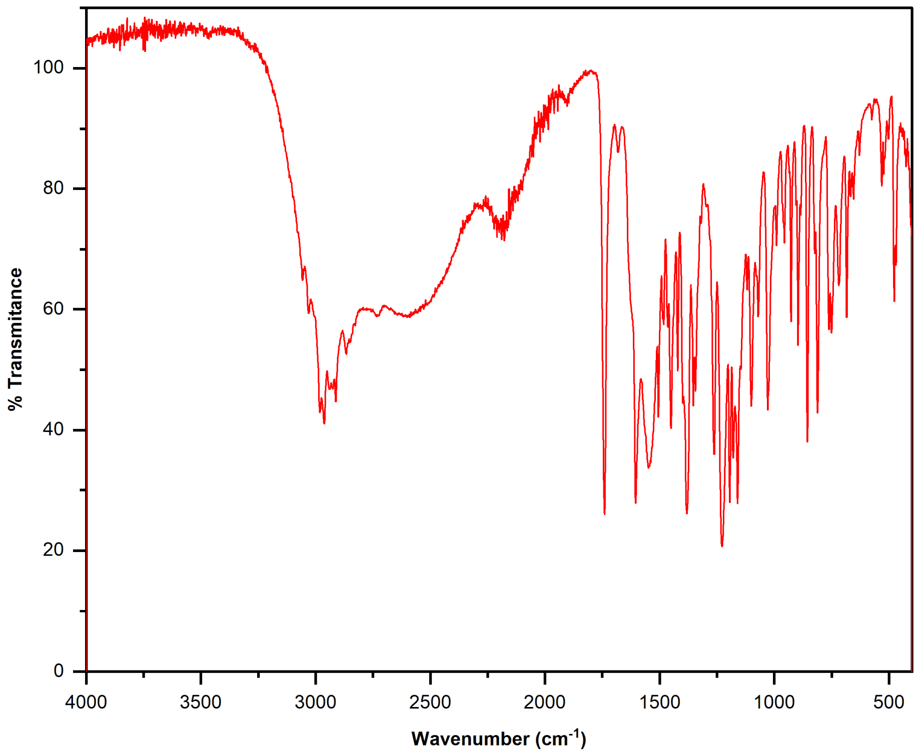

Figure S35. The ATR-FTIR spectrum of [L-MetOiPr][NAP].

The ATR-FTIR spectra of [AAOiPr][SA]

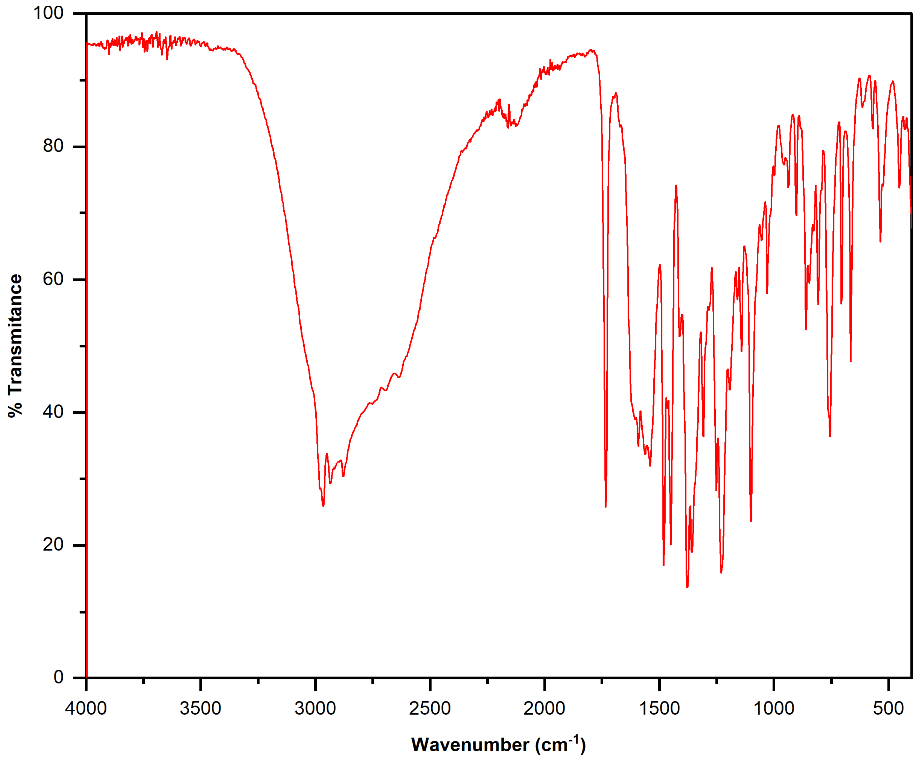

Figure S36. The ATR-FTIR spectrum of [L-IleOiPr][SA].

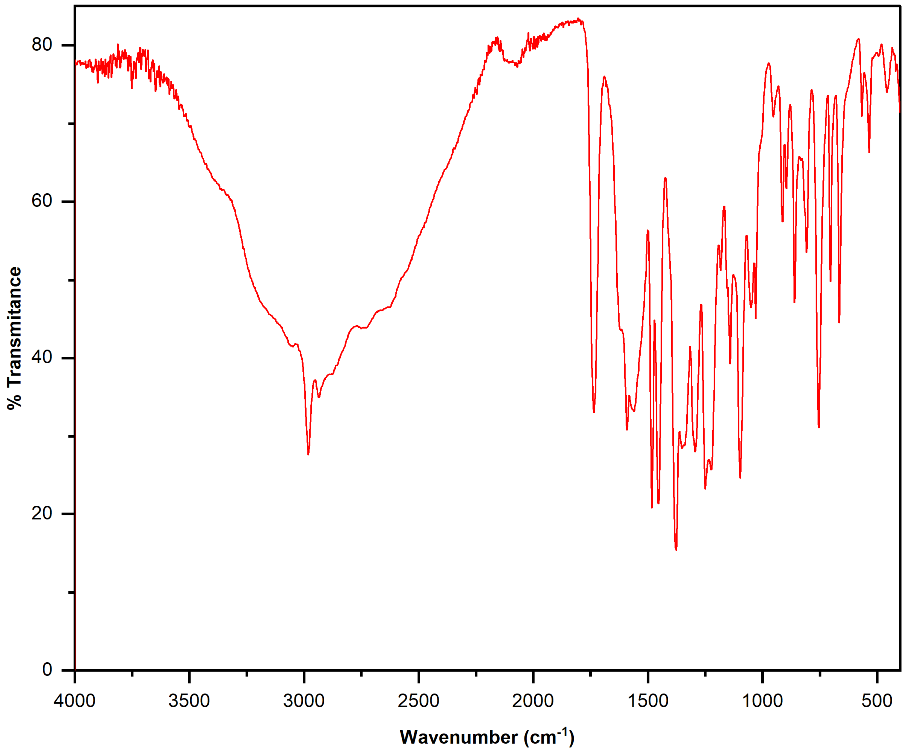

Figure S37. The ATR-FTIR spectrum of [L-ThrOiPr][SA].

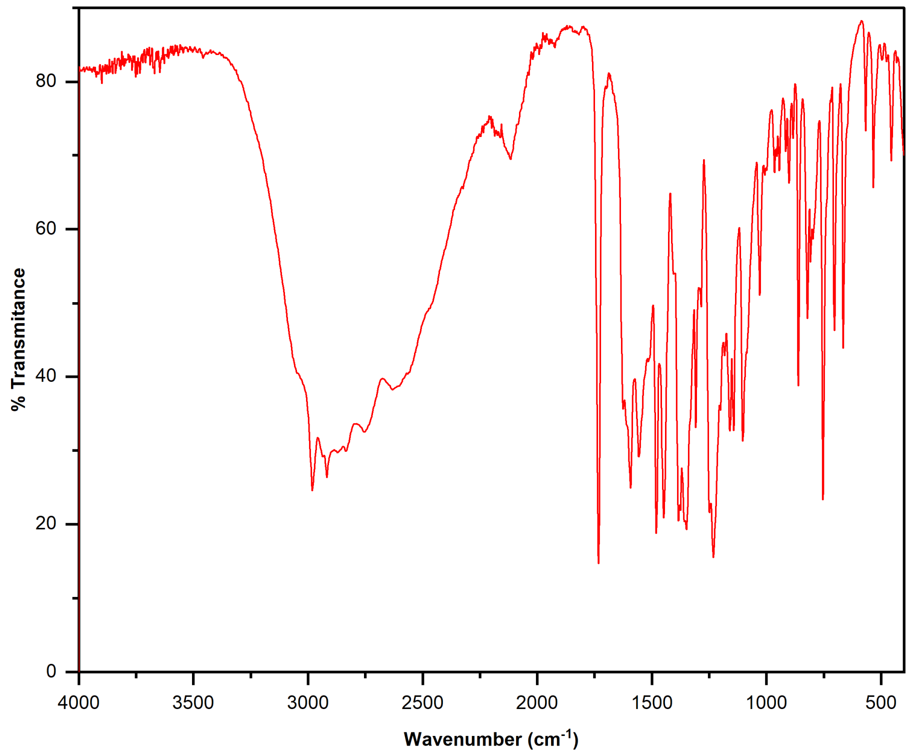

Figure S38. The ATR-FTIR spectrum of [L-MetOiPr][SA].

The UV–Vis spectra of [AAOiPr][KETO]

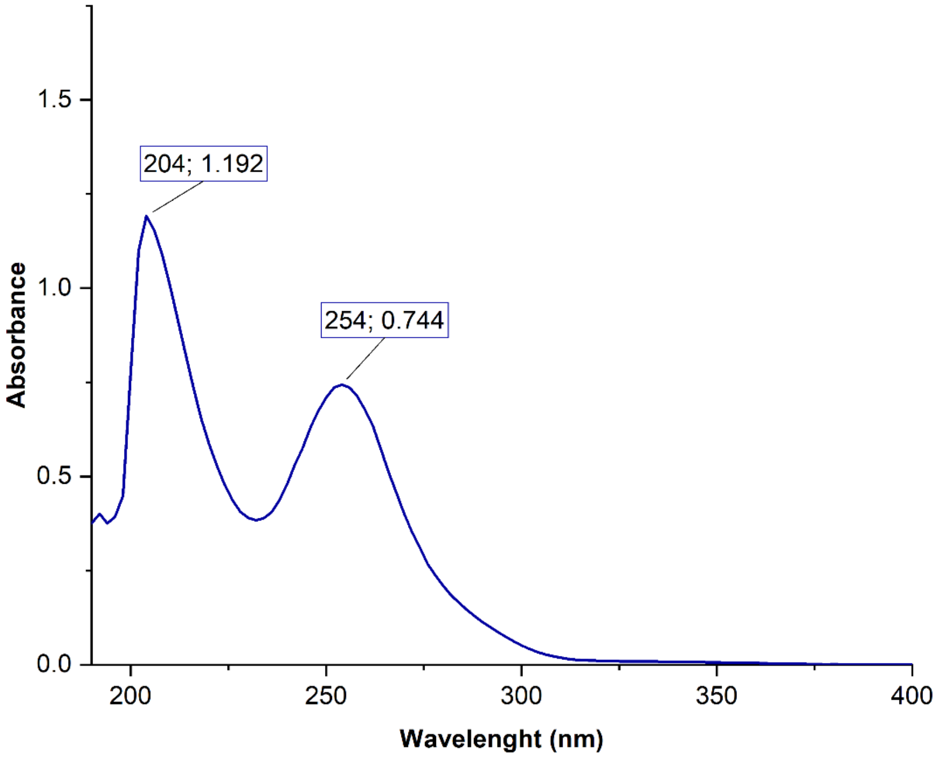

Figure S39. UV–Vis spectrum of [L-ValOiPr][KETO].

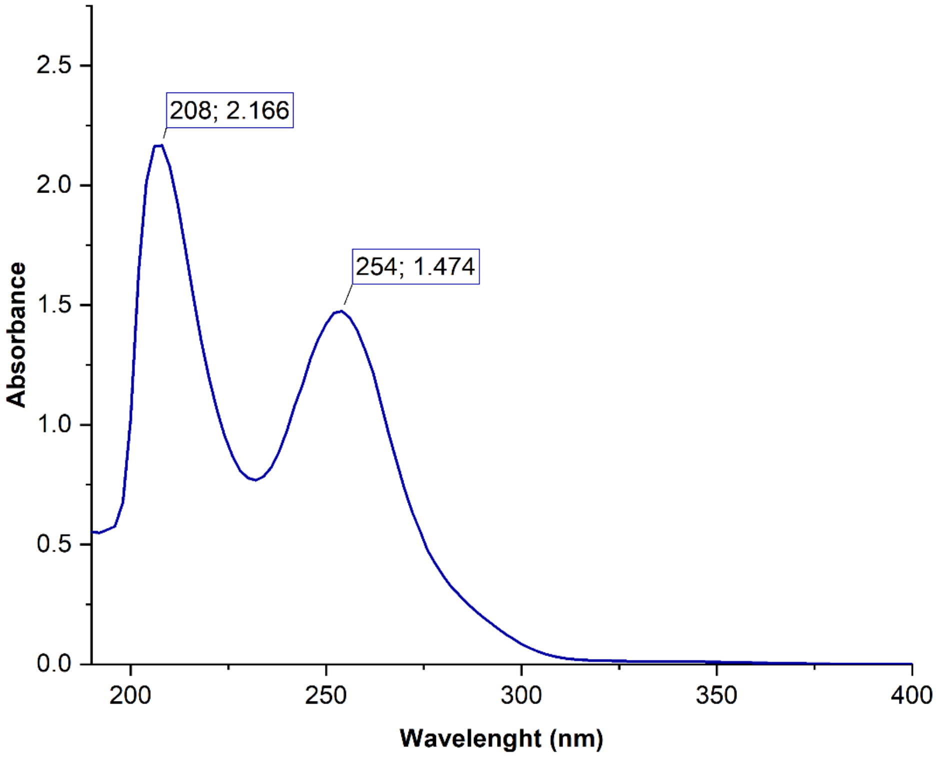

Figure S40. UV–Vis spectrum of [L-IleOiPr][KETO].

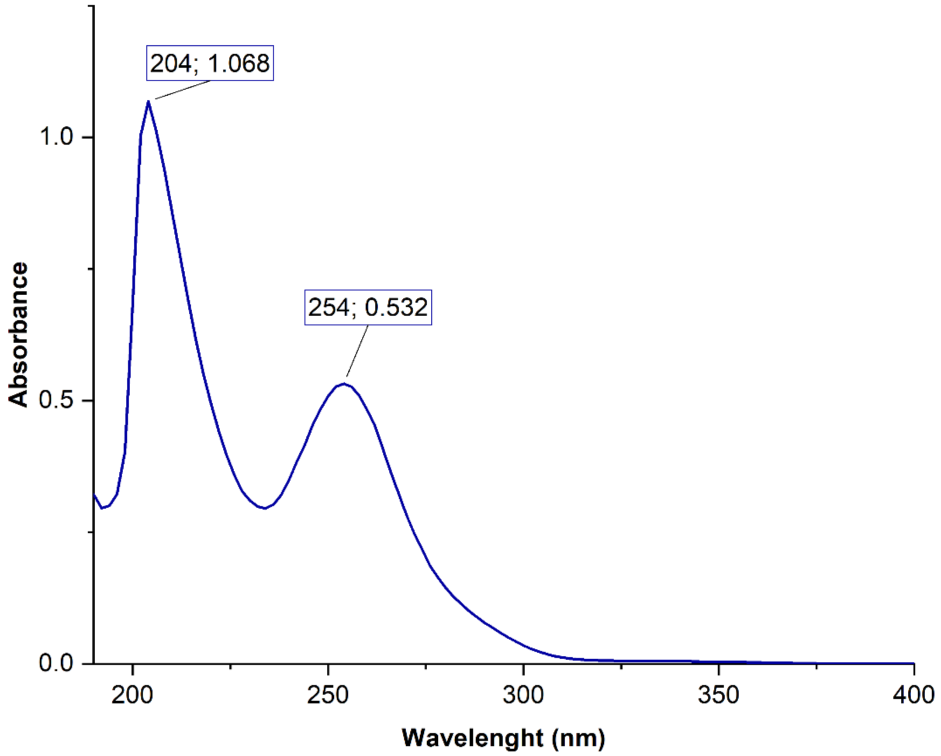

Figure S41. UV–Vis spectrum of [L-ThrOiPr][KETO].

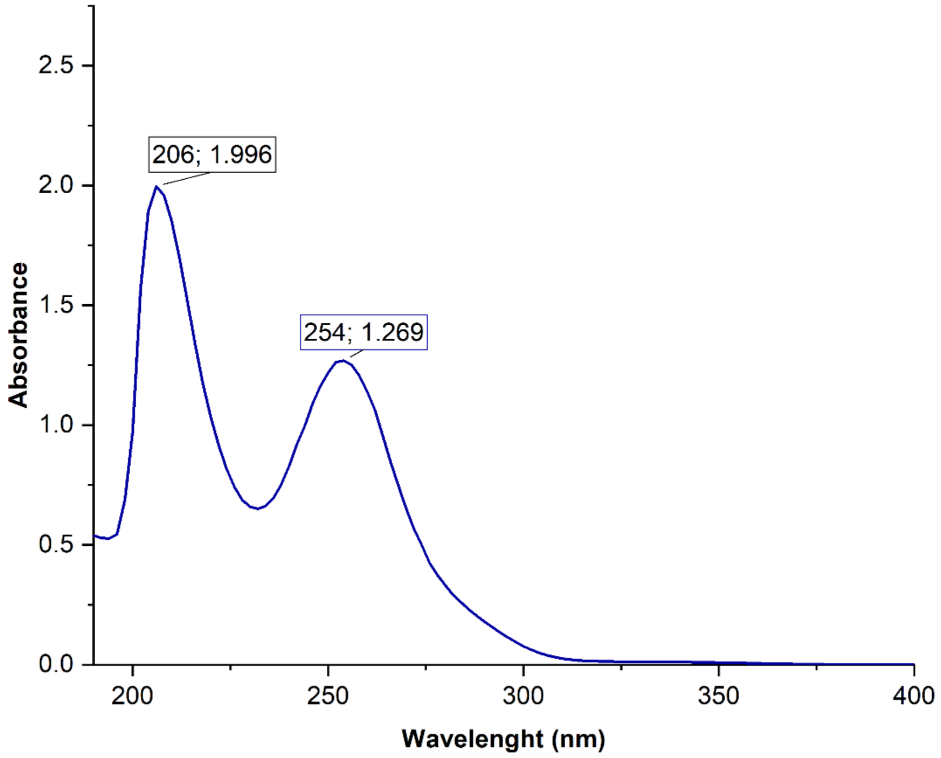

Figure S42. UV–Vis spectrum of [L-MetOiPr][KETO].

The UV–Vis spectra of [AAOiPr][NAP]

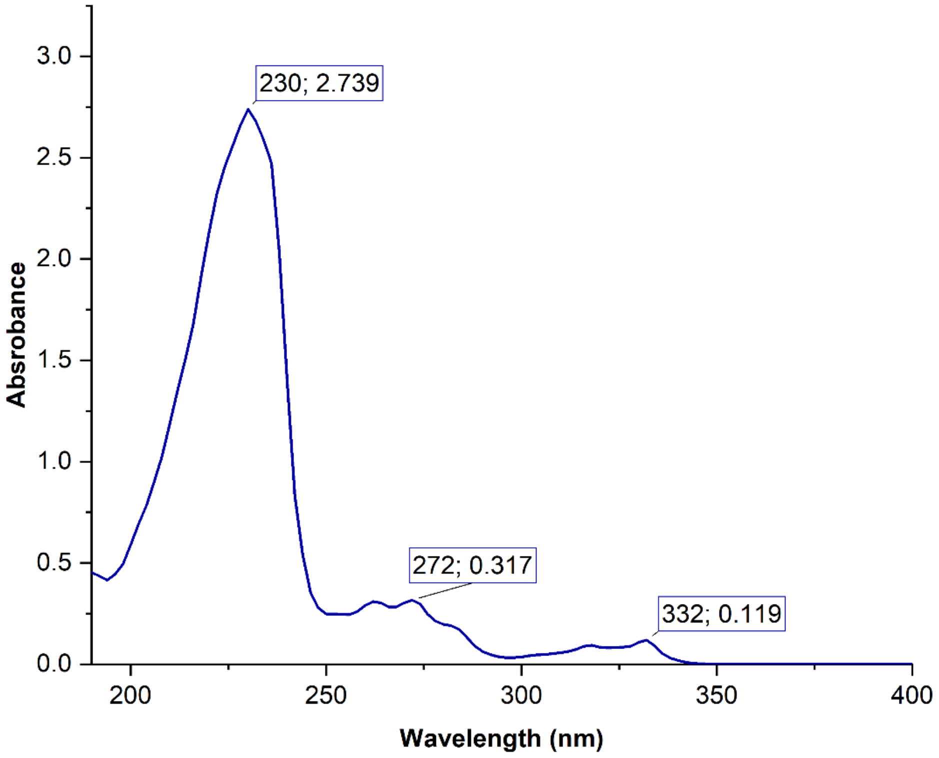

Figure S43. UV–Vis spectrum of [L-ValOiPr][NAP].

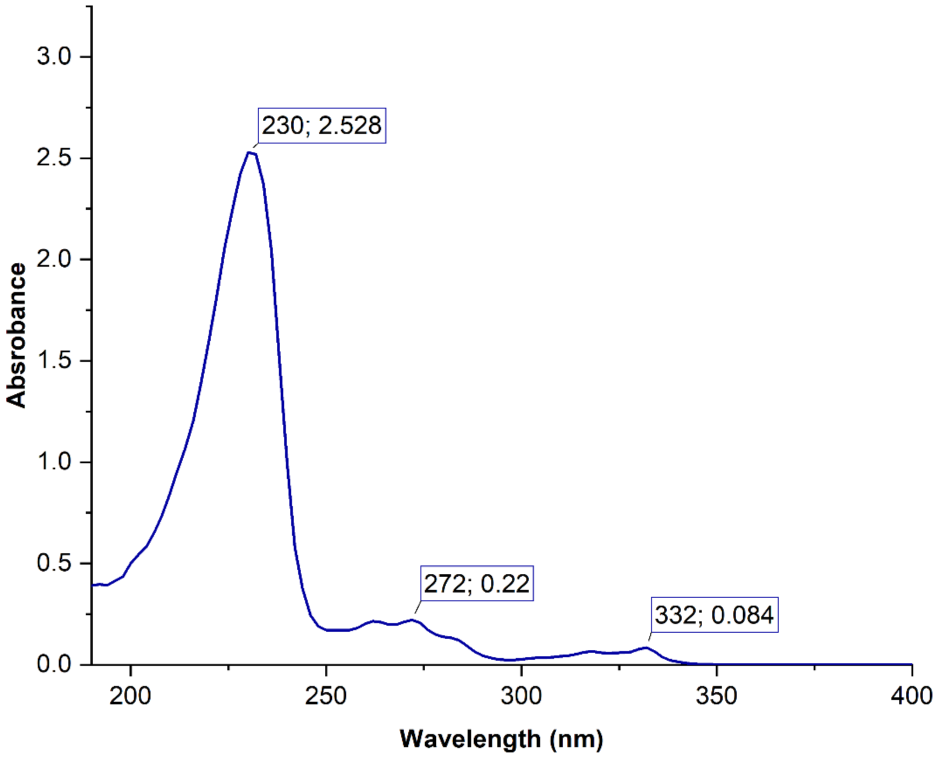

Figure S44. UV–Vis spectrum of [L-IleOiPr][NAP].

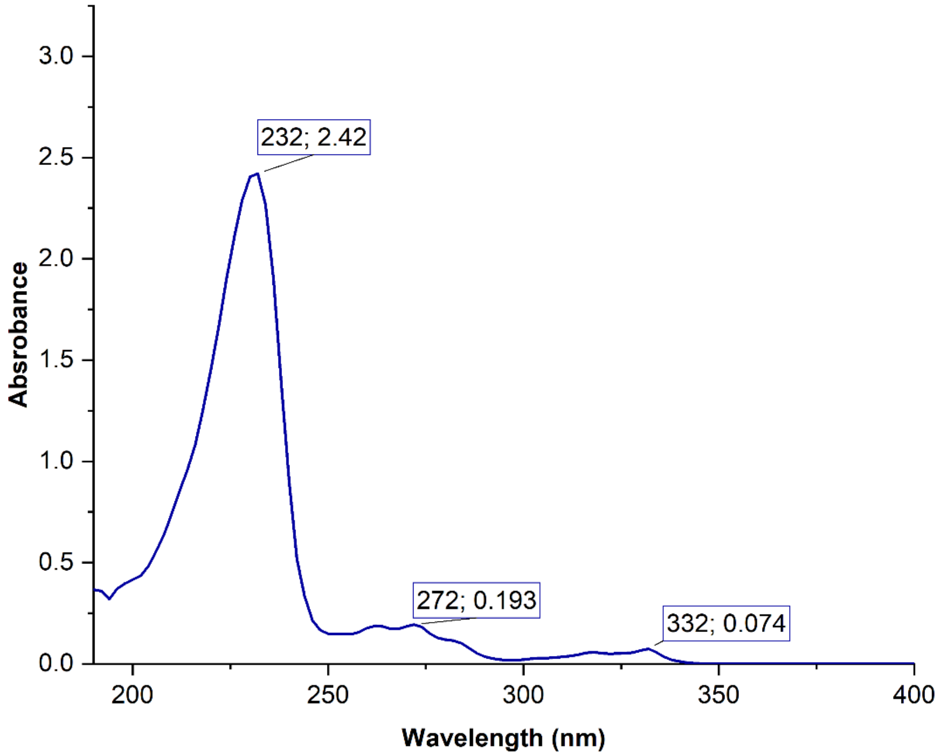

Figure S45. UV–Vis spectrum of [L-ThrOiPr][NAP].

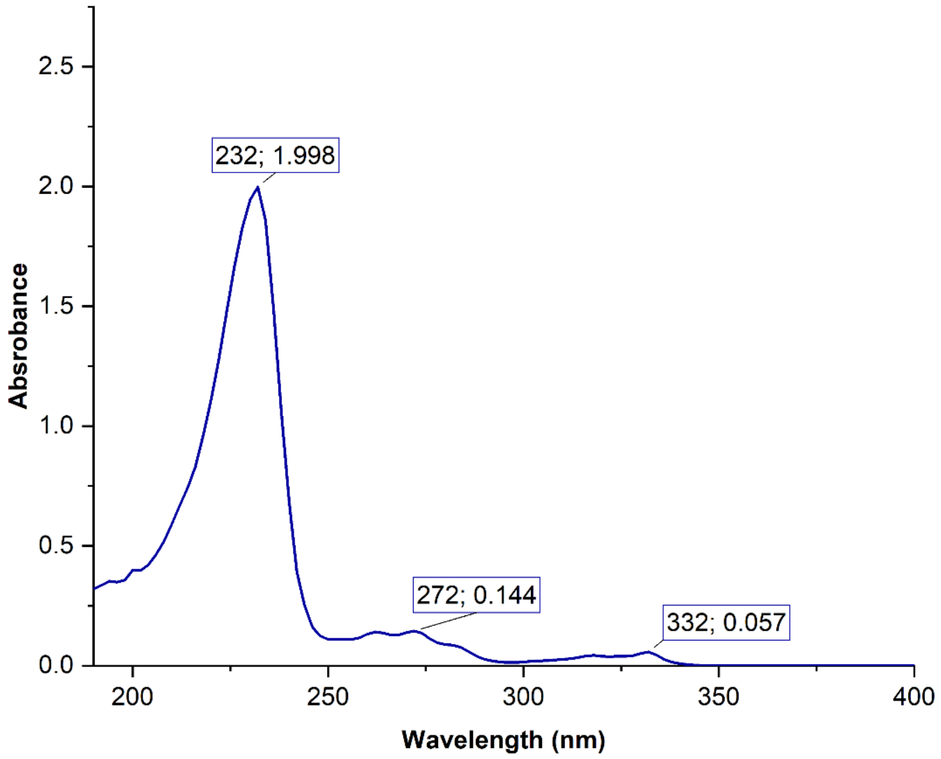

Figure S46. UV–Vis spectrum of [L-MetOiPr][NAP].

The UV–Vis spectra of [AAOiPr][SA]

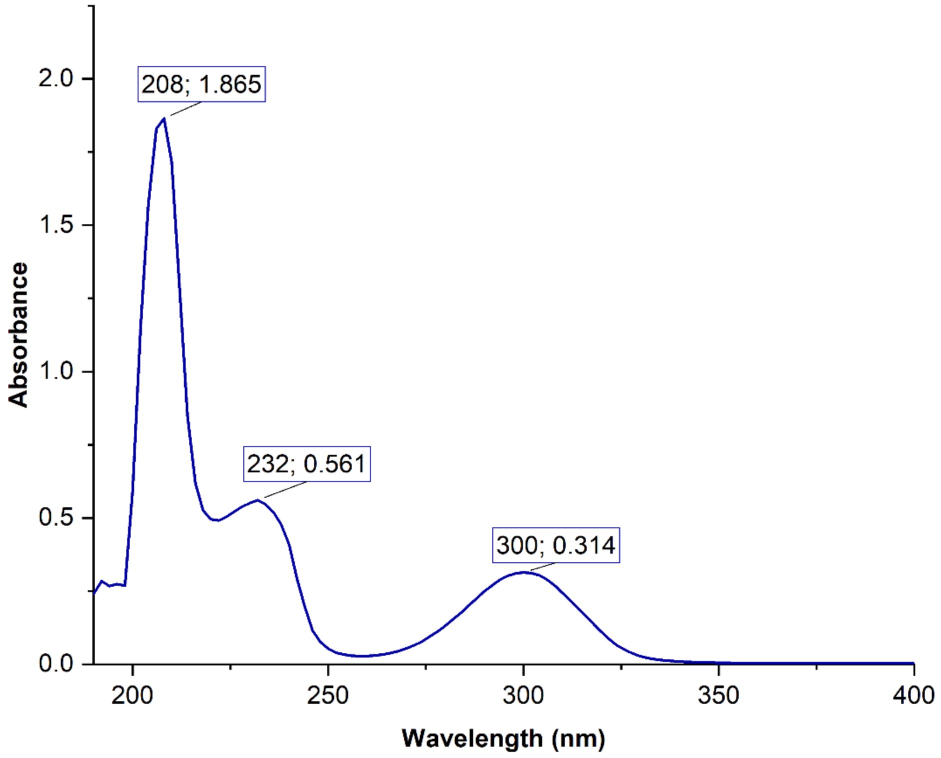

Figure S47. UV–Vis spectrum of [L-ValOiPr][SA].

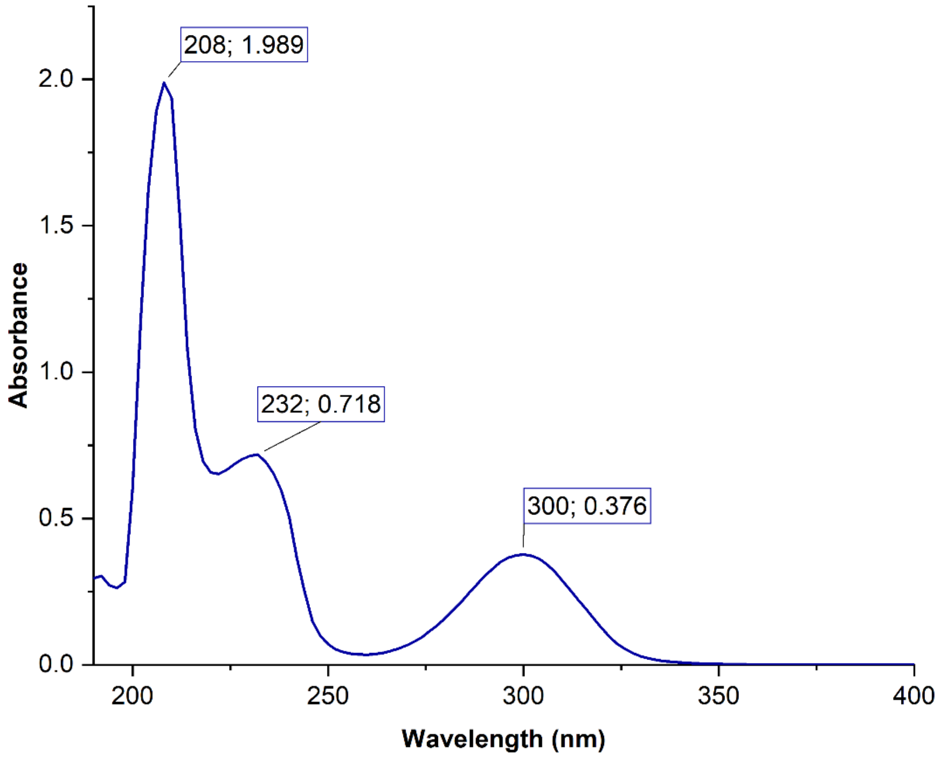

Figure S48. UV–Vis spectrum of [L-IleOiPr][SA].

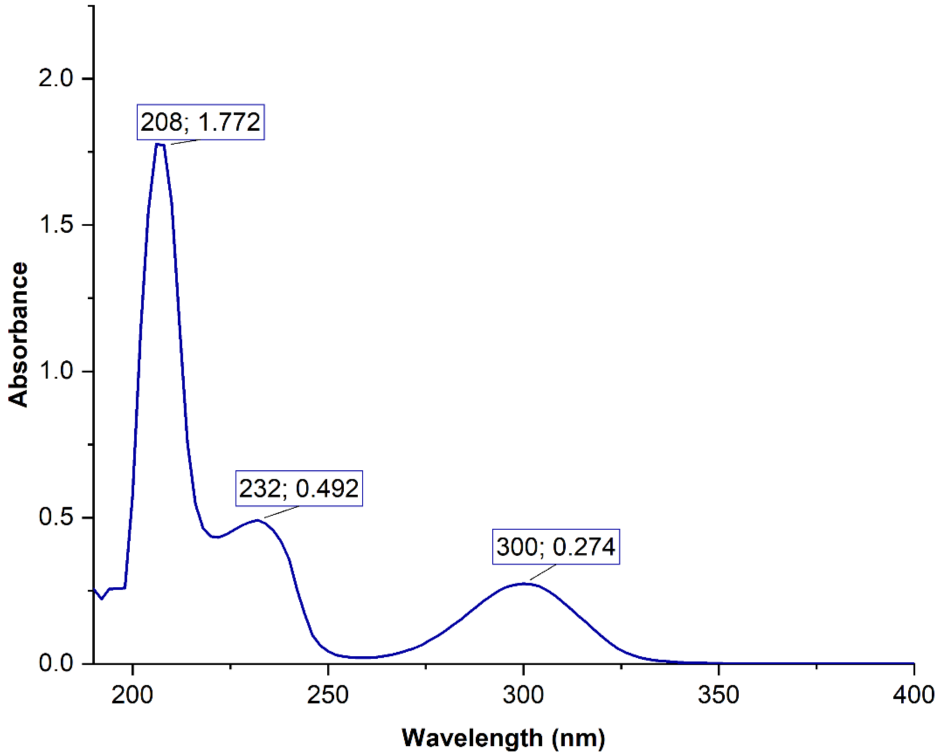

Figure S49. UV–Vis spectrum of [L-ThrOiPr][SA].

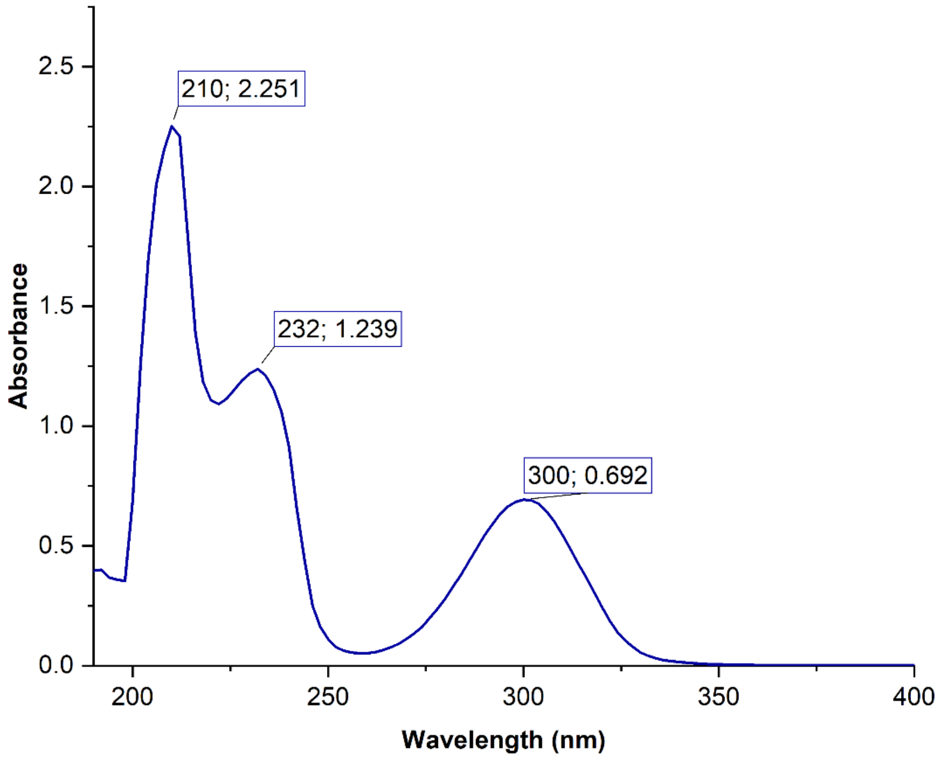

Figure S50. UV–Vis spectrum of [L-MetOiPr][SA].

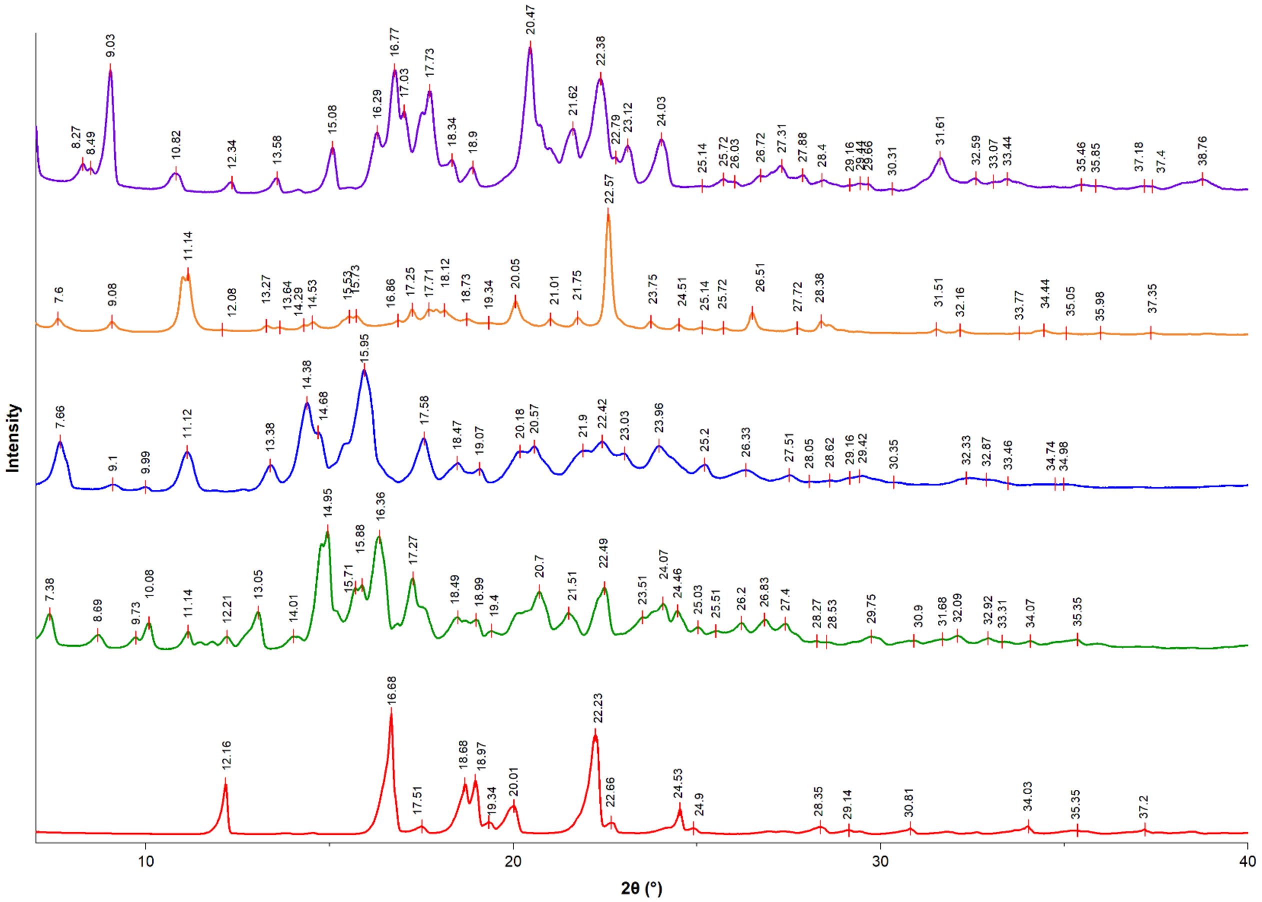

Figure S51. X-ray diffraction patterns of ibuprofen and its salts, from the bottom: unmodified acid (red), salts of [L-ValOiPr] (blue), salts of [L-IleOiPr] (green), salts of [L-ThrOiPr] (orange), salts of [L-MetOiPr][IBU] (purple).

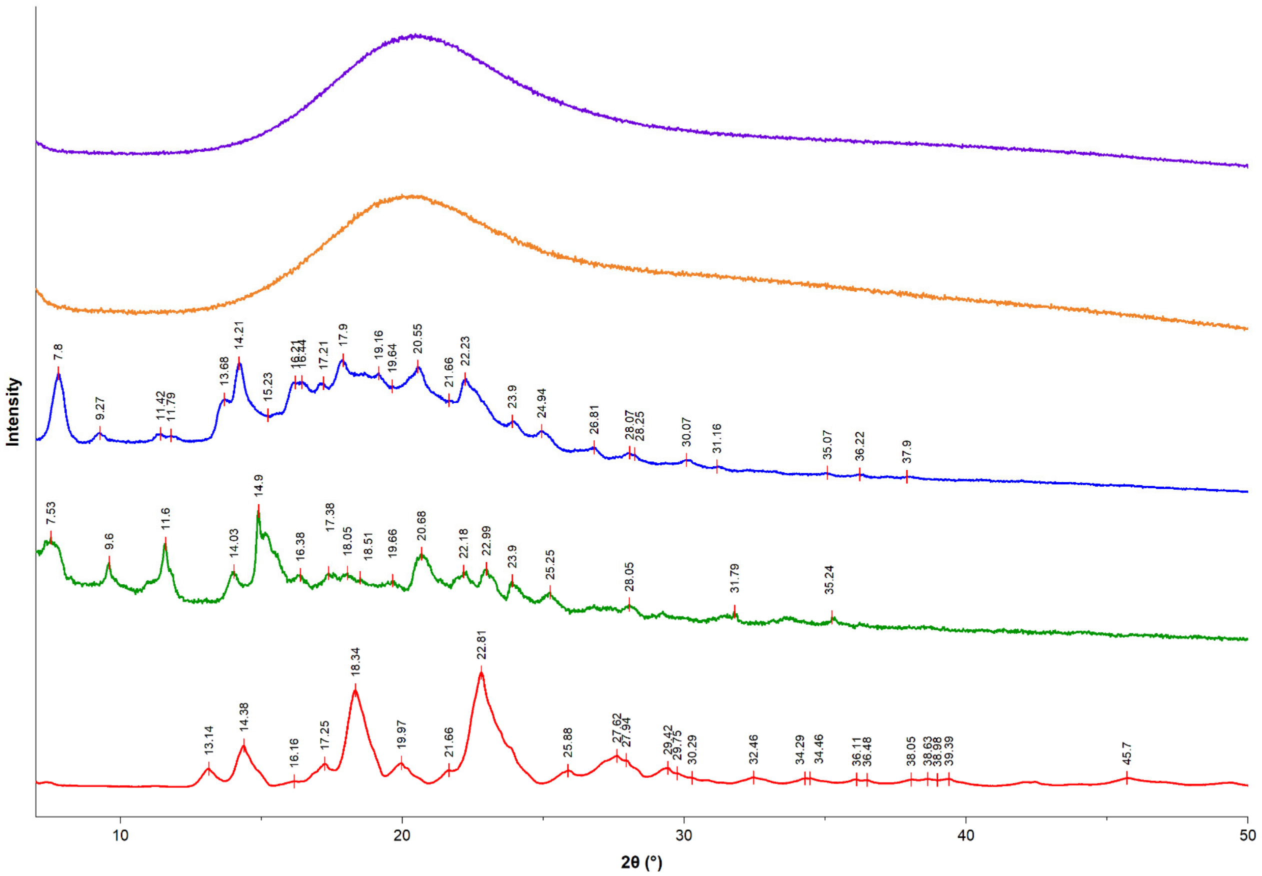

Figure S52. X-ray diffraction patterns of ketoprofen and its salts, from the bottom: unmodified acid (red), salts of [L-ValOiPr] (blue), salts of [L-IleOiPr] (green), salts of [L-ThrOiPr] (orange), salts of [L-MetOiPr][IBU] (purple).

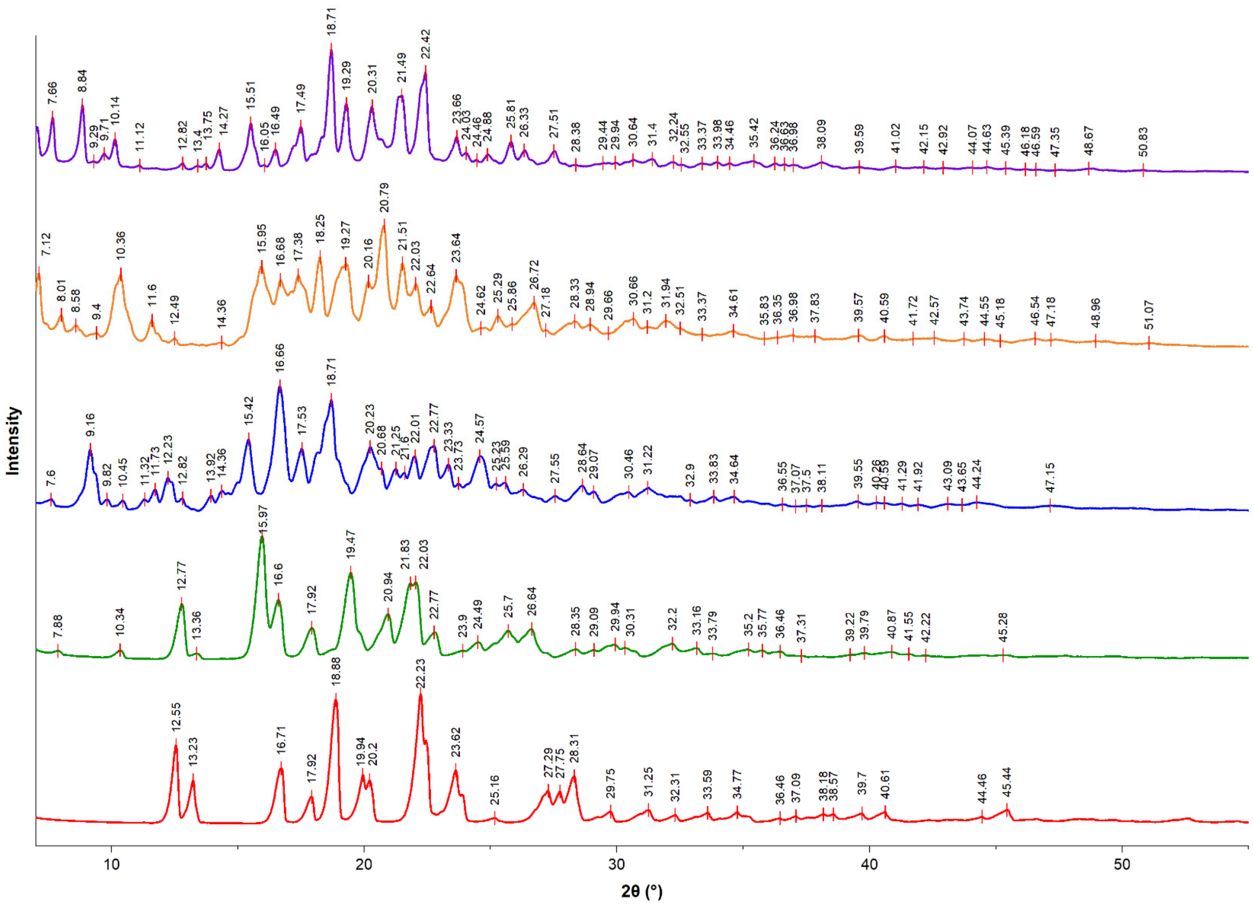

Figure S53. X-ray diffraction patterns of naproxen and its salts, from the bottom: unmodified acid (red), salts of [L-ValOiPr] (blue), salts of [L-IleOiPr] (green), salts of [L-ThrOiPr] (orange), salts of [L-MetOiPr][IBU] (purple).

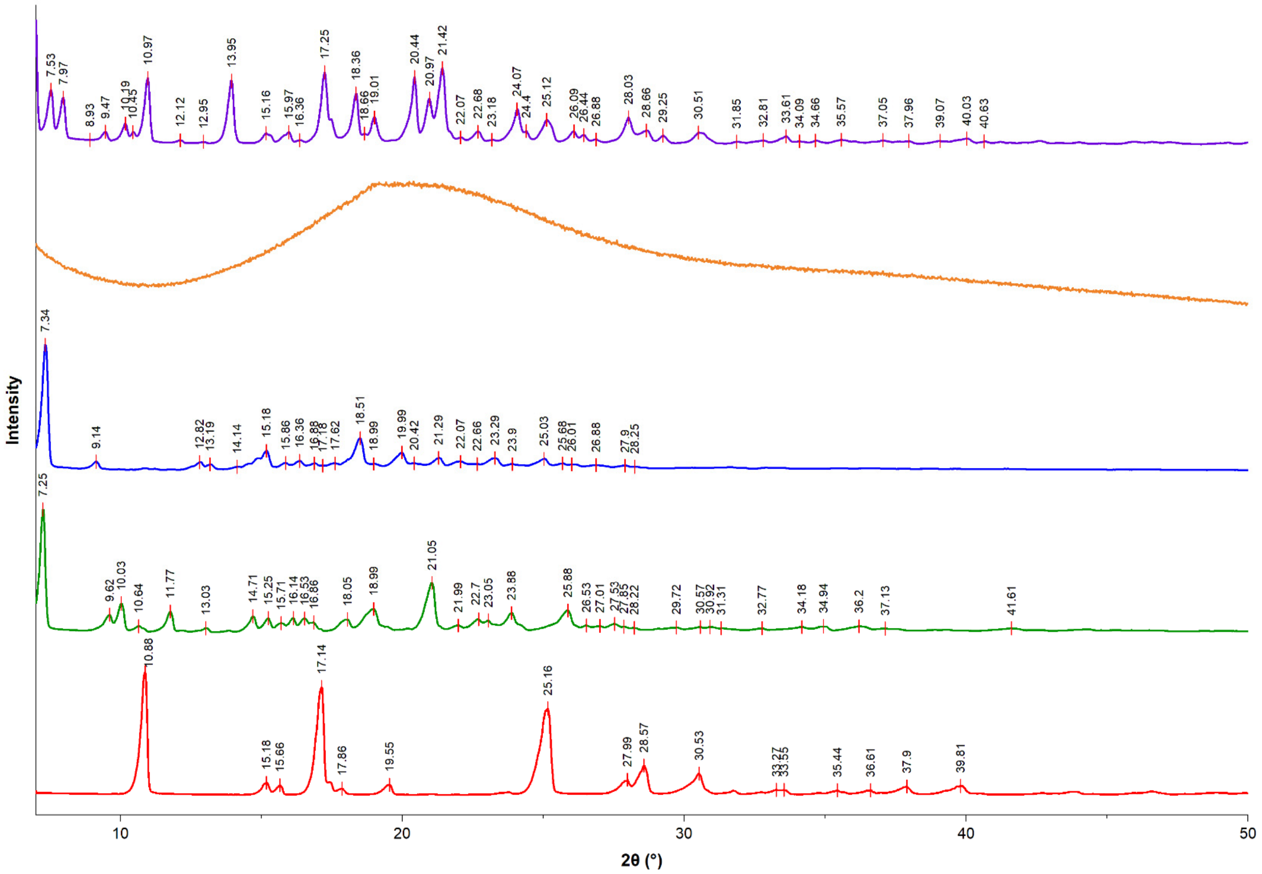

Figure S54. X-ray diffraction patterns of naproxen and its salts, from the bottom: unmodified acid (red), salts of [L-ValOiPr] (blue), salts of [L-IleOiPr] (green), salts of [L-ThrOiPr] (orange), salts of [L-MetOiPr][IBU] (purple).

X-ray diffraction (XRD) patterns of [AAOiPr][KETO]

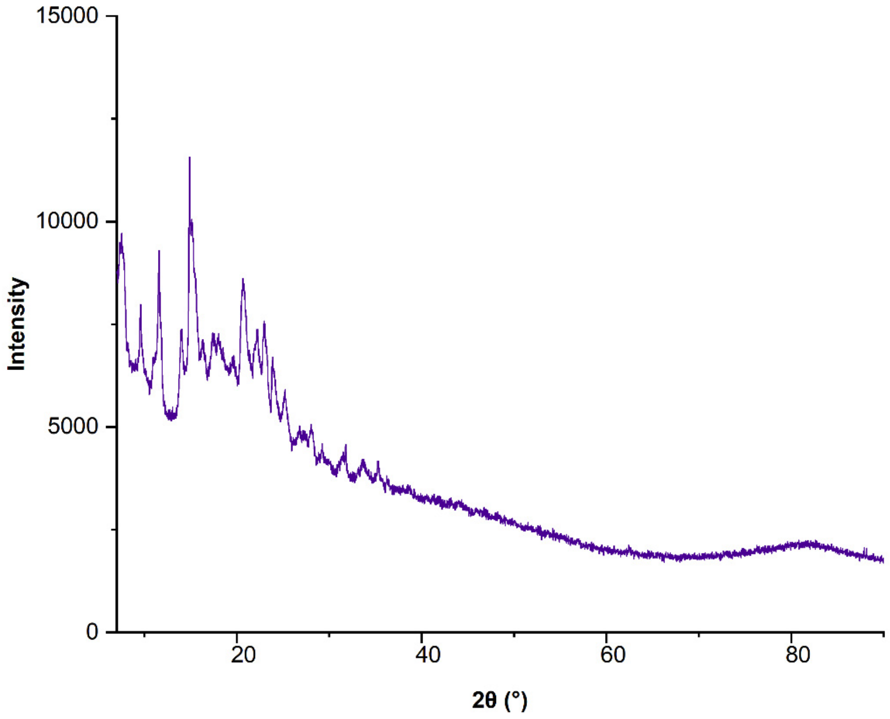

Figure S55. XRD pattern of [L-ValOiPr][KETO].

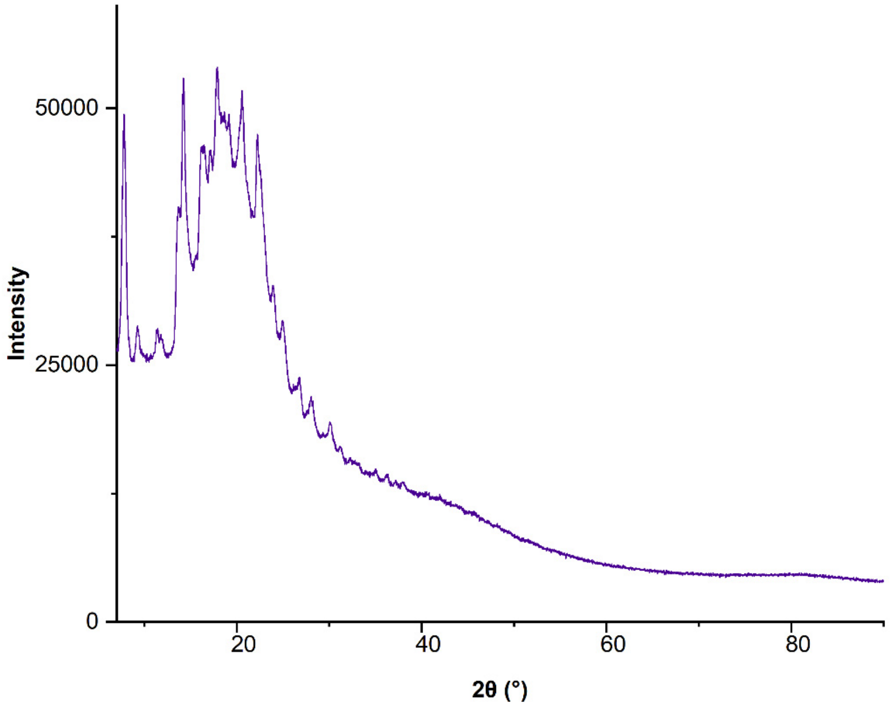

Figure S56. XRD pattern of [L-IleOiPr][KETO].

X-ray diffraction (XRD) patterns of [AAOiPr][NAP]

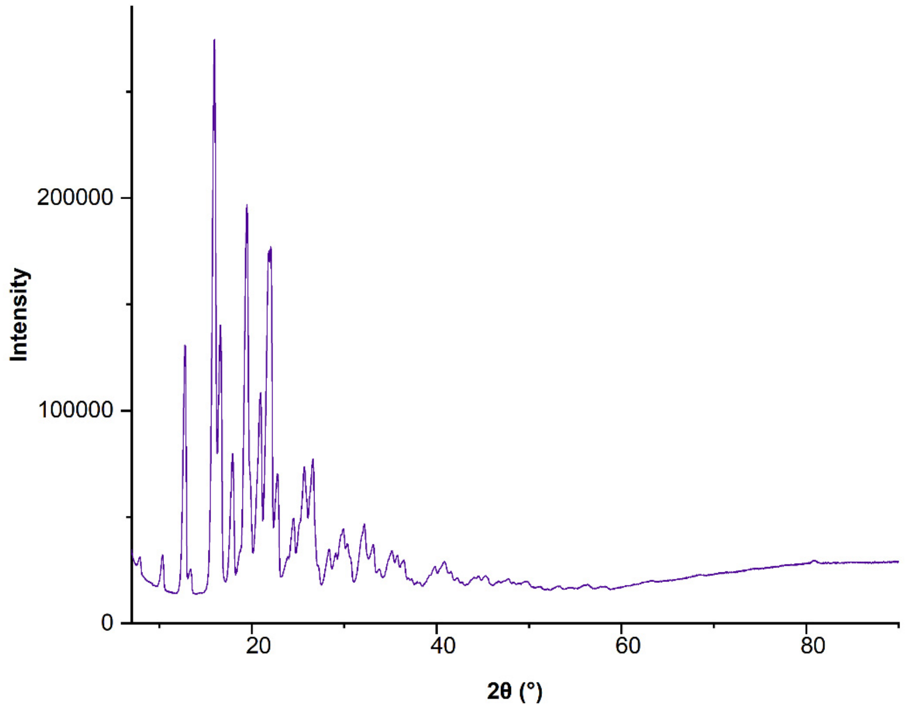

Figure S57. XRD pattern of [L-ValOiPr][NAP].

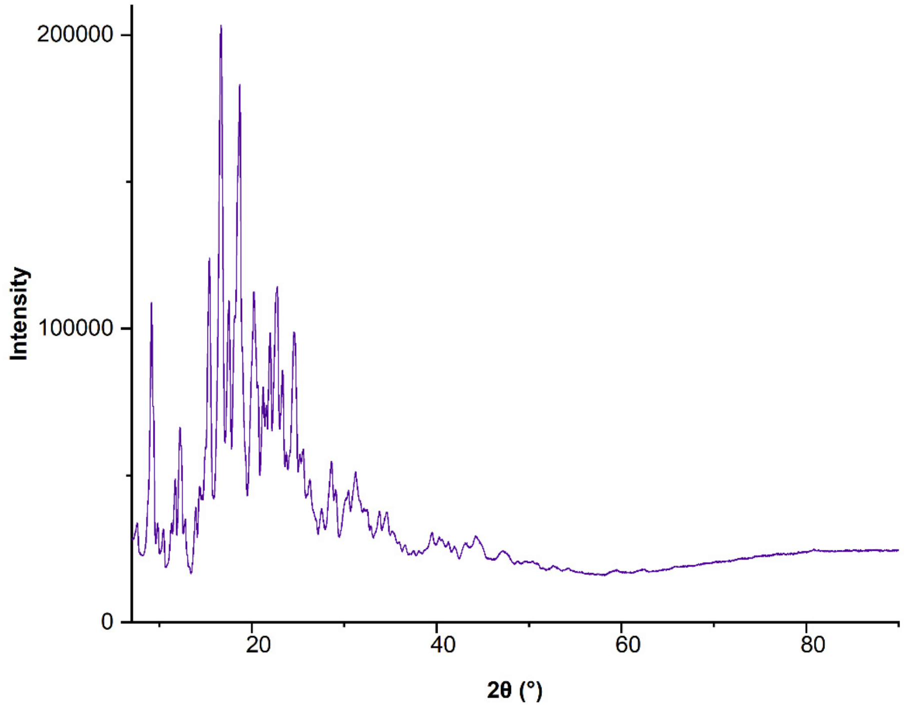

Figure S58. XRD pattern of [L-IleOiPr][NAP].

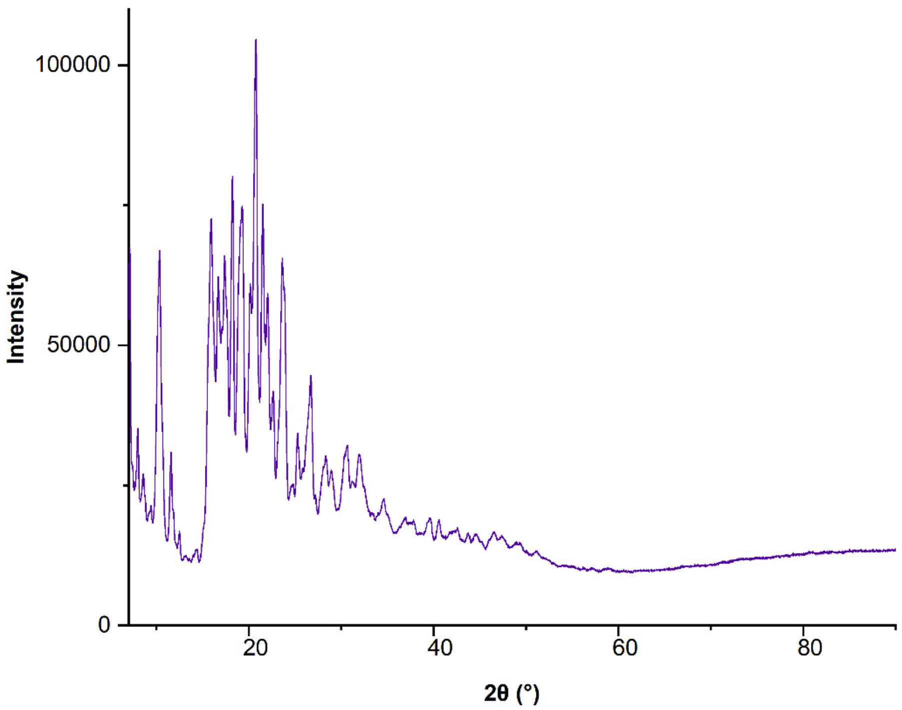

Figure S59. XRD pattern of [L-ThrOiPr][NAP].

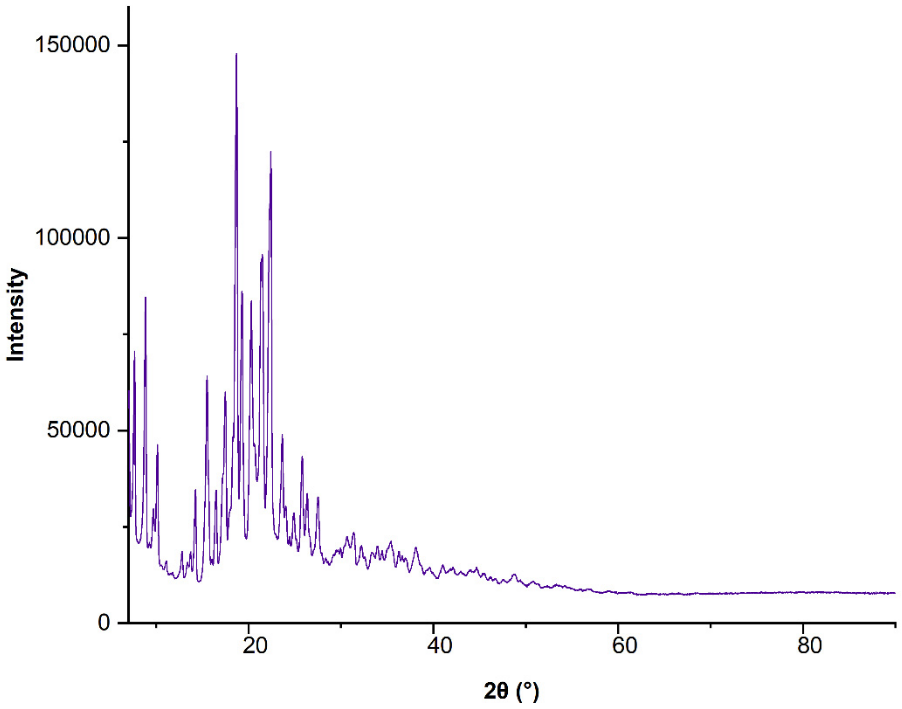

Figure S60. XRD pattern of [L-MetOiPr][NAP].

X-ray diffraction (XRD) patterns of [AAOiPr][SA]

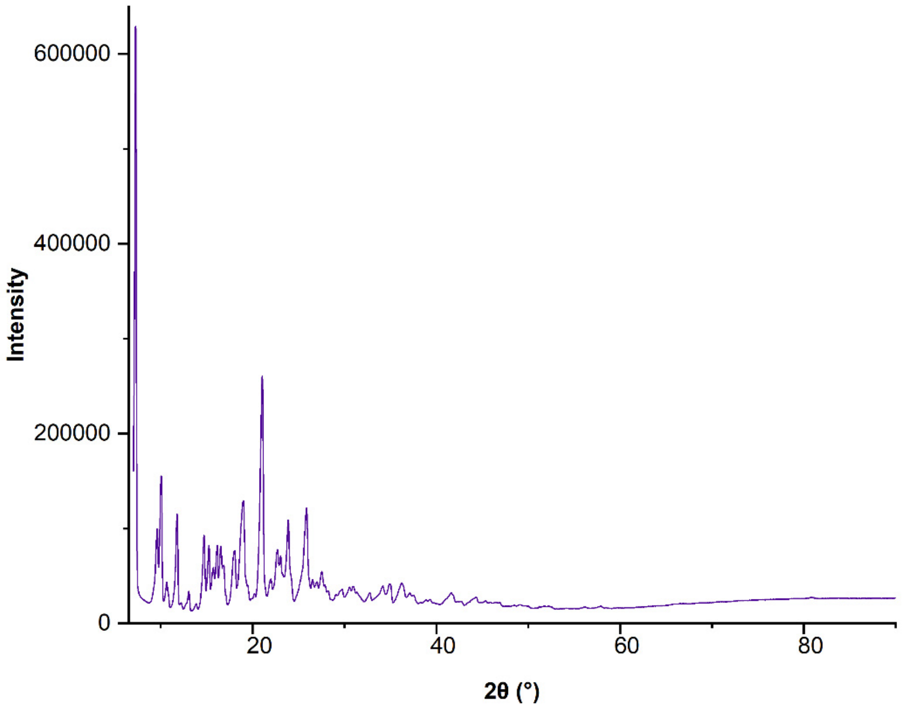

Figure S61. XRD pattern of [L-ValOiPr][SA].

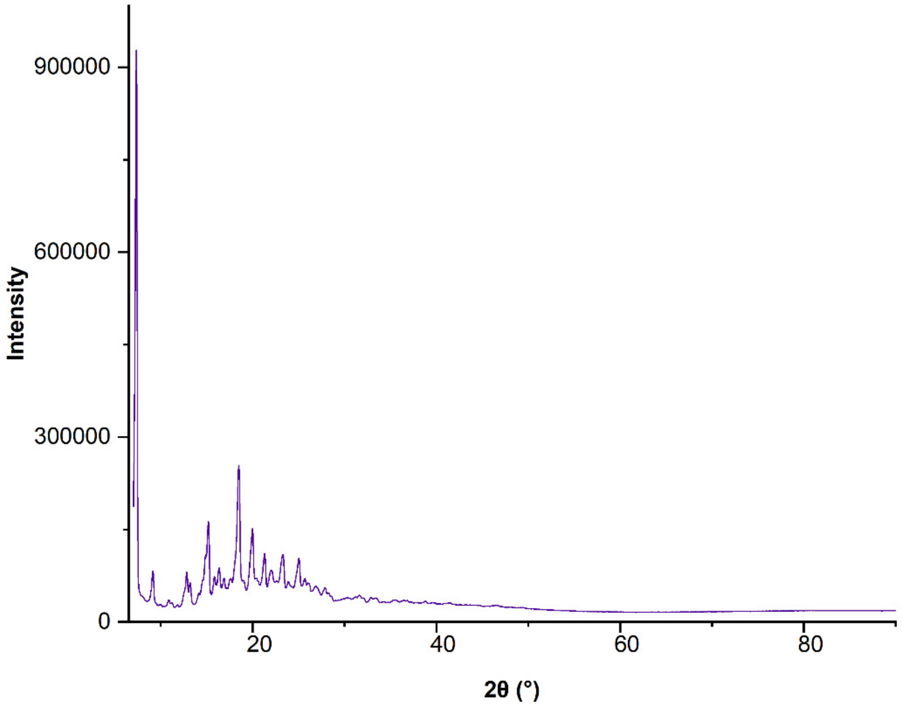

Figure S62. XRD pattern of [L-IleOiPr][SA].

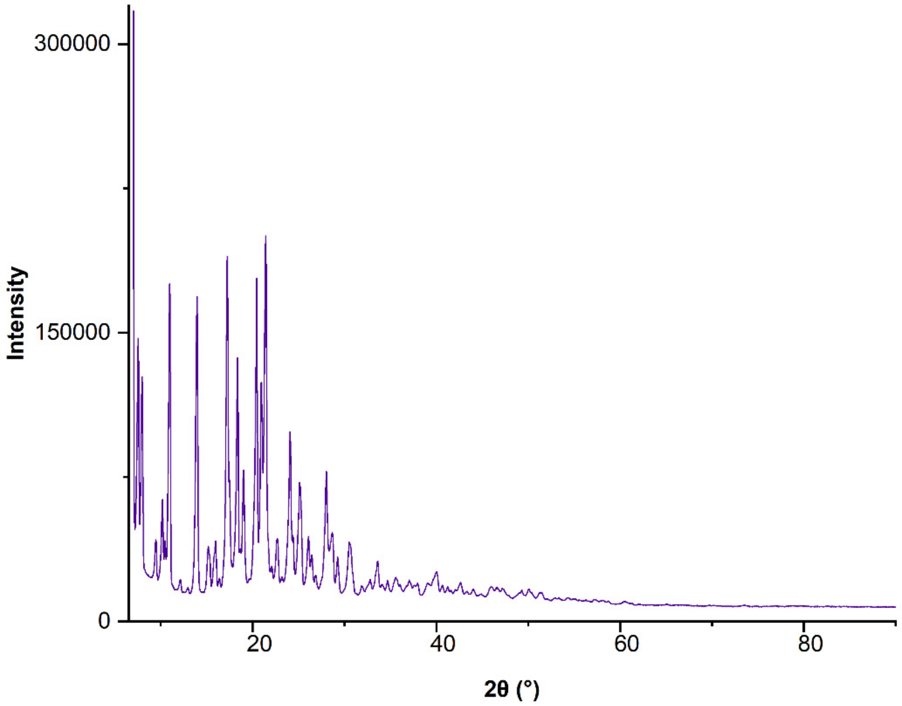

Figure S63. XRD pattern of [L-MetOiPr][SA].

The TG curves of [AAOiPr][KETO]

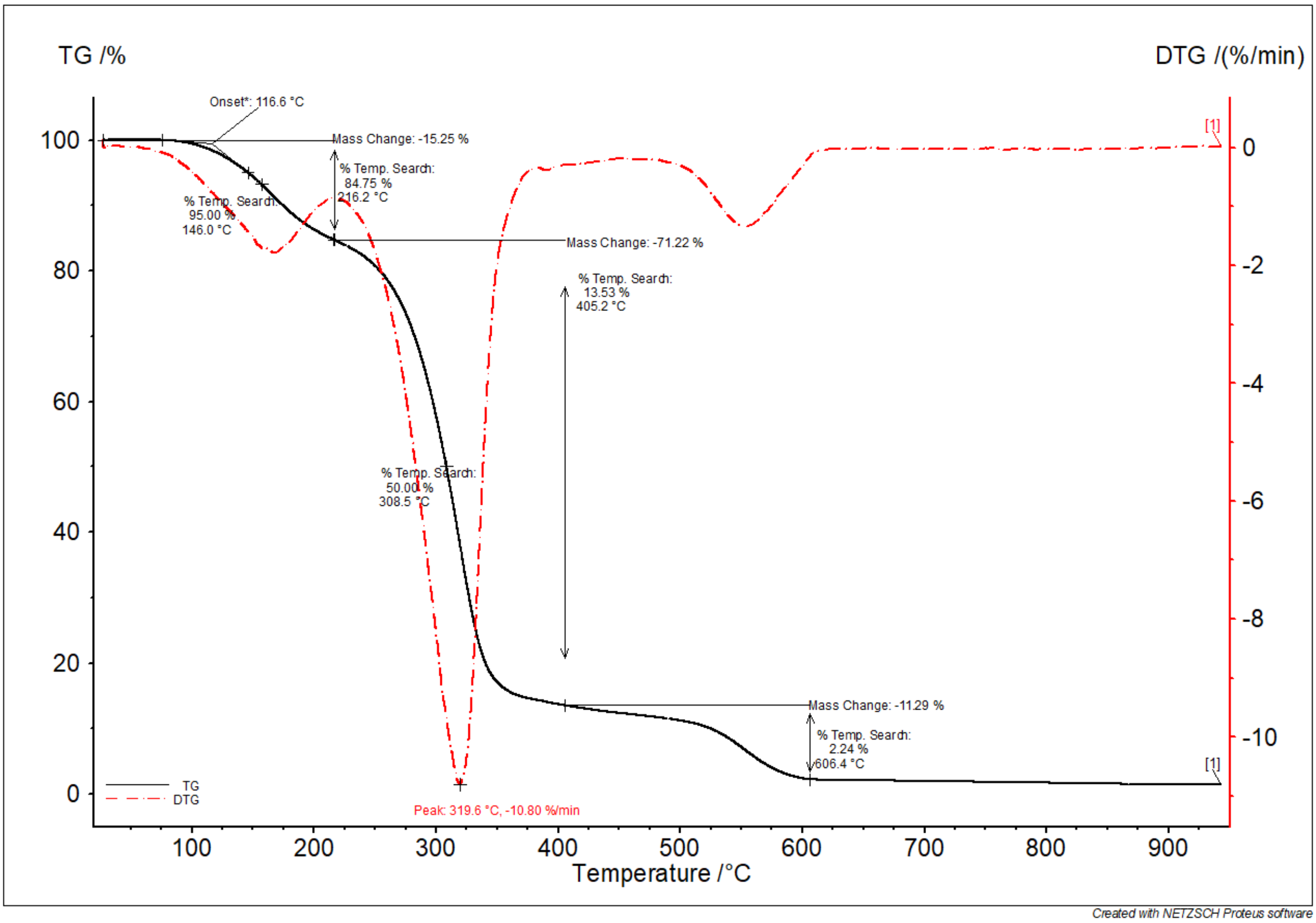

Figure S64. The TG and DTG curves of [L-IleOiPr][KETO].

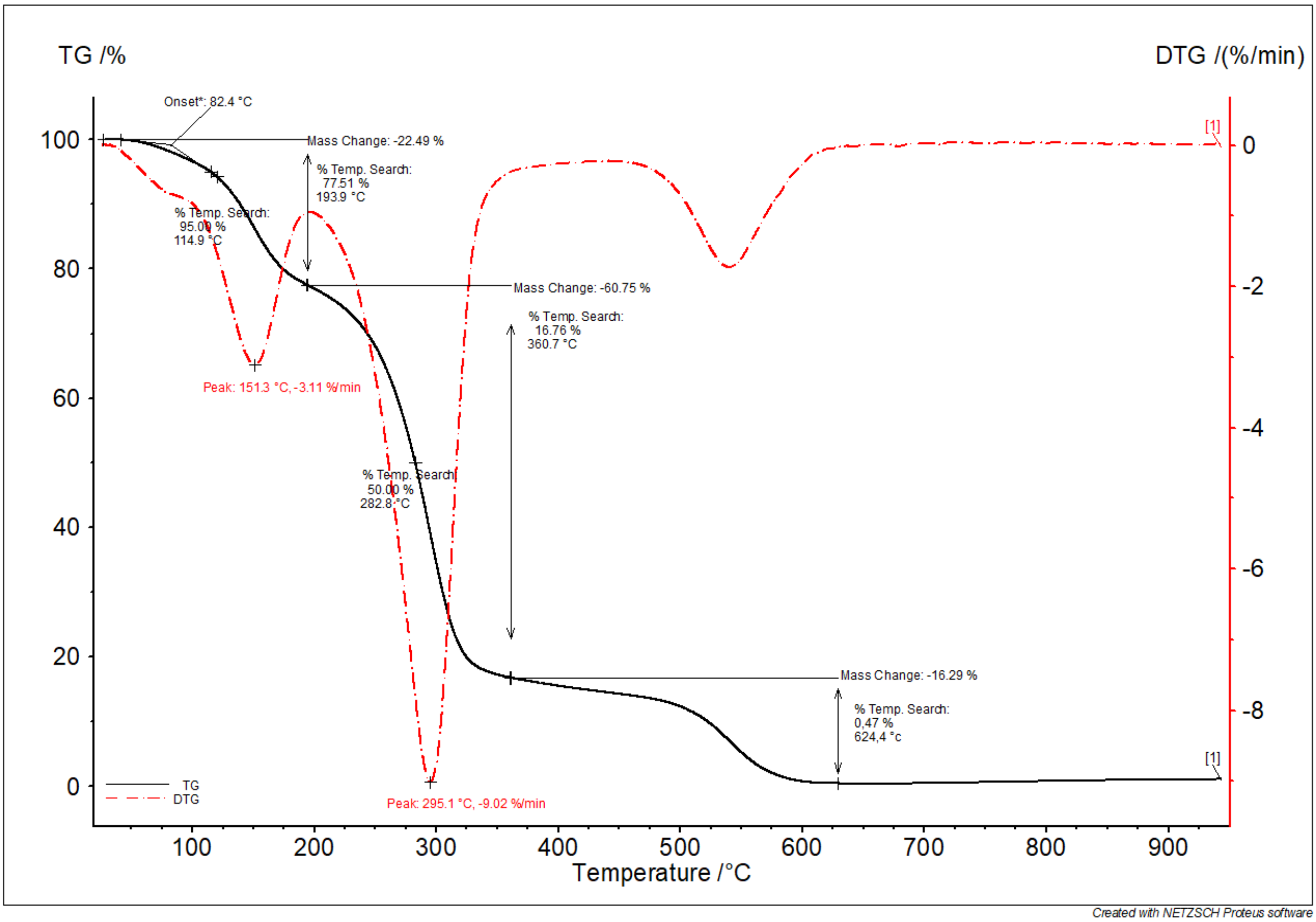

Figure S65. The TG and DTG curves of [L-ThrOiPr][KETO].

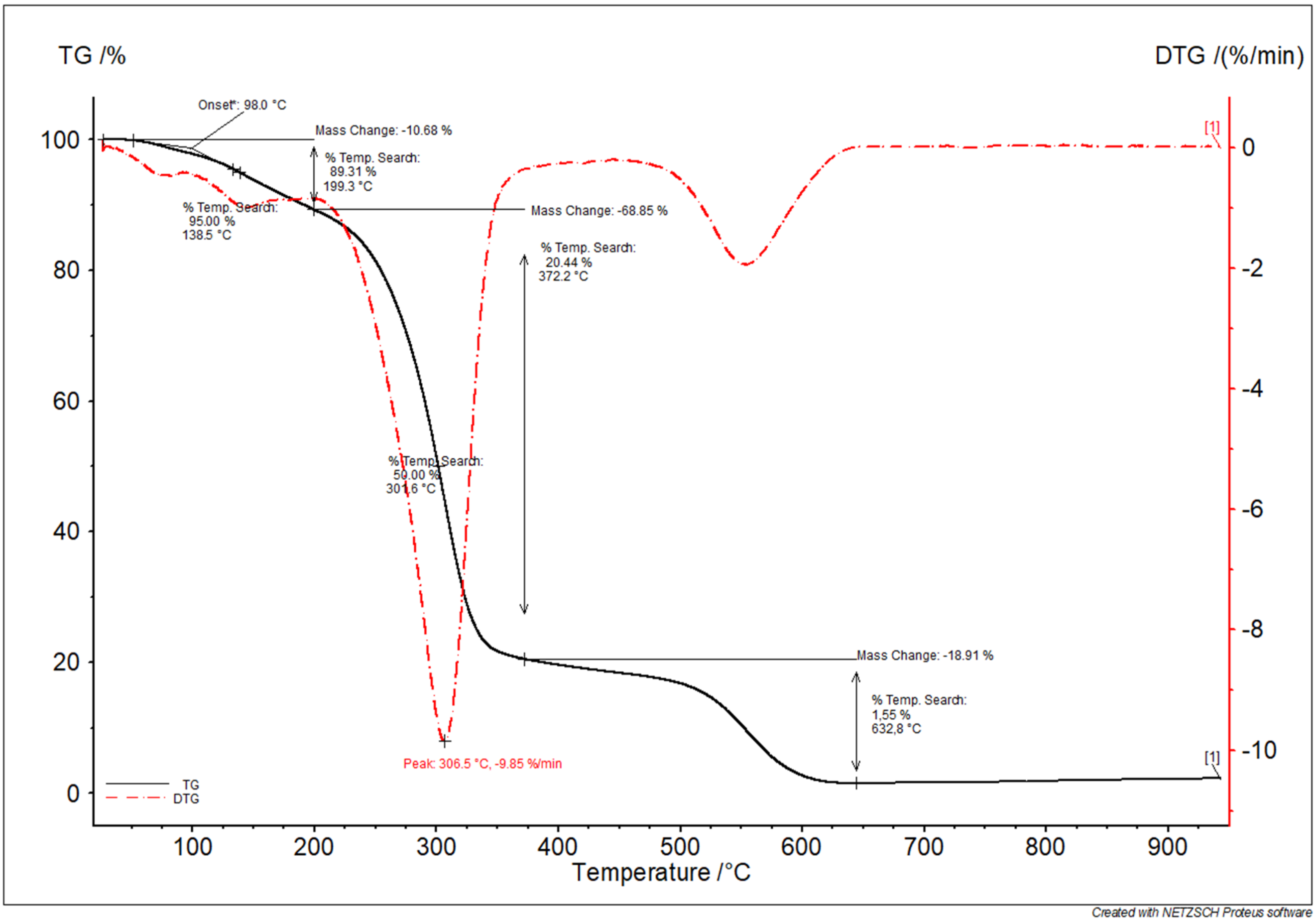

Figure S66. The TG and DTG curves of [L-MetOiPr][KETO].

The TG curves of [AAOiPr][NAP]

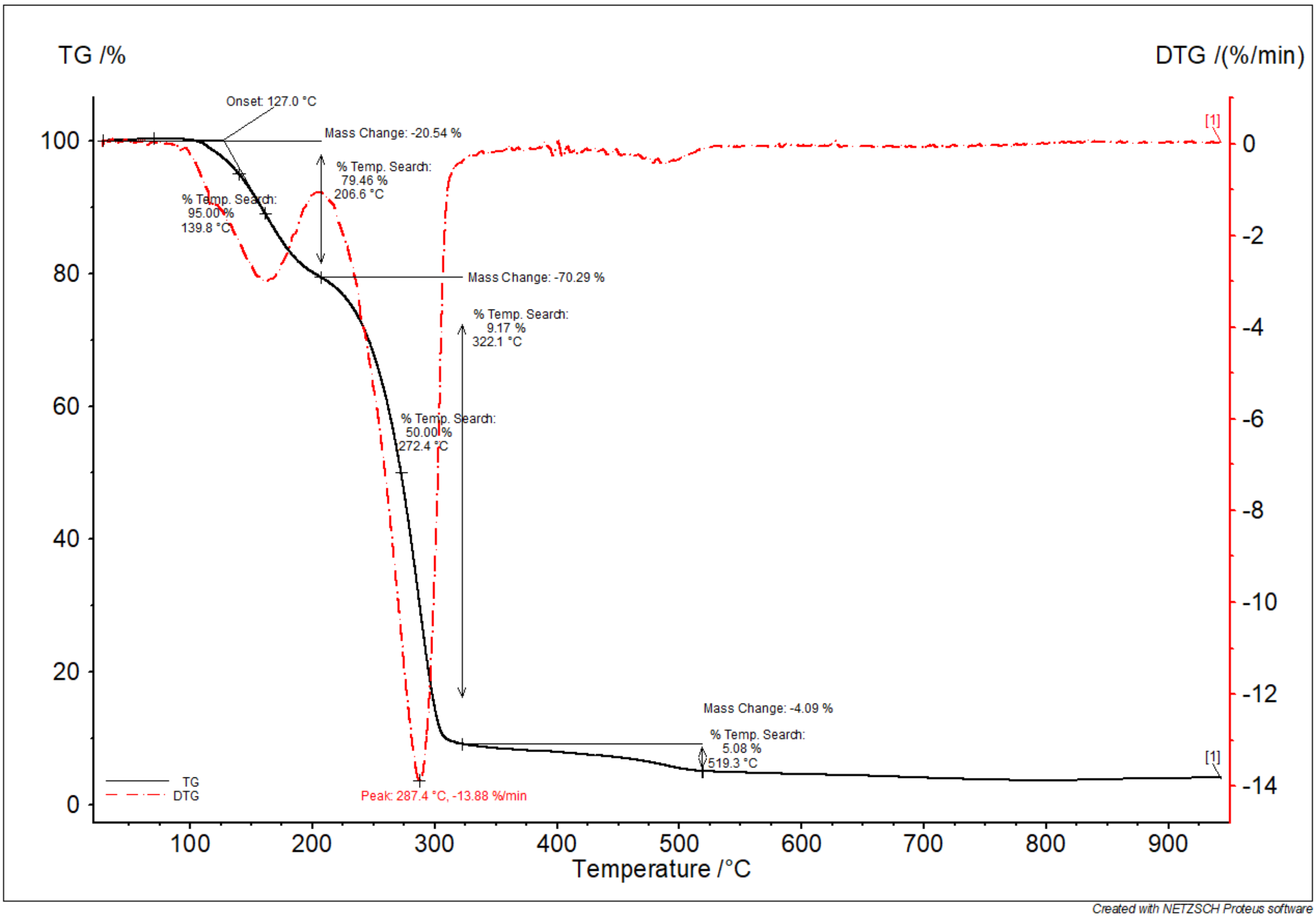

Figure S67. The TG and DTG curves of [L-IleOiPr][NAP].

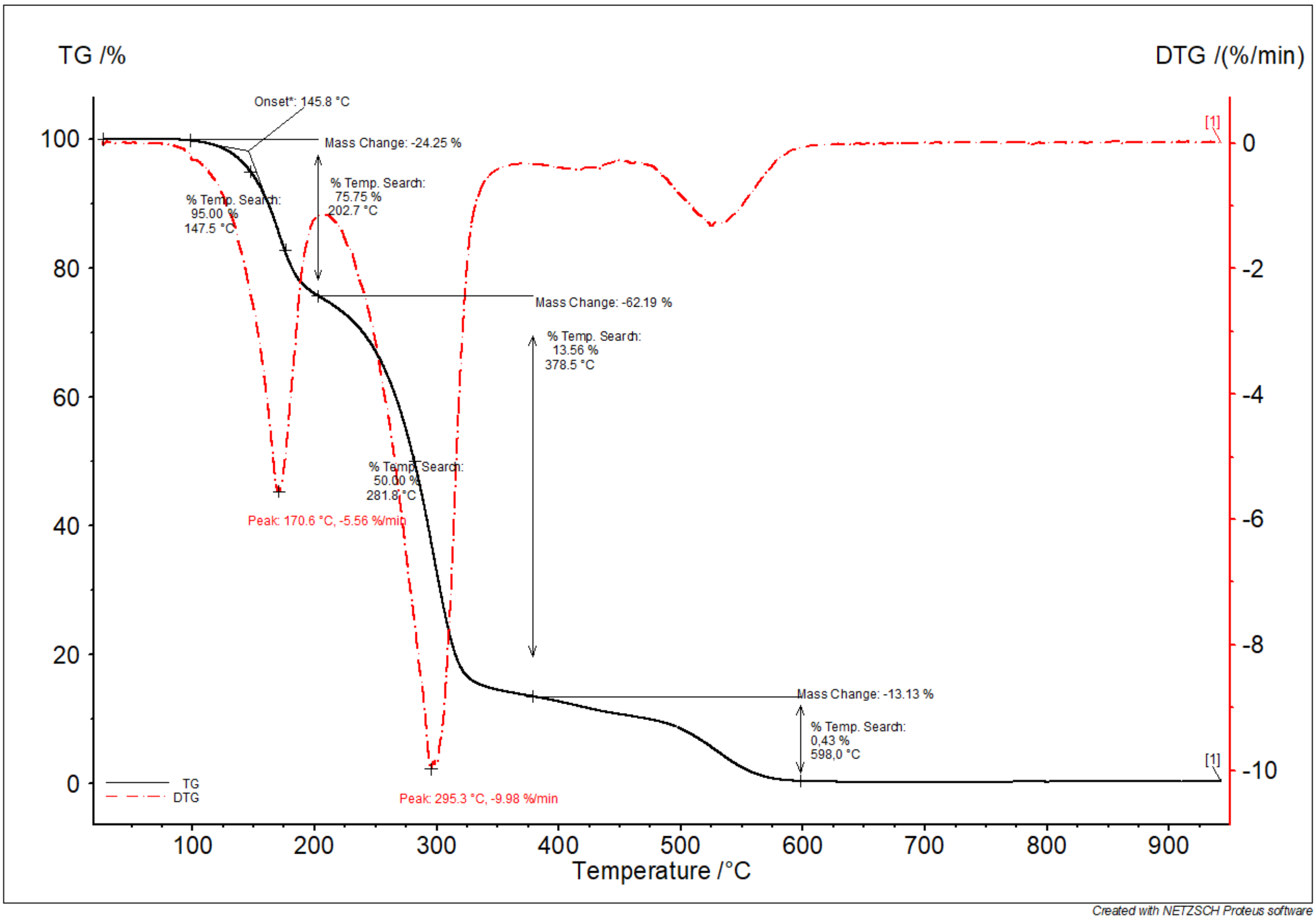

Figure S68. The TG and DTG curves of [L-ThrOiPr][NAP].

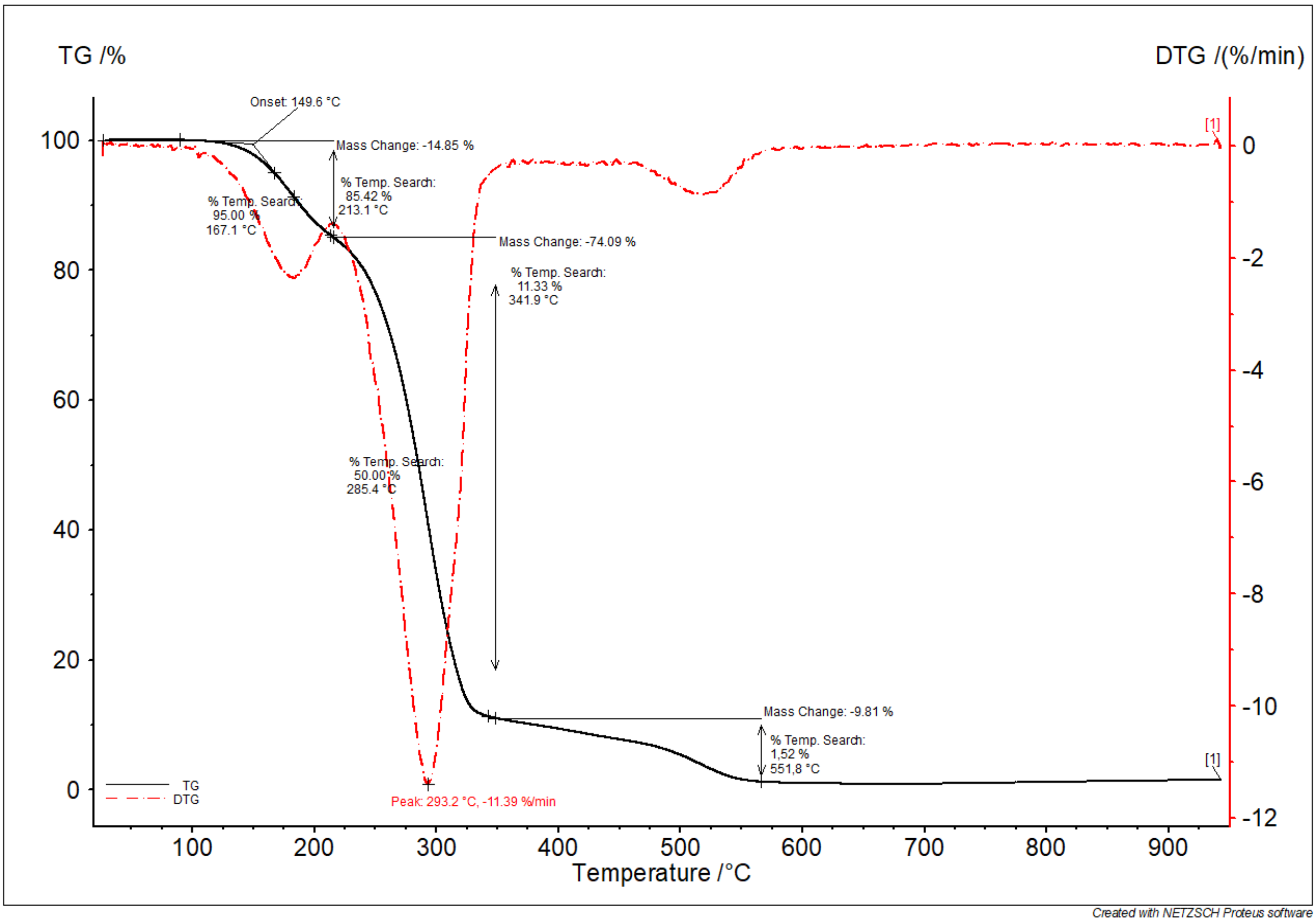

Figure S69. The TG and DTG curves of [L-MetOiPr][NAP].

The TG curves of [AAOiPr][SA]

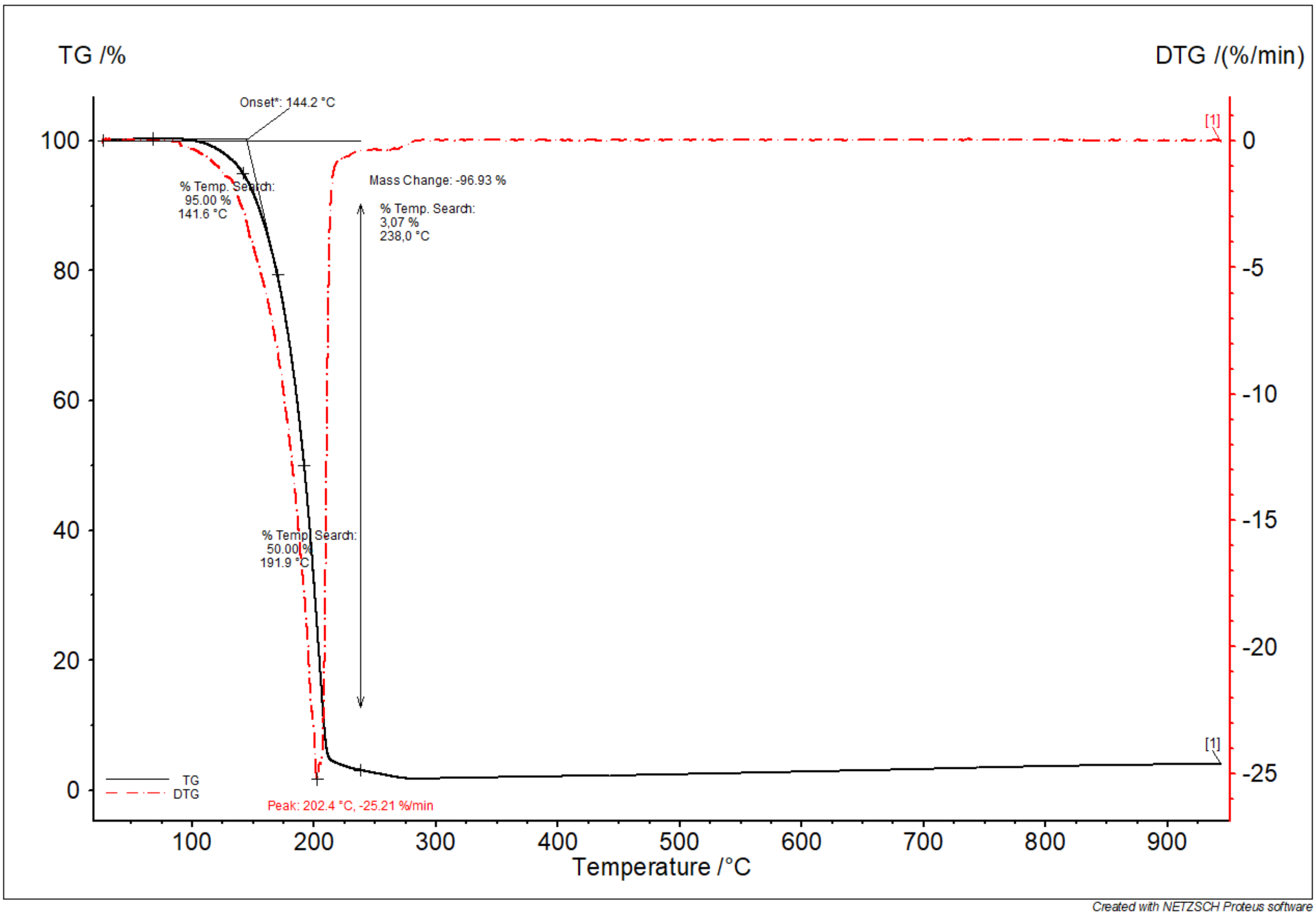

Figure S70. The TG and DTG curves of [L-IleOiPr][SA].

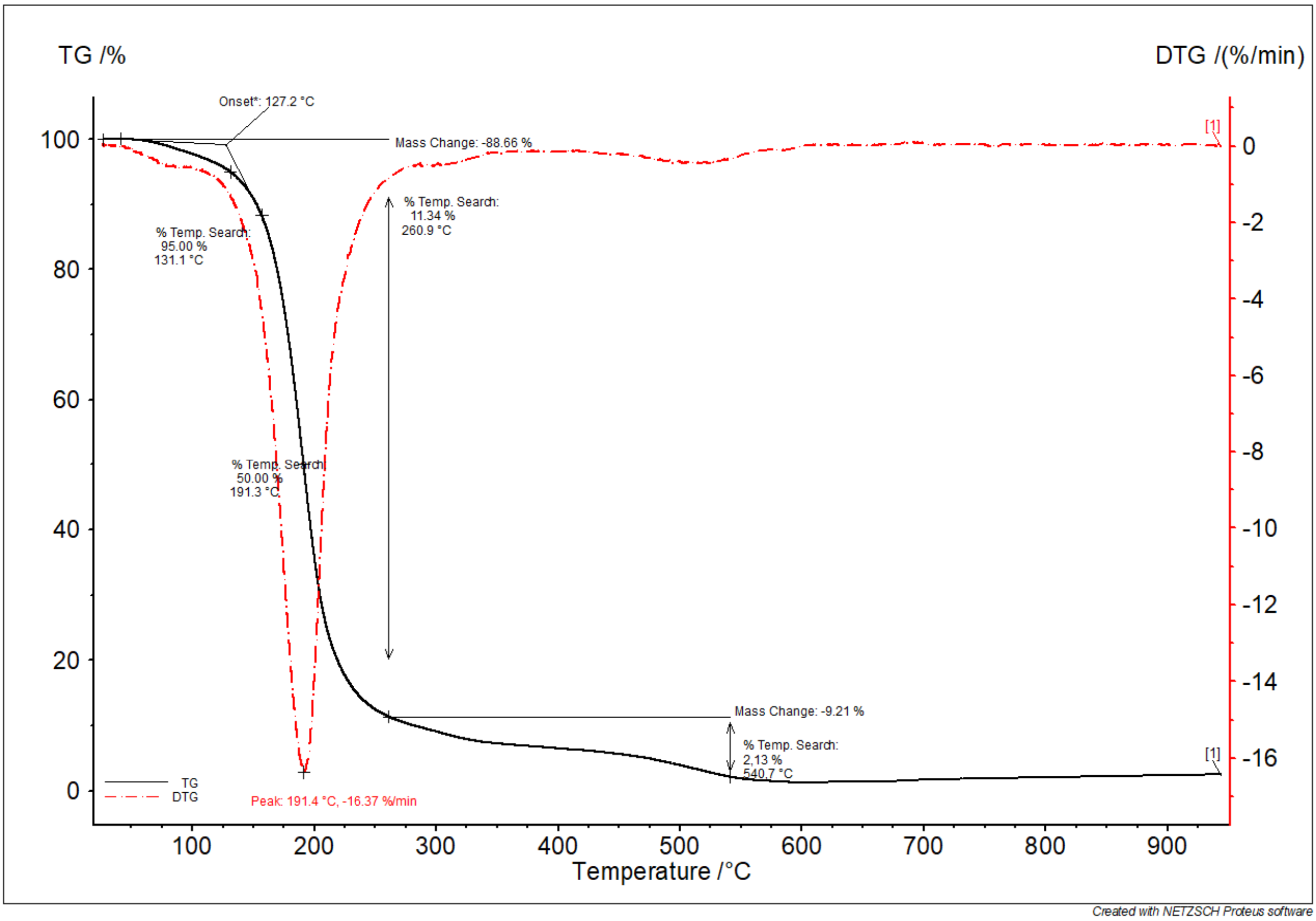

Figure S71. The TG and DTG curves of [L-ThrOiPr][SA].

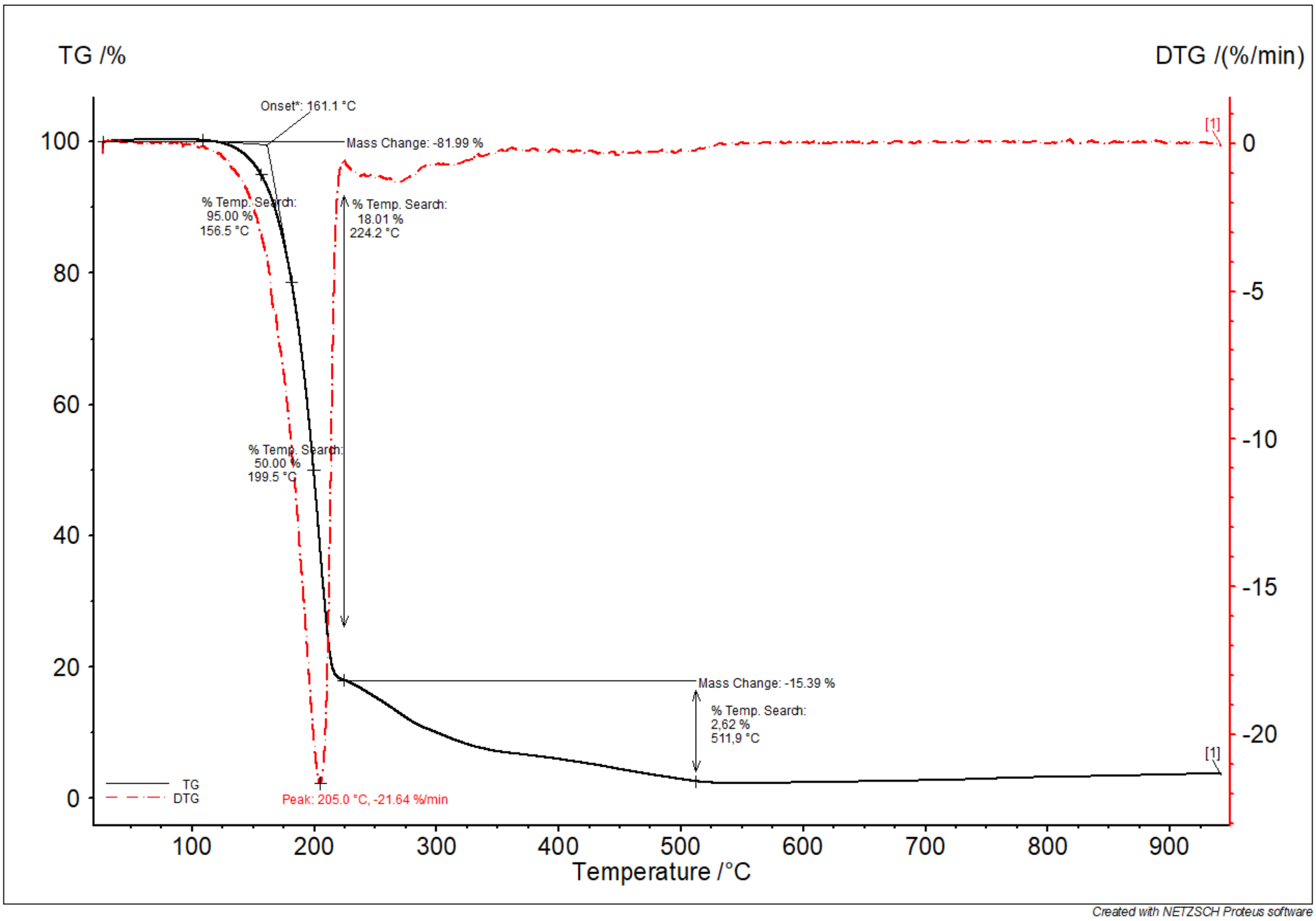

Figure S72. The TG and DTG curves of [L-MetOiPr][SA].

The DSC curves of [AAOiPr][IBU]

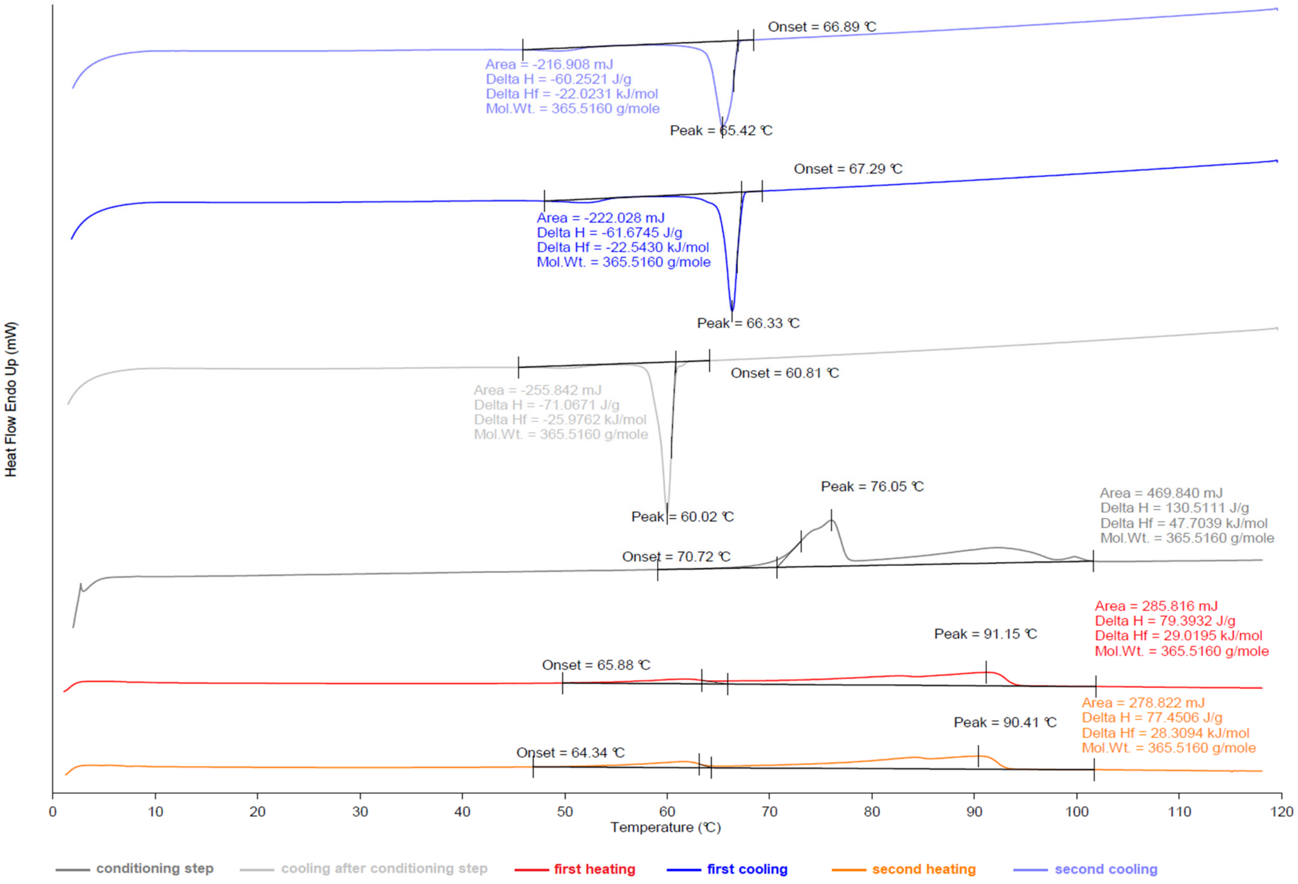

Figure S73. The DSC curves of [L-ValOiPr][IBU].

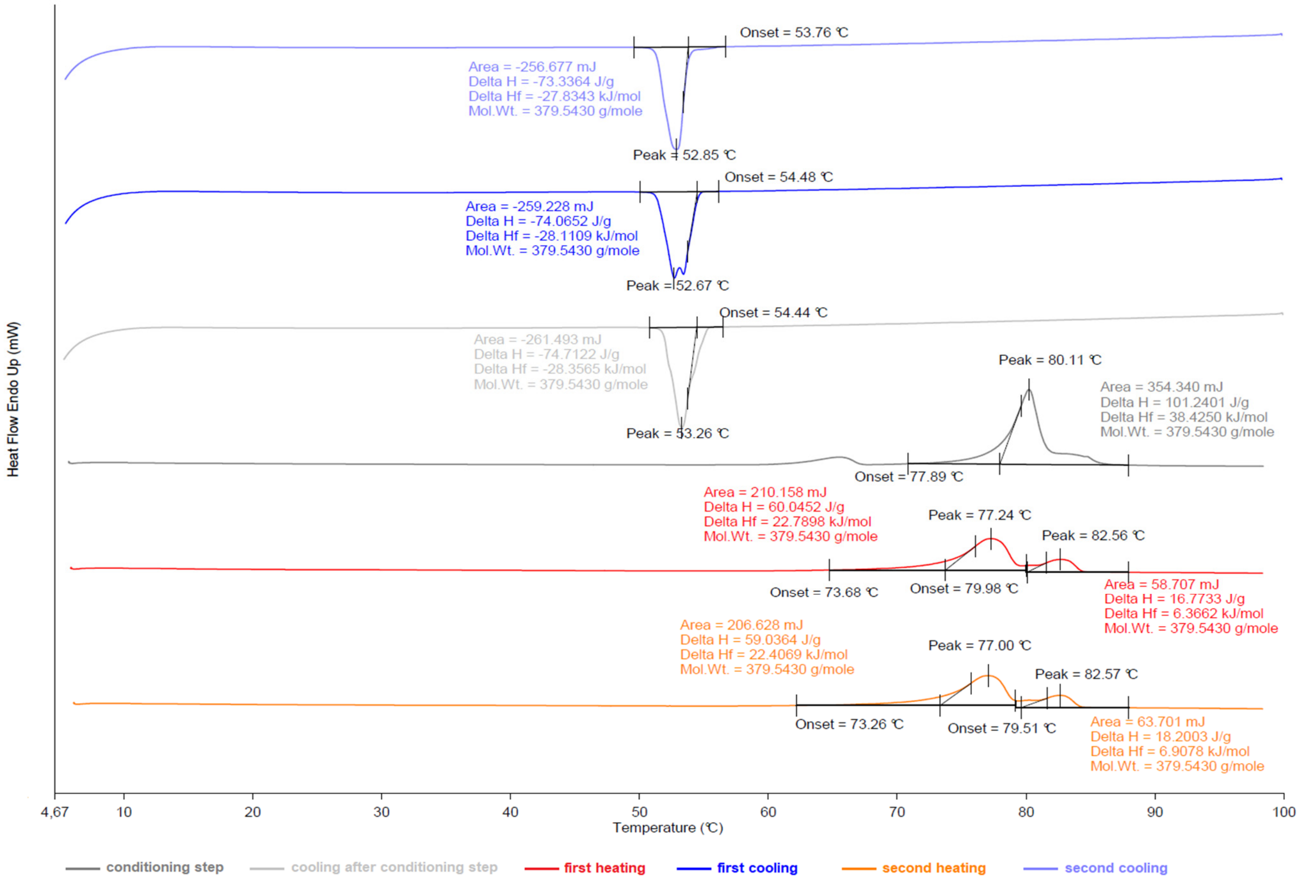

Figure S74. The DSC curves of [L-IleOiPr][IBU].

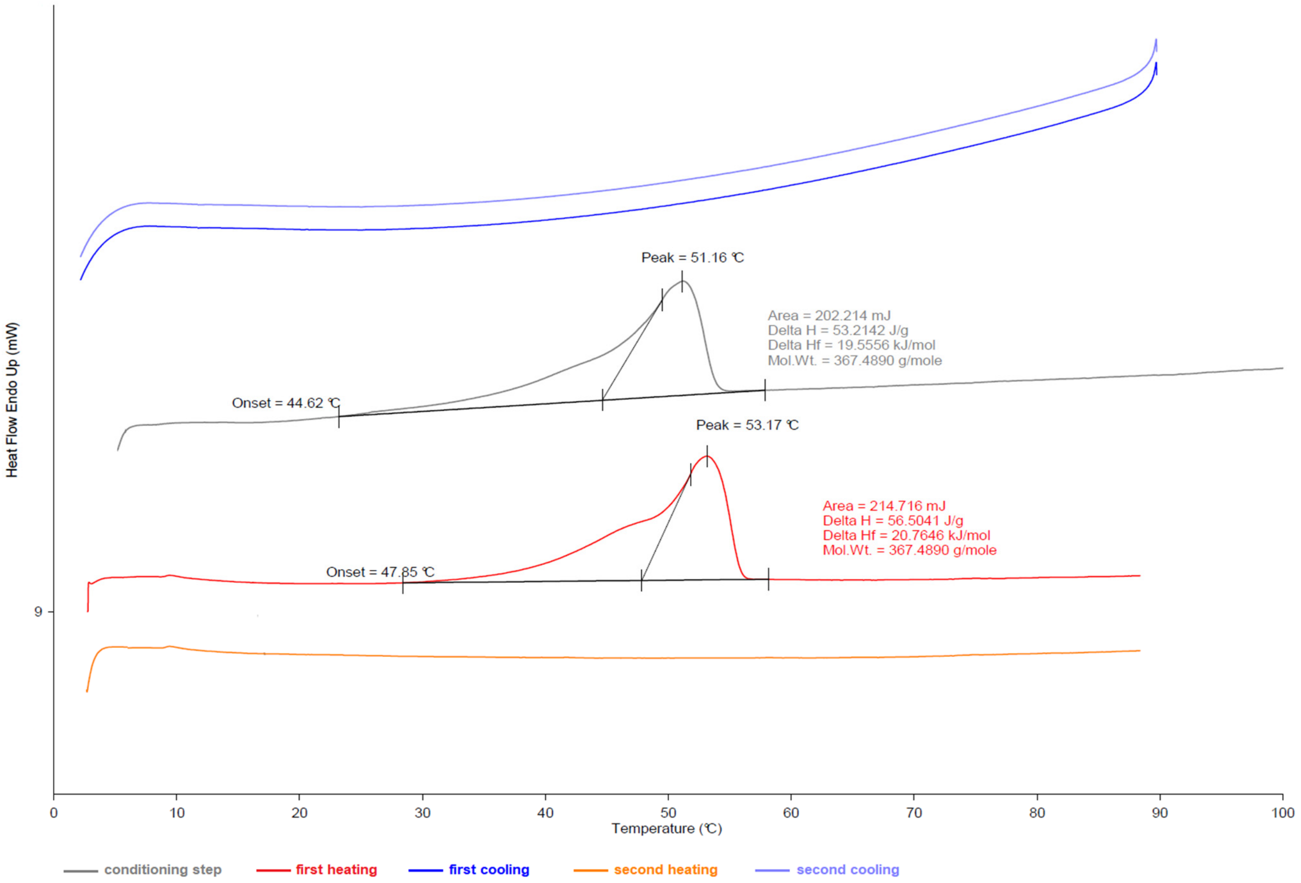

Figure S75. The DSC curves of [L-ThrOiPr][IBU].

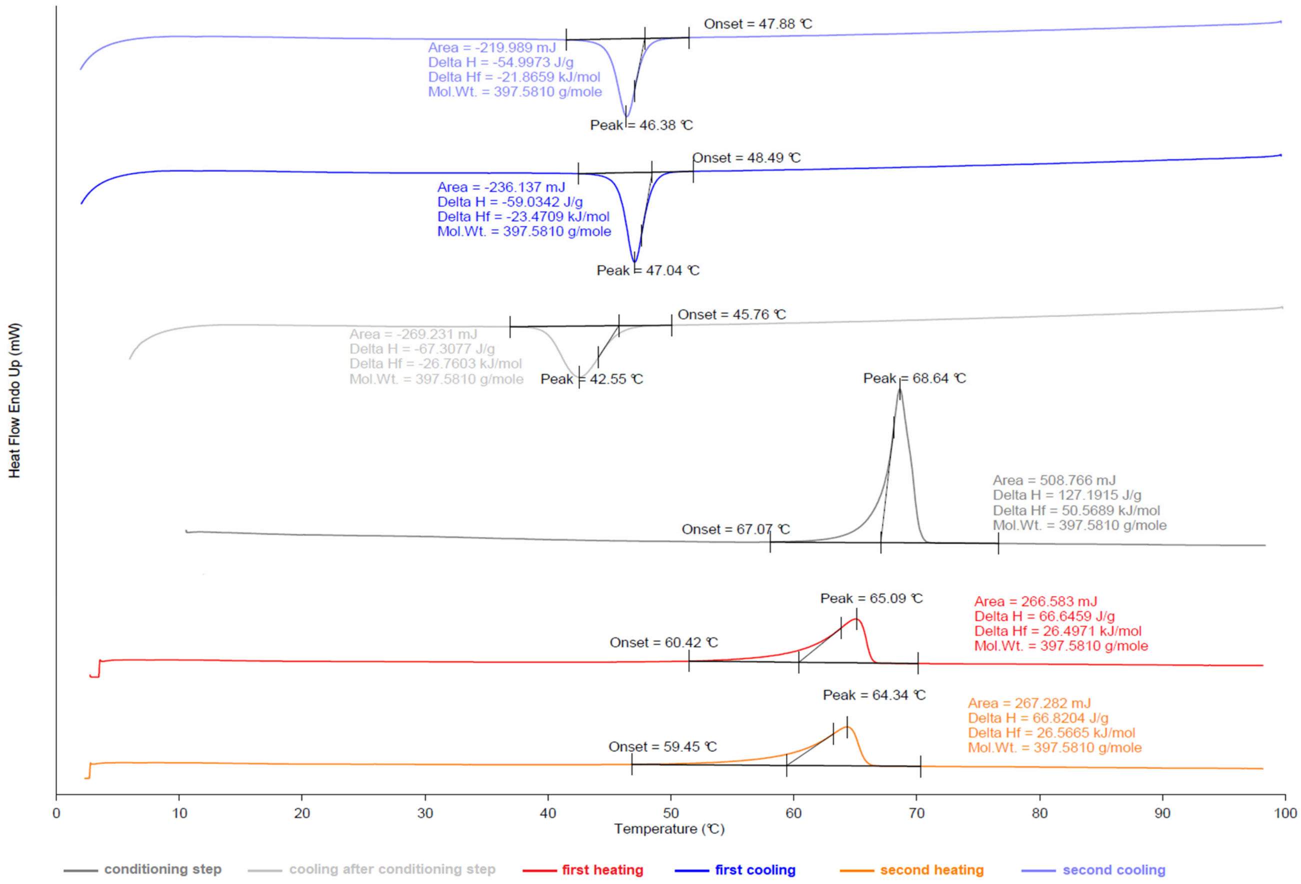

Figure S76. The DSC curves of [L-MetOiPr][IBU].

The DSC curves of [AAOiPr][KETO]

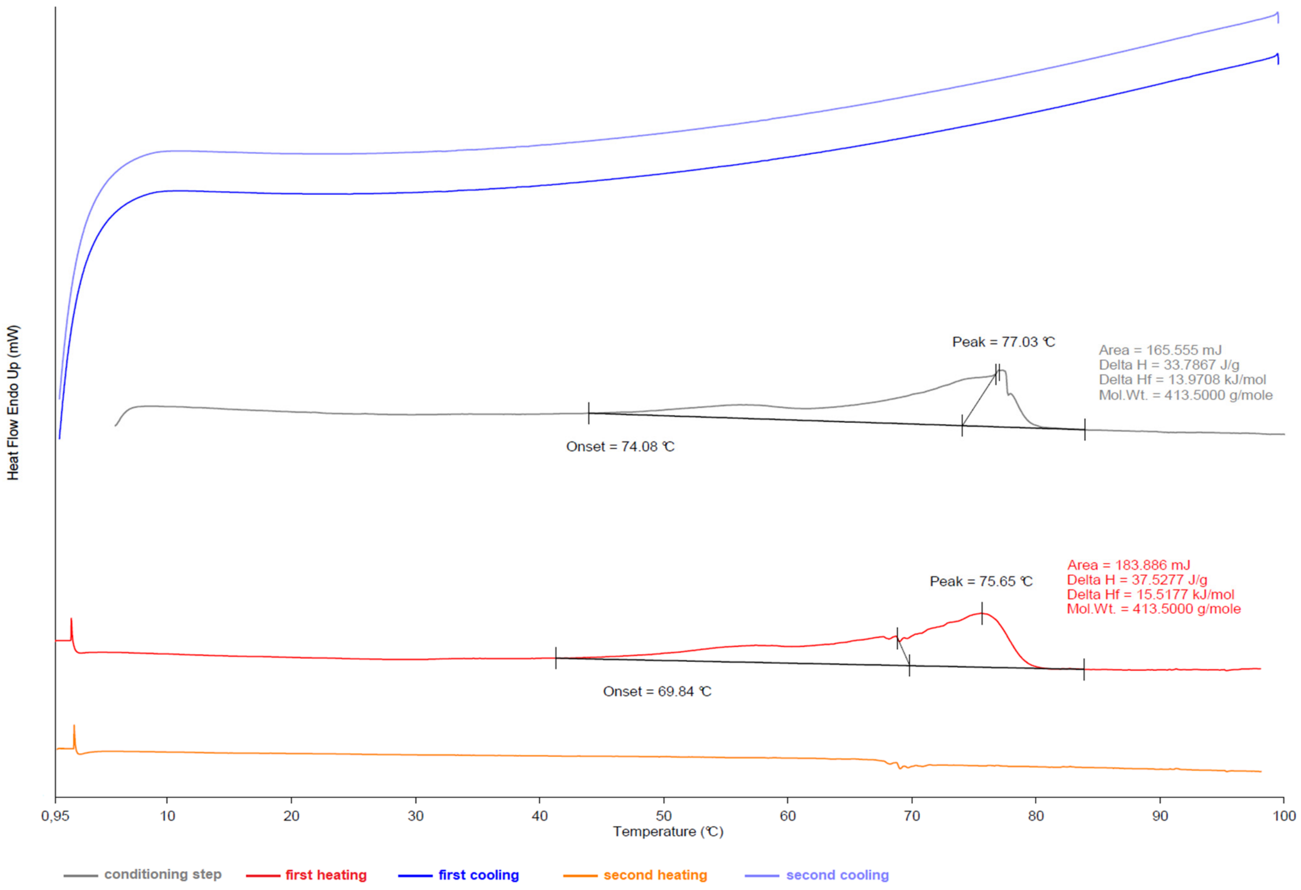

Figure S77. The DSC curves of [L-ValOiPr][KETO].

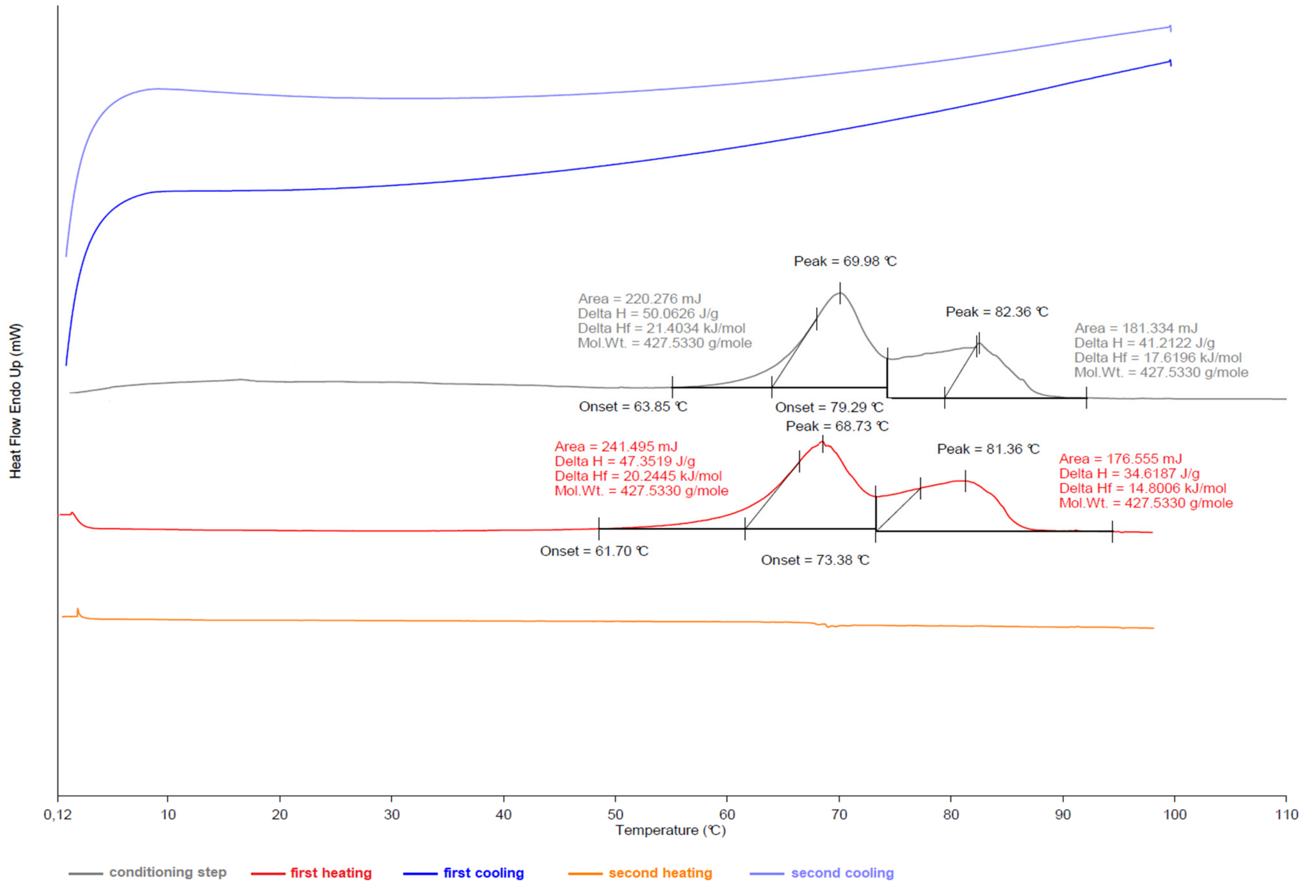

Figure S78. The DSC curves of [L-IleOiPr][KETO].

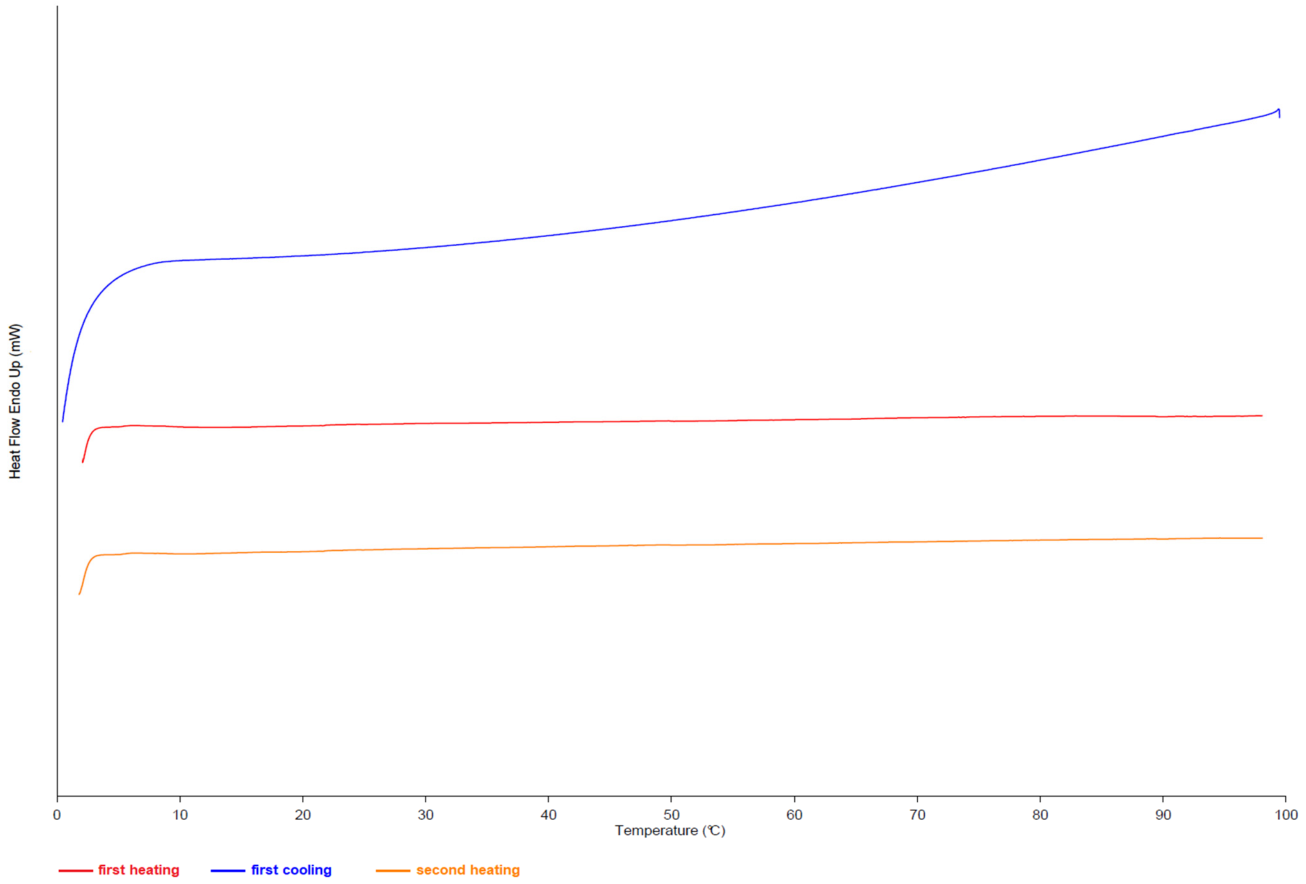

Figure S79. The DSC curves of [L-ThrOiPr][KETO].

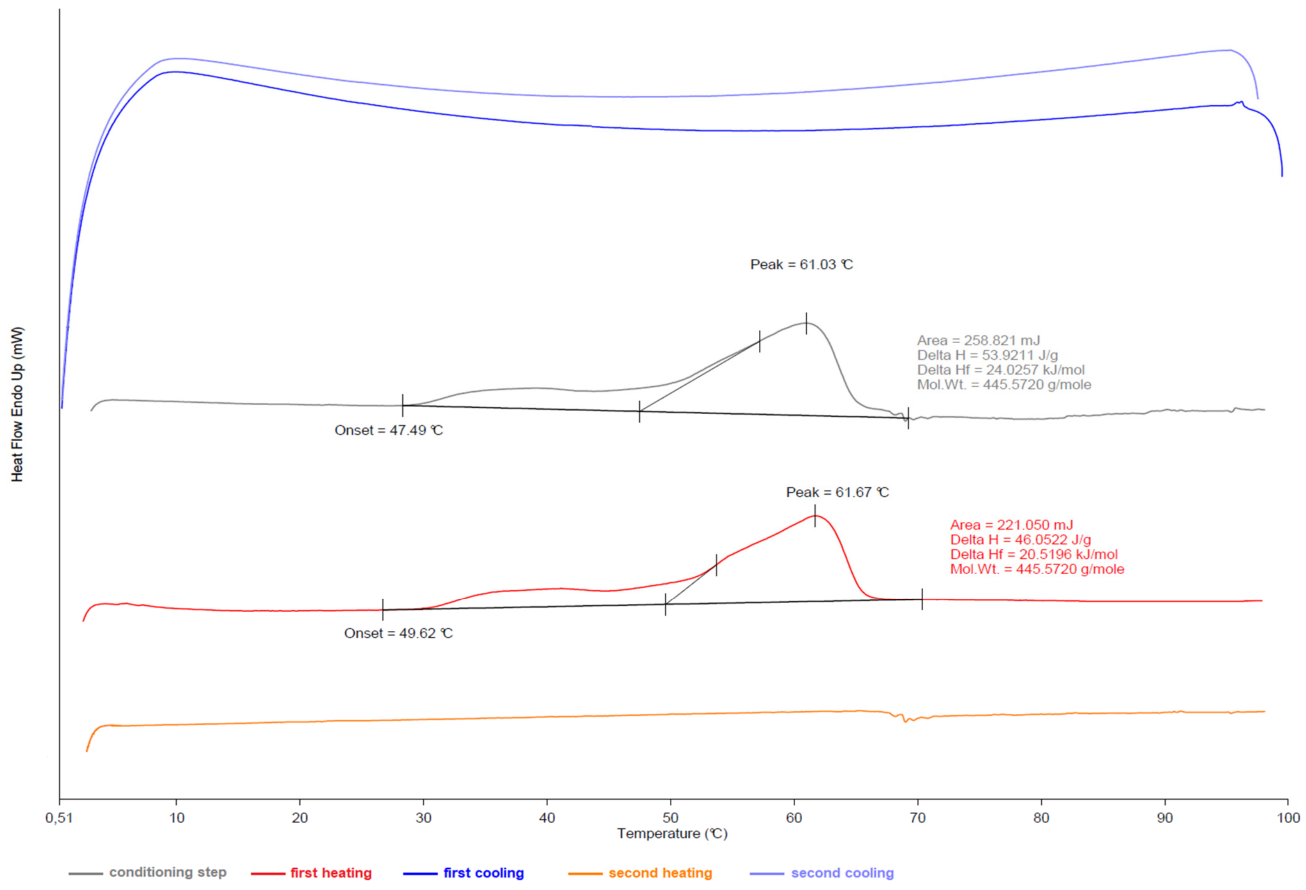

Figure S80. The DSC curves of [L-MetOiPr][KETO].

The DSC curves of [AAOiPr][NAP]

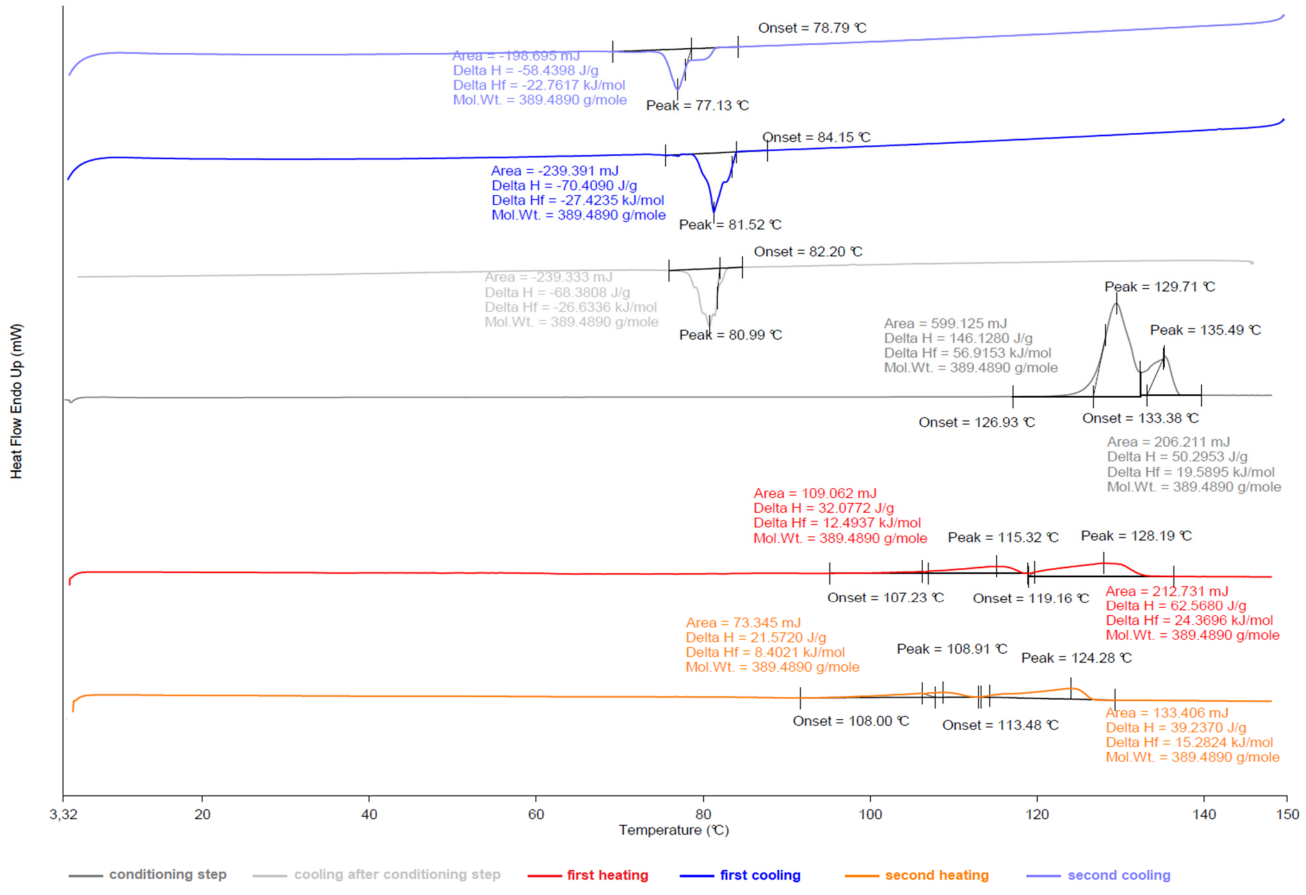

Figure S81. The DSC curves of [L-ValOiPr][NAP].

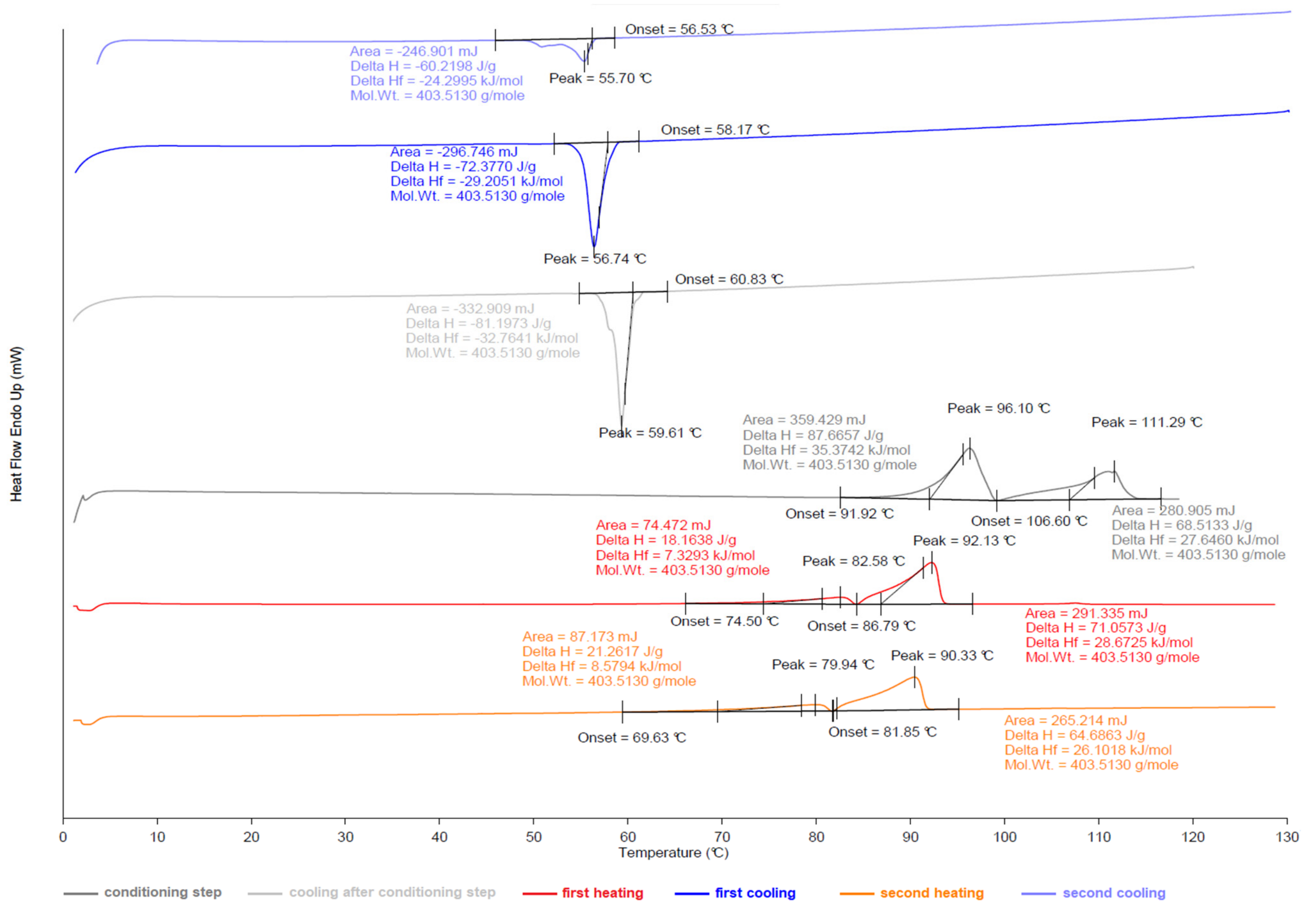

Figure S82. The DSC curves of [L-IleOiPr][NAP].

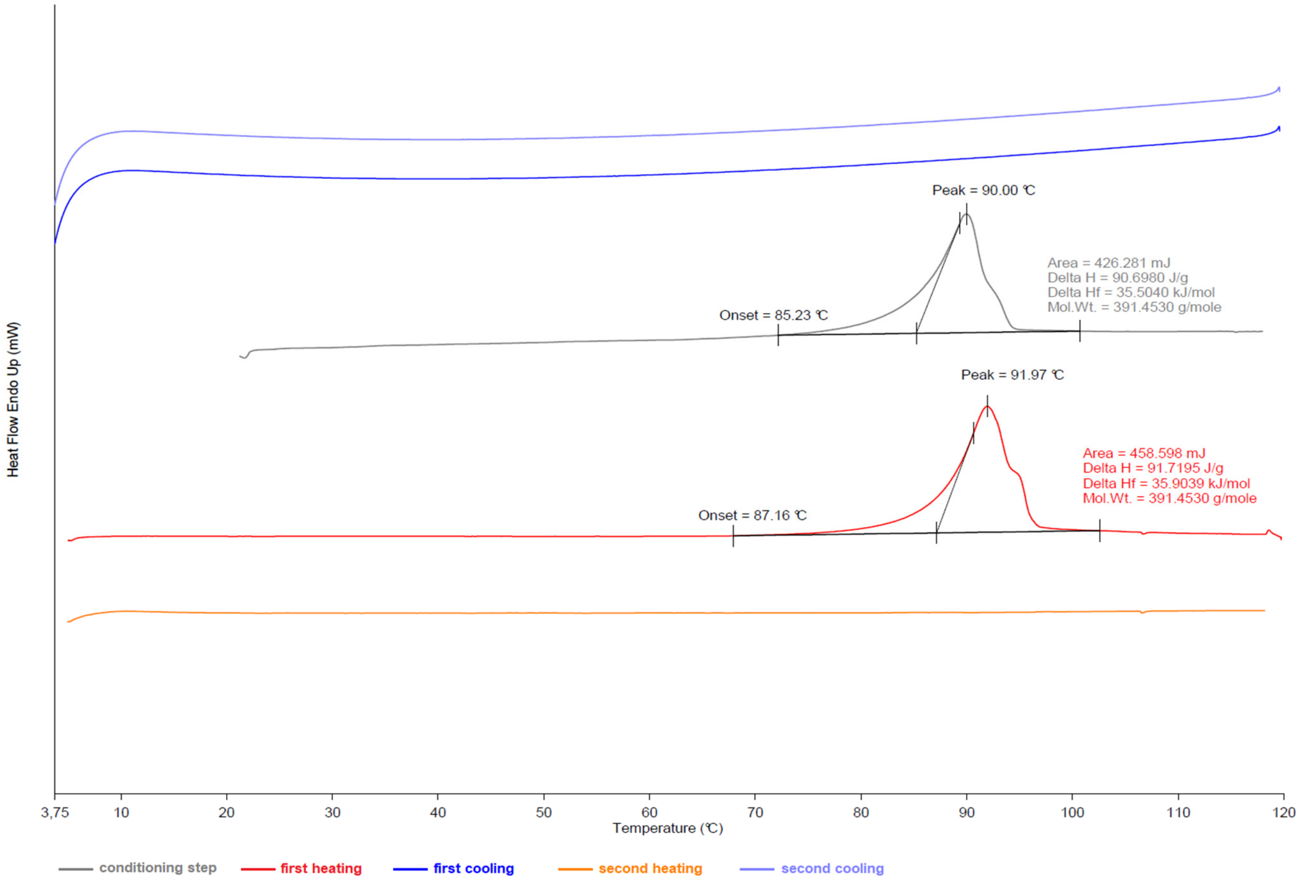

Figure S83. The DSC curves of [L-ThrOiPr][NAP].

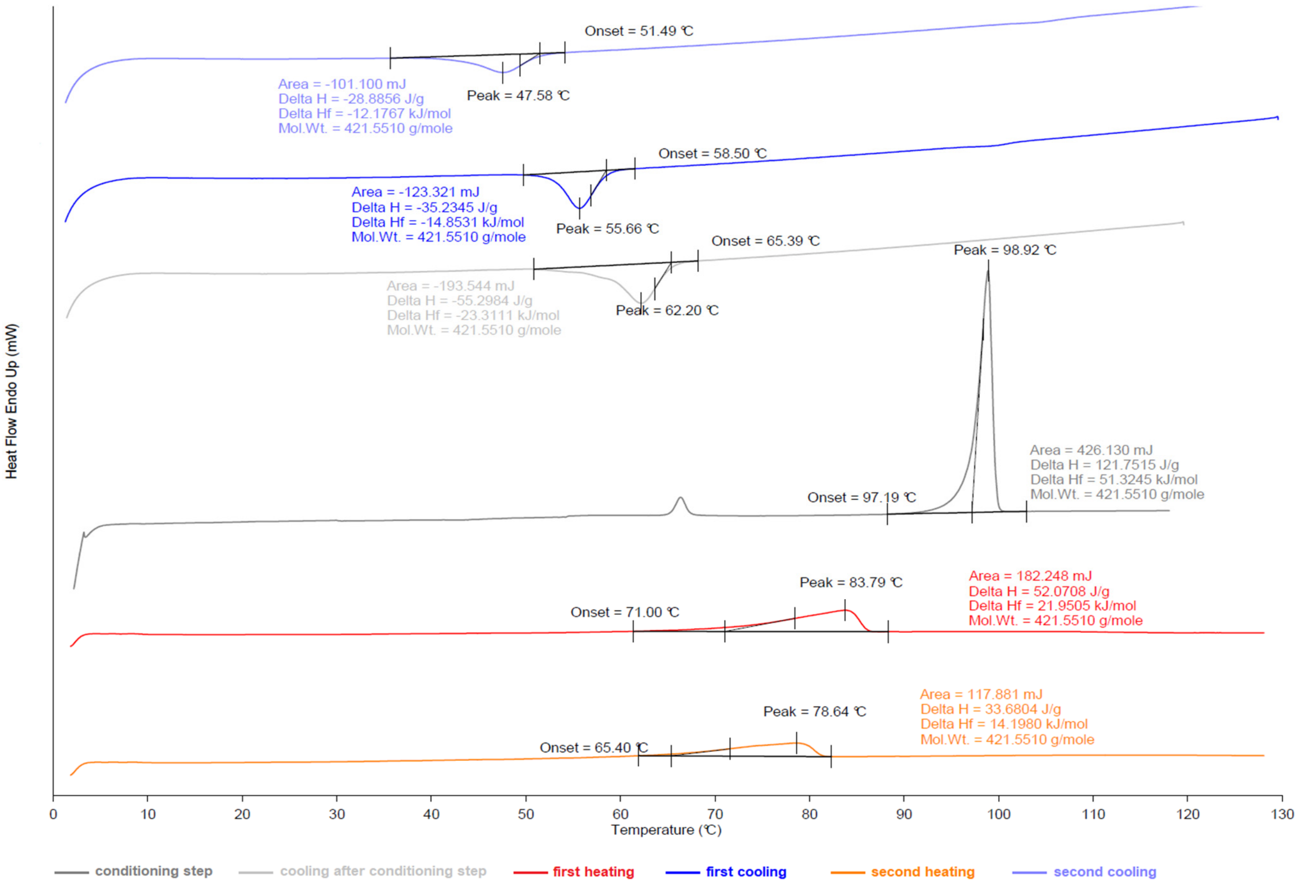

Figure S84. The DSC curves of [L-MetOiPr][NAP].

The DSC curves of [AAOiPr][SA]

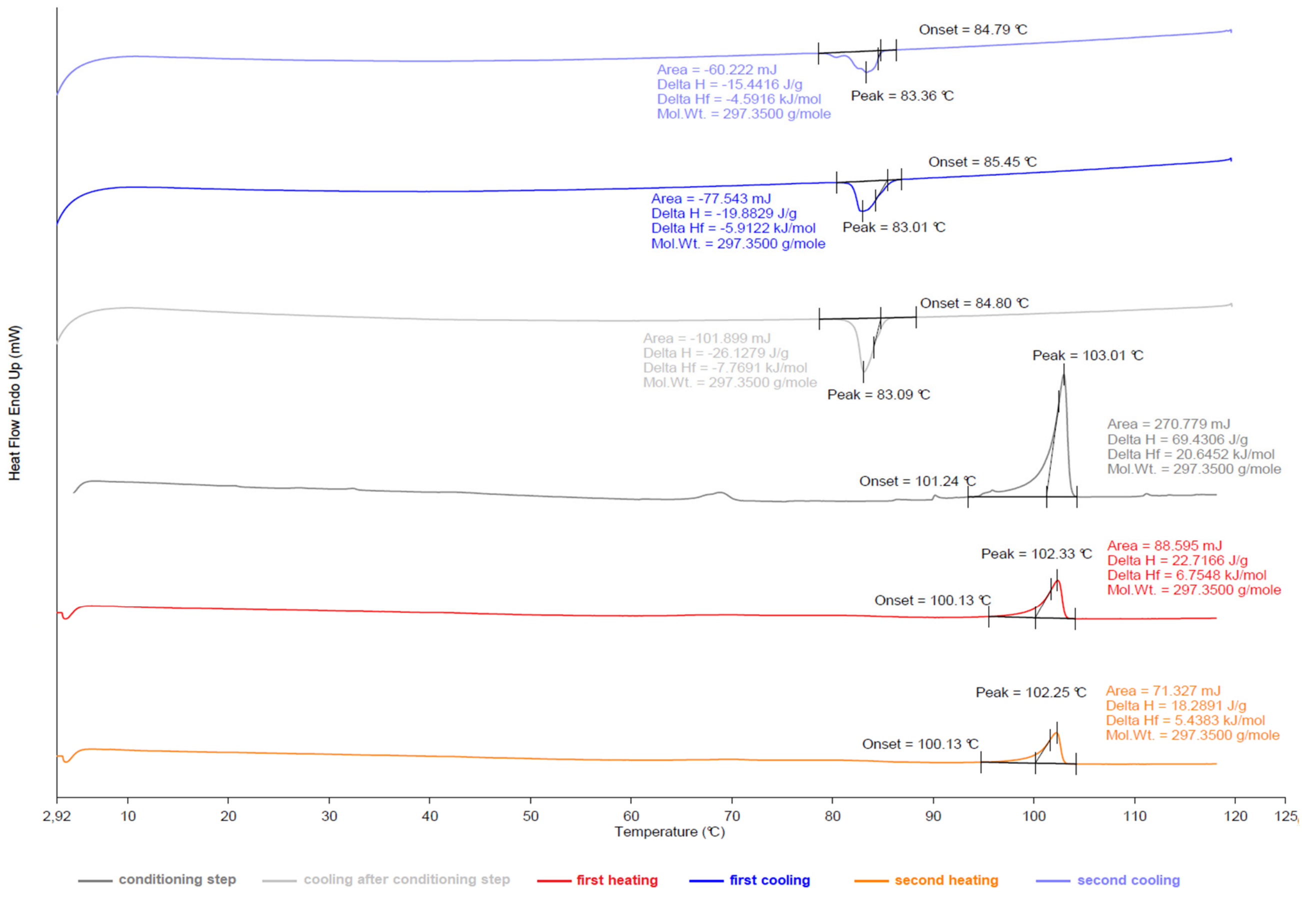

Figure S85. The DSC curves of [L-ValOiPr][SA].

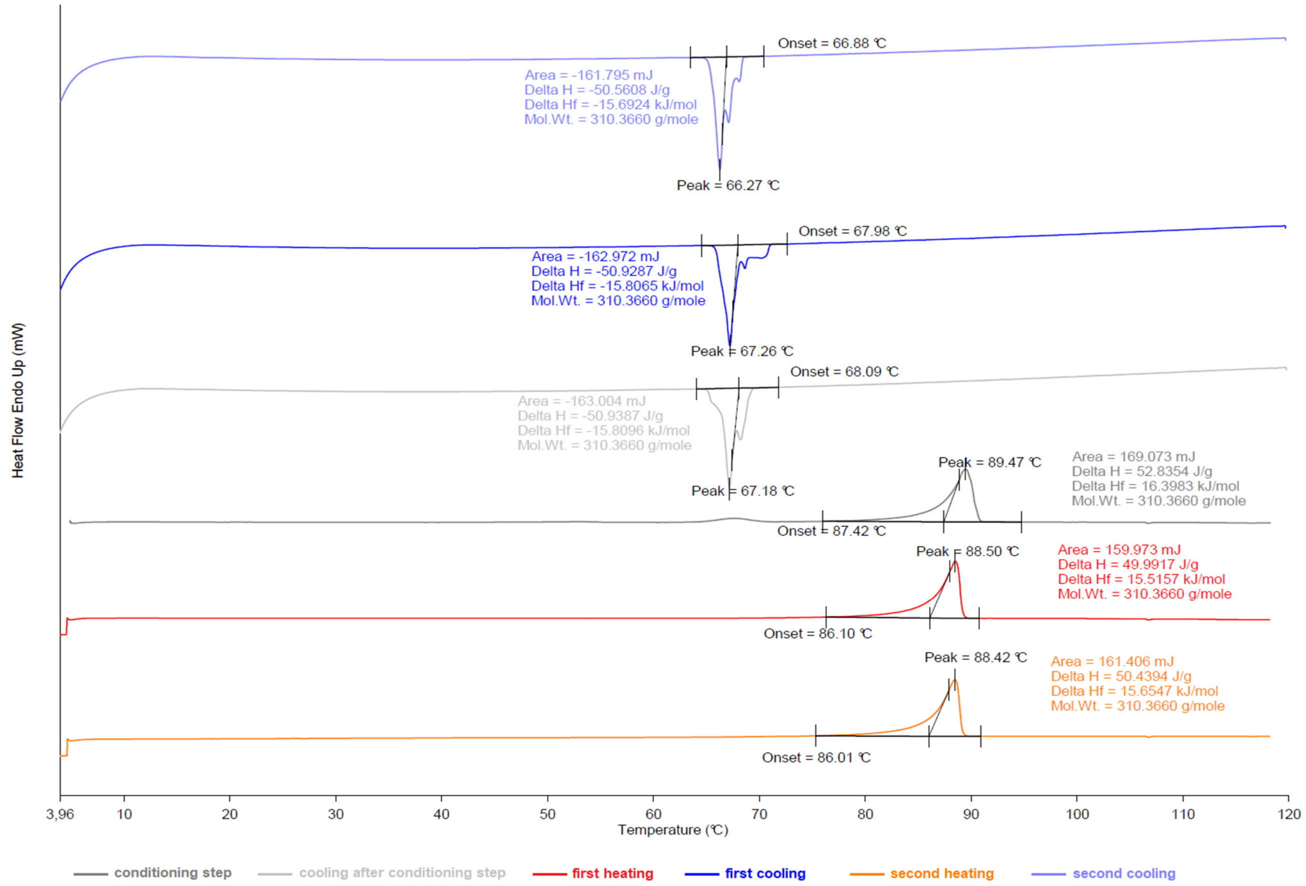

Figure S86. The DSC curves of [L-IleOiPr][SA].

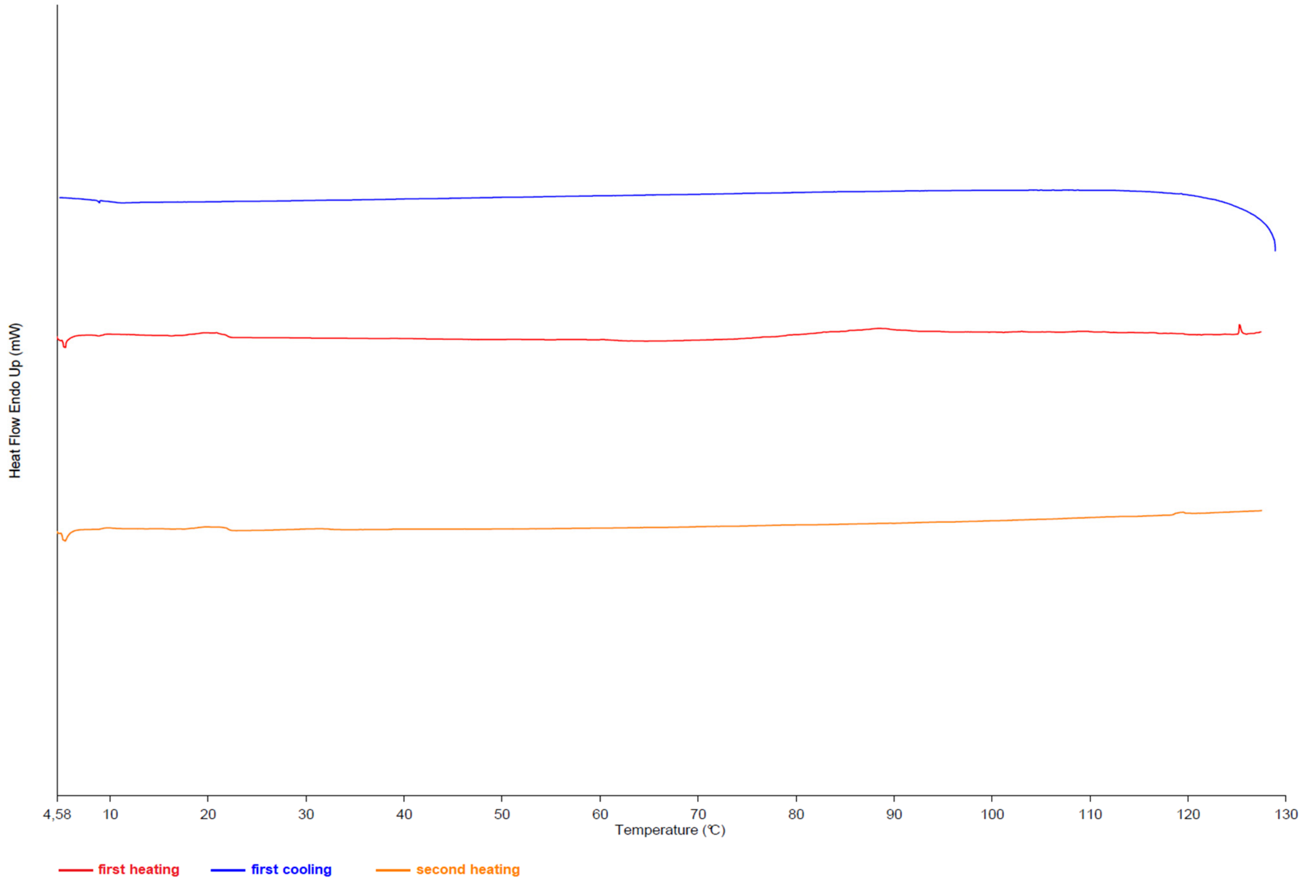

Figure S87. The DSC curves of [L-ThrOiPr][SA].

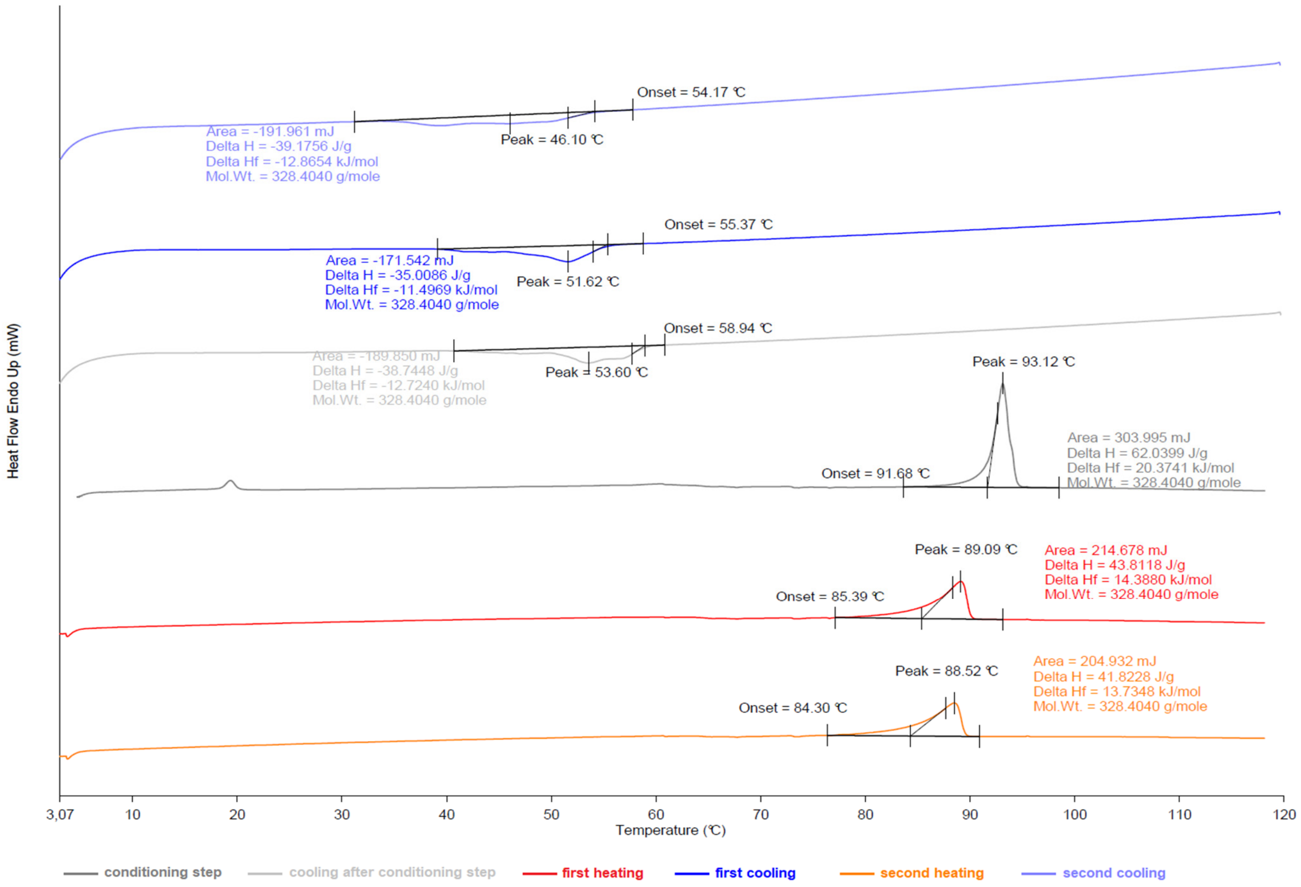

Figure S88. The DSC curves of [L-MetOiPr][SA].
